# Supplementary material for: Pseudohypoxic HIF pathway activation dysregulates collagen structure-function in human lung fibrosis
Source: eLife. 2022 Feb 21;11:e69348. doi: 10.7554/eLife.69348 (PMC8860444; doi:10.7554/eLife.69348)
Supplement: Figure 2—source data 1. [file elife-69348-fig2-data1.zip › Figure 2C-source data 1/Figure 2C labelled.pptx]

## Slide 1
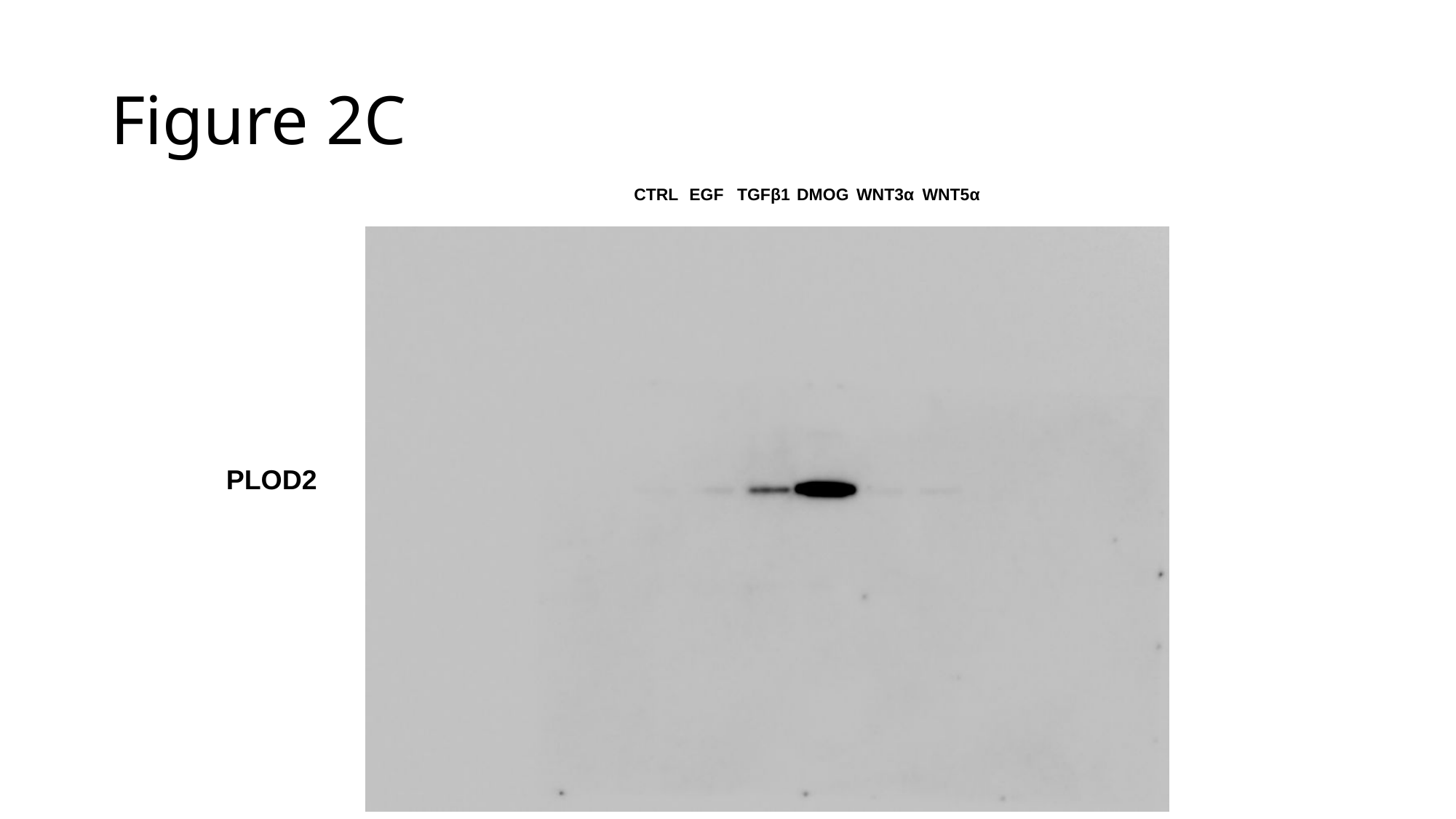

# Figure 2C
CTRL
TGFβ1
WNT3α
WNT5α
EGF
DMOG
PLOD2

## Slide 2
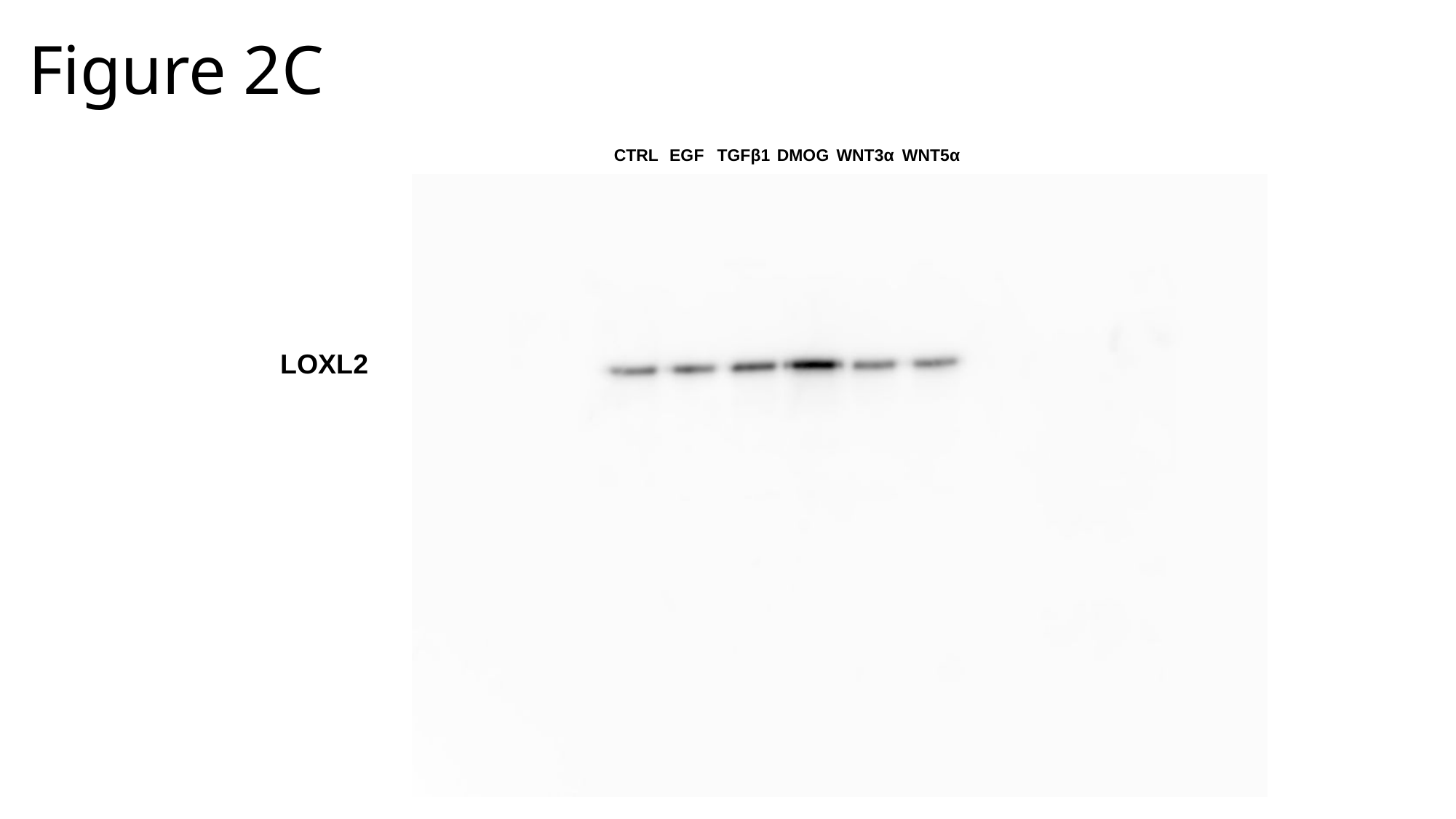

# Figure 2C
CTRL
TGFβ1
WNT3α
WNT5α
EGF
DMOG
LOXL2

## Slide 3
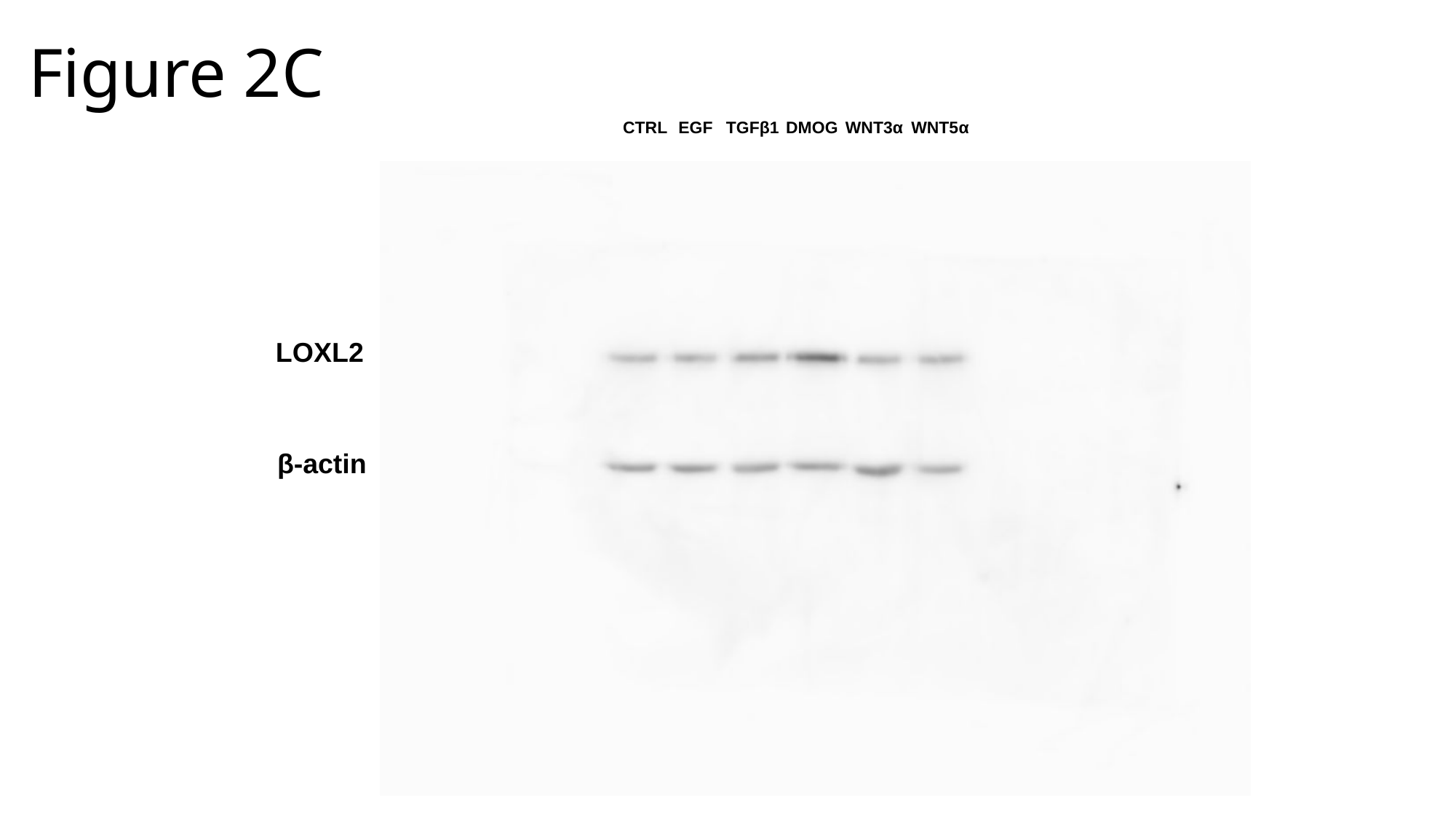

# Figure 2C
CTRL
TGFβ1
WNT3α
WNT5α
EGF
DMOG
LOXL2
β-actin
