# Supplementary figures and images for: Pseudohypoxic HIF pathway activation dysregulates collagen structure-function in human lung fibrosis
Source: eLife. 2022 Feb 21;11:e69348. doi: 10.7554/eLife.69348 (PMC8860444; doi:10.7554/eLife.69348)

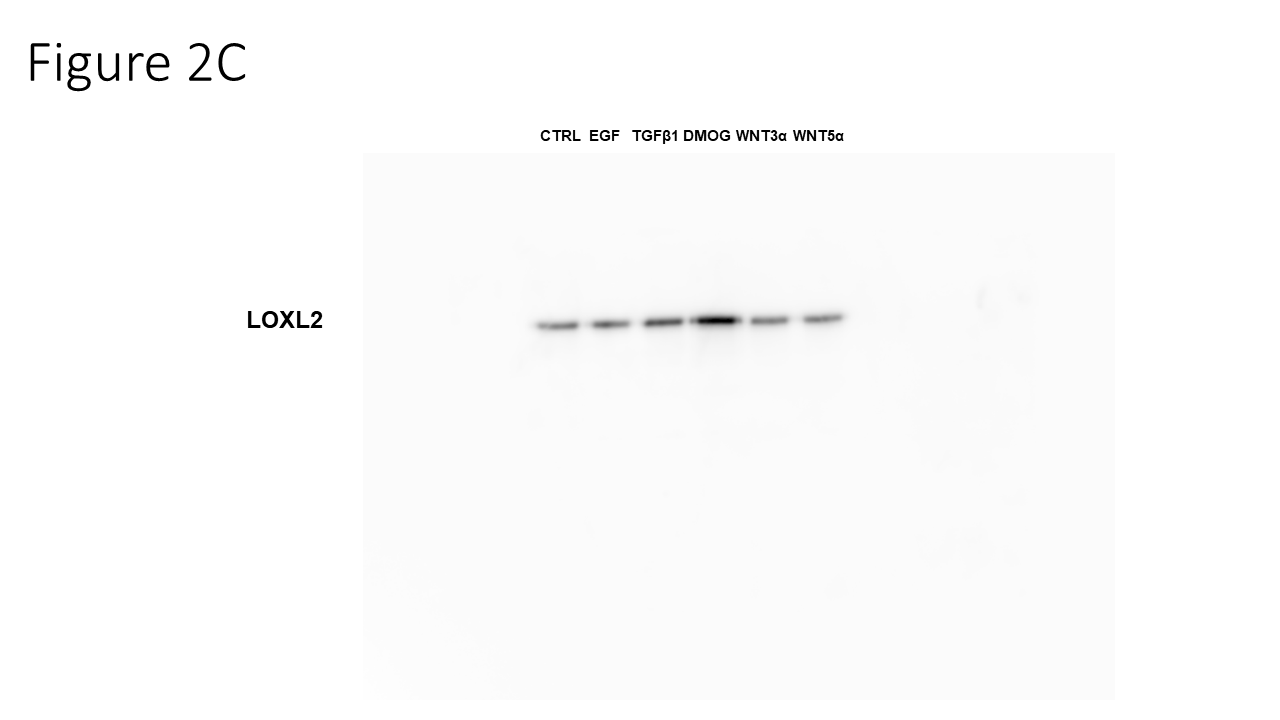

Supplement: Figure 2—source data 1. [file elife-69348-fig2-data1.zip › Figure 2C-source data 1/Labelled raw blot/LOXL2.tif]

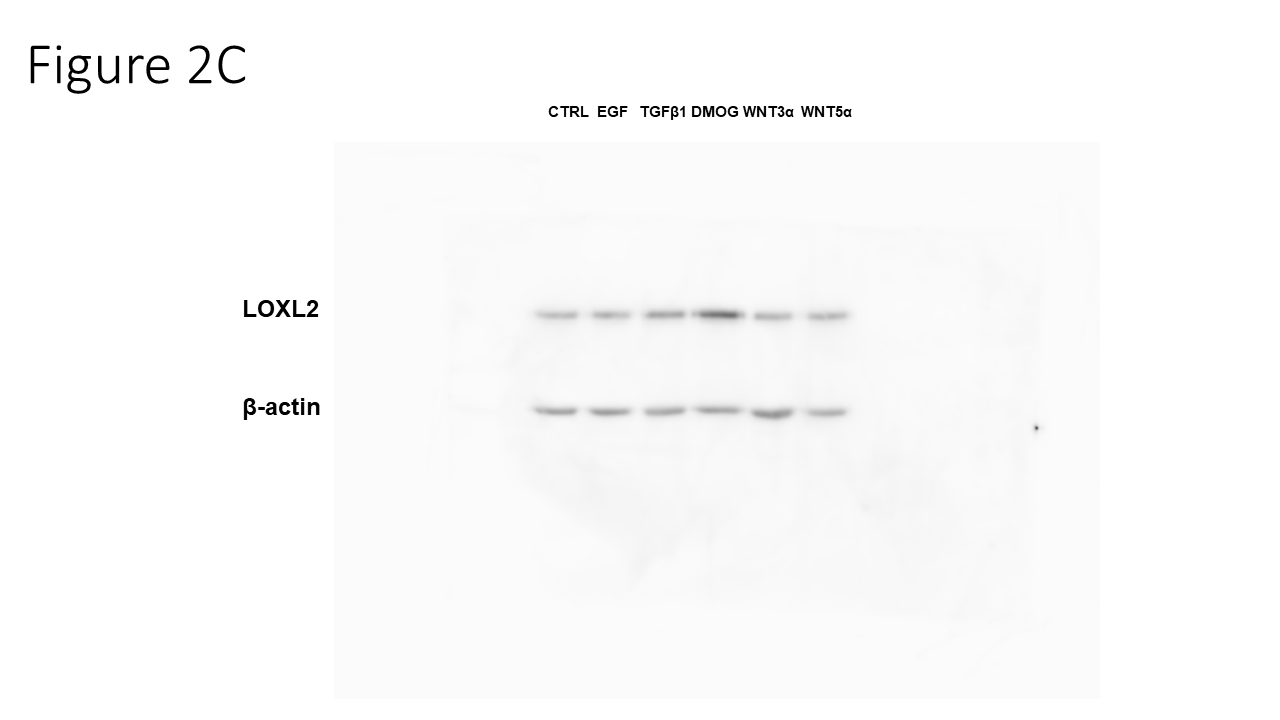

Supplement: Figure 2—source data 1. [file elife-69348-fig2-data1.zip › Figure 2C-source data 1/Labelled raw blot/B-actin.tif]

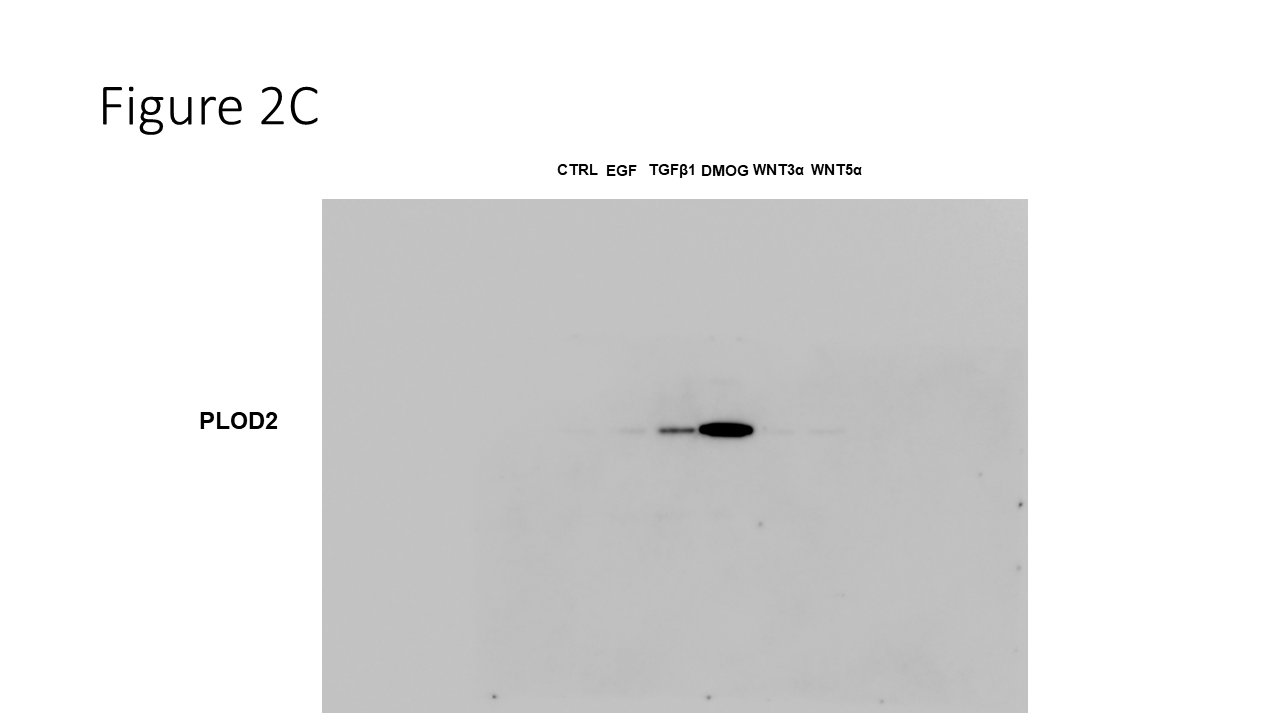

Supplement: Figure 2—source data 1. [file elife-69348-fig2-data1.zip › Figure 2C-source data 1/Labelled raw blot/PLOD2.tif]

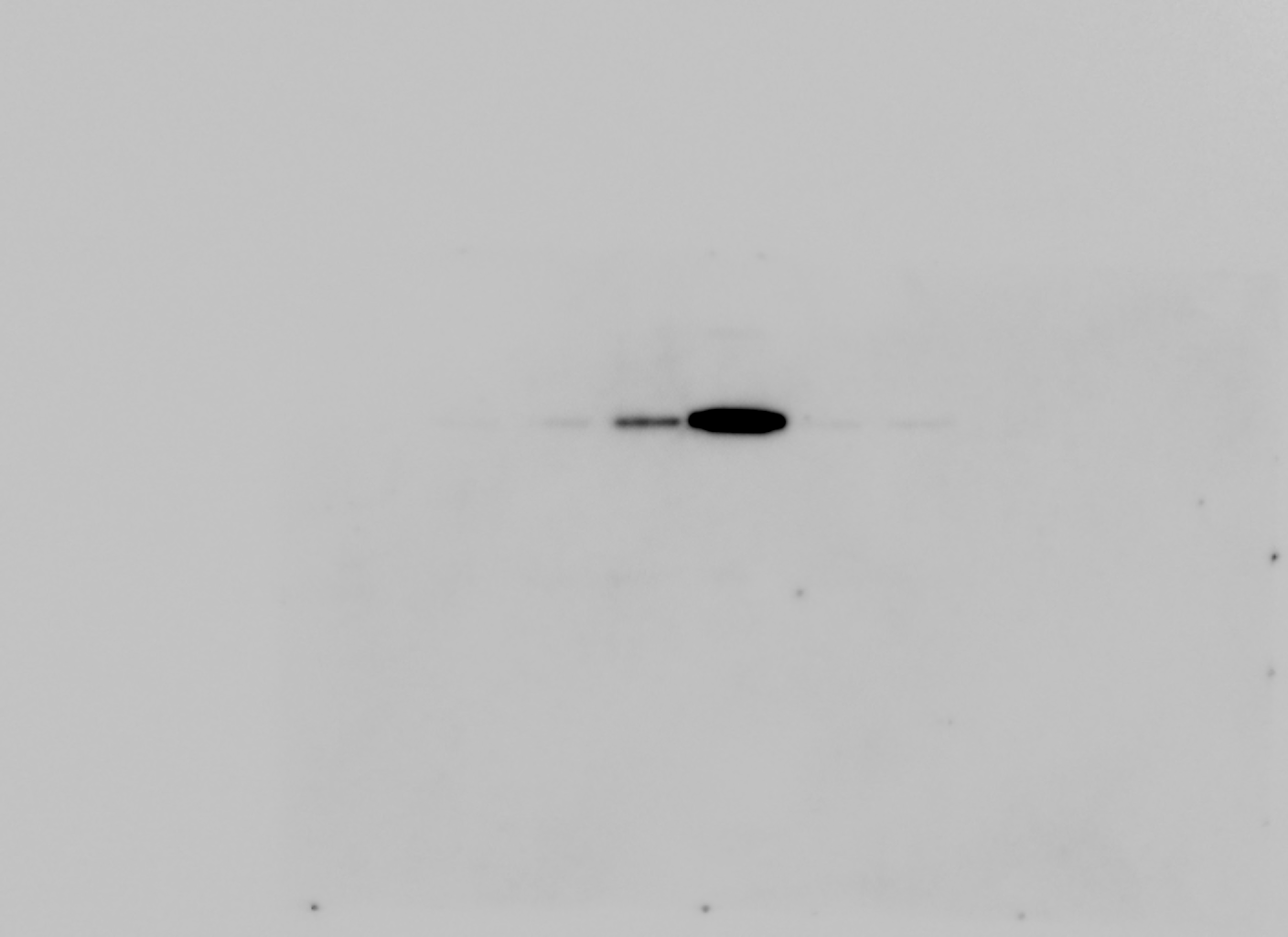

Supplement: Figure 2—source data 1. [file elife-69348-fig2-data1.zip › Figure 2C-source data 1/Raw blot/PLOD2 raw.tif]

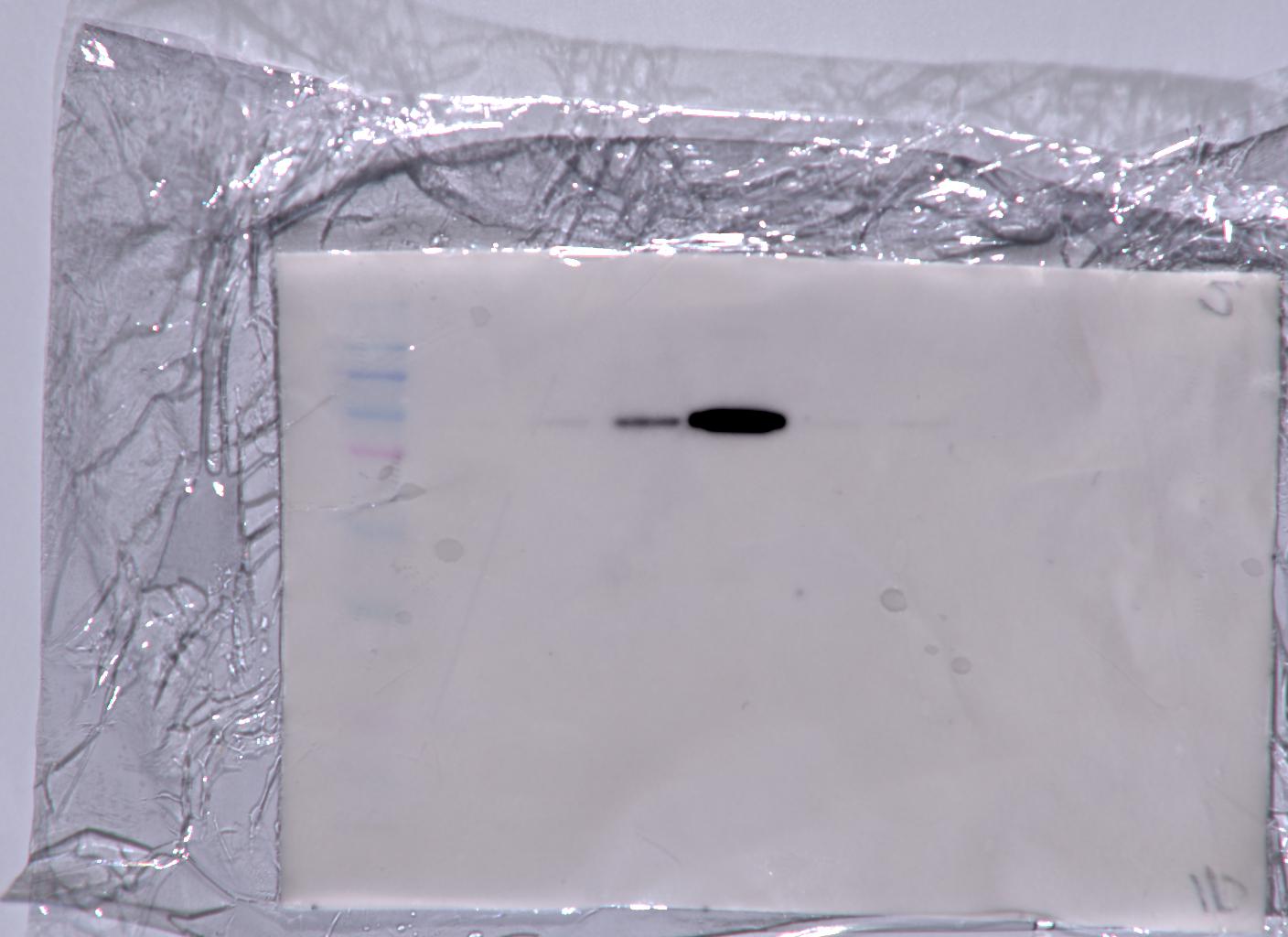

Supplement: Figure 2—source data 1. [file elife-69348-fig2-data1.zip › Figure 2C-source data 1/Raw blot/PLOD2 with MW ladder.tif]

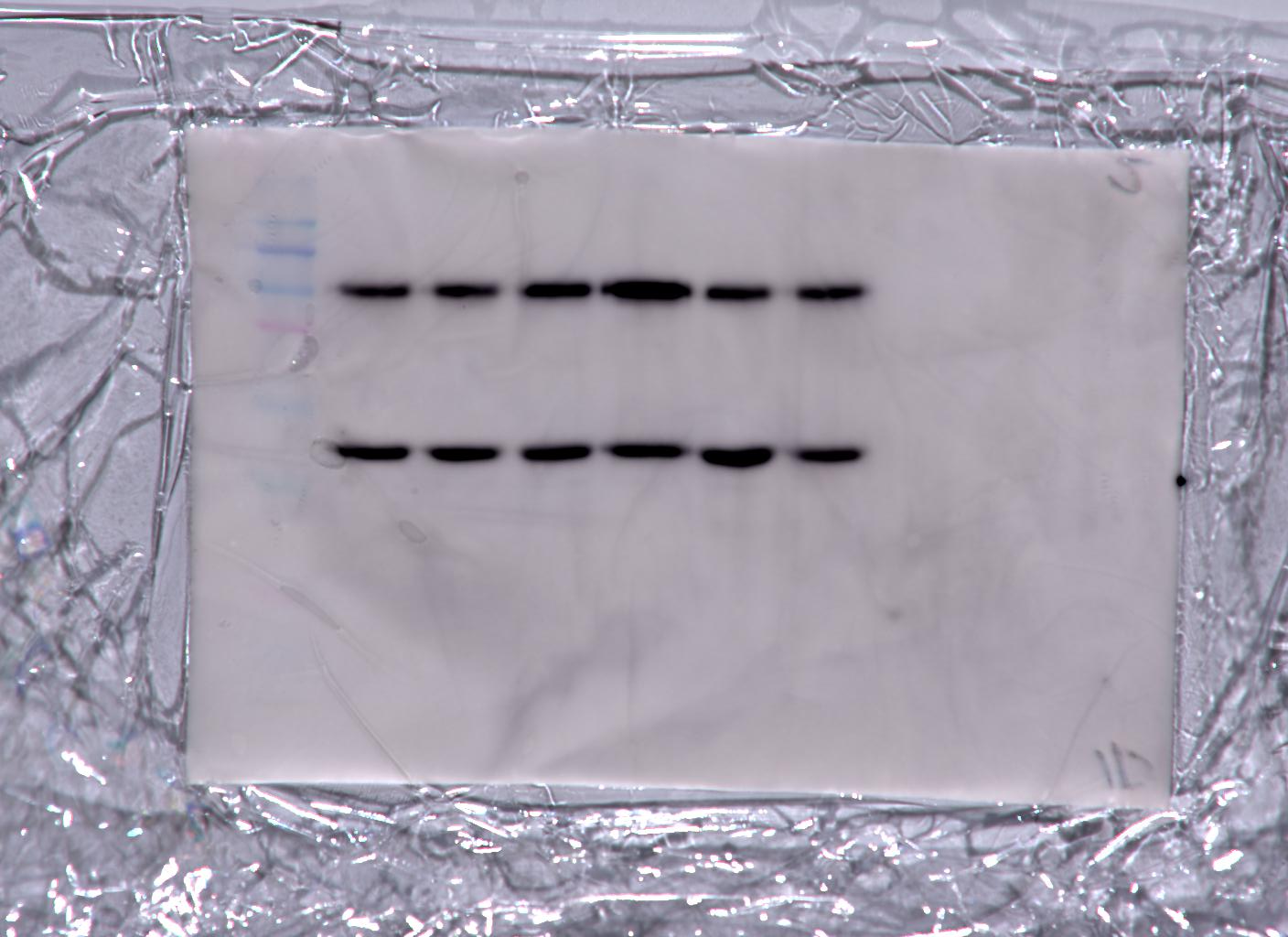

Supplement: Figure 2—source data 1. [file elife-69348-fig2-data1.zip › Figure 2C-source data 1/Raw blot/B-actin with MW ladder.tif]

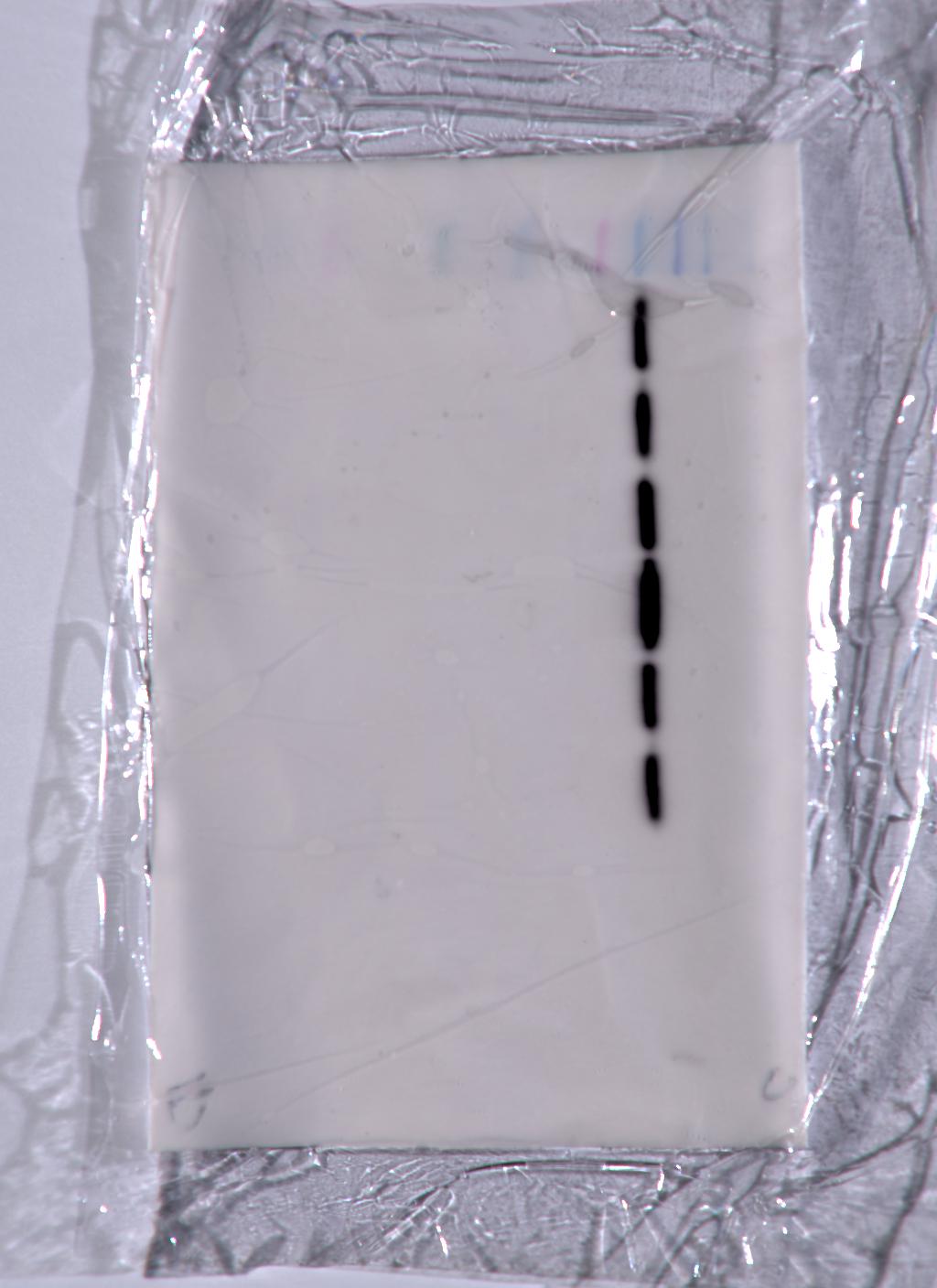

Supplement: Figure 2—source data 1. [file elife-69348-fig2-data1.zip › Figure 2C-source data 1/Raw blot/LOXL2 with MW ladder.tif]

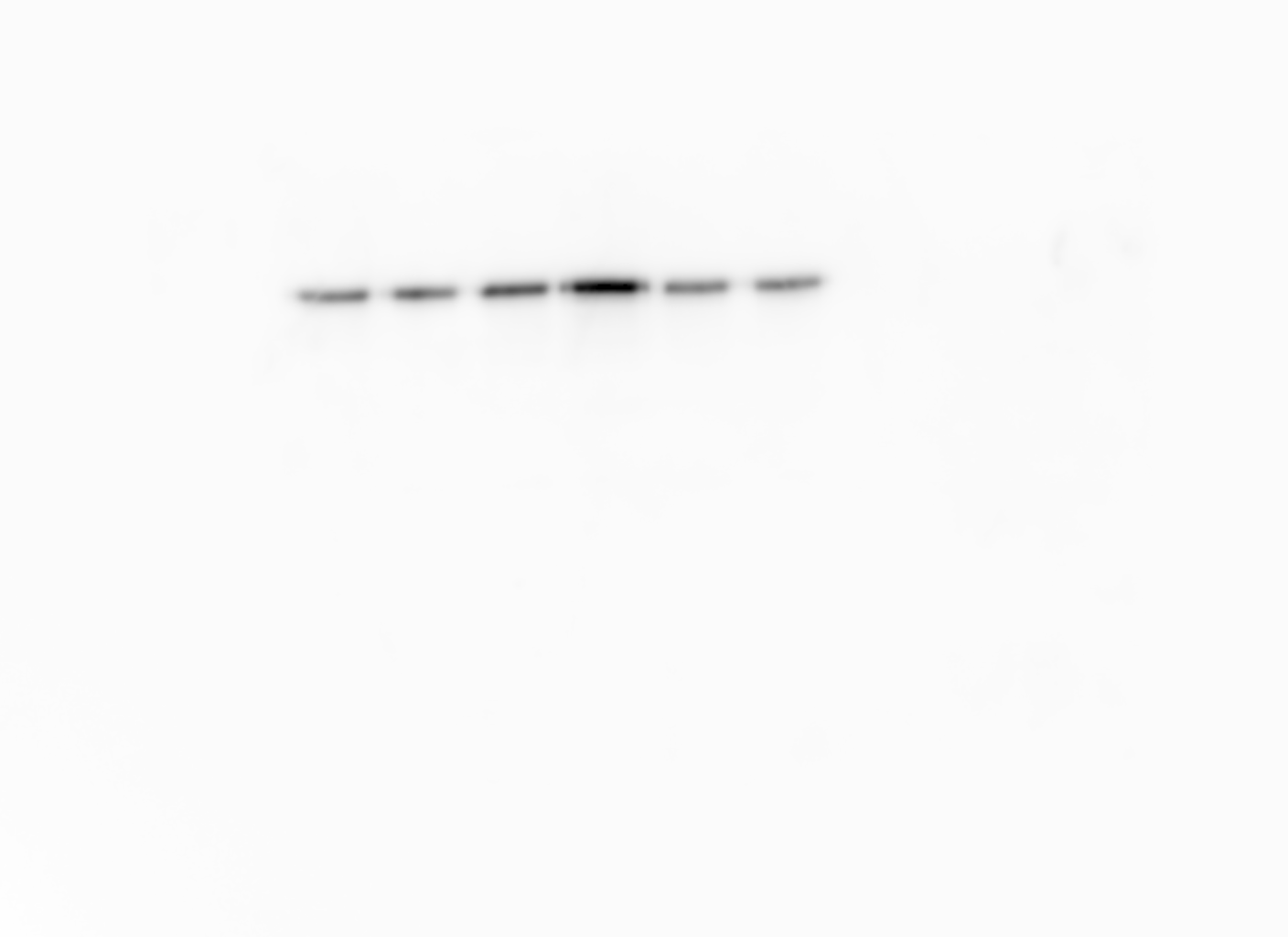

Supplement: Figure 2—source data 1. [file elife-69348-fig2-data1.zip › Figure 2C-source data 1/Raw blot/LOXL2 raw.tif]

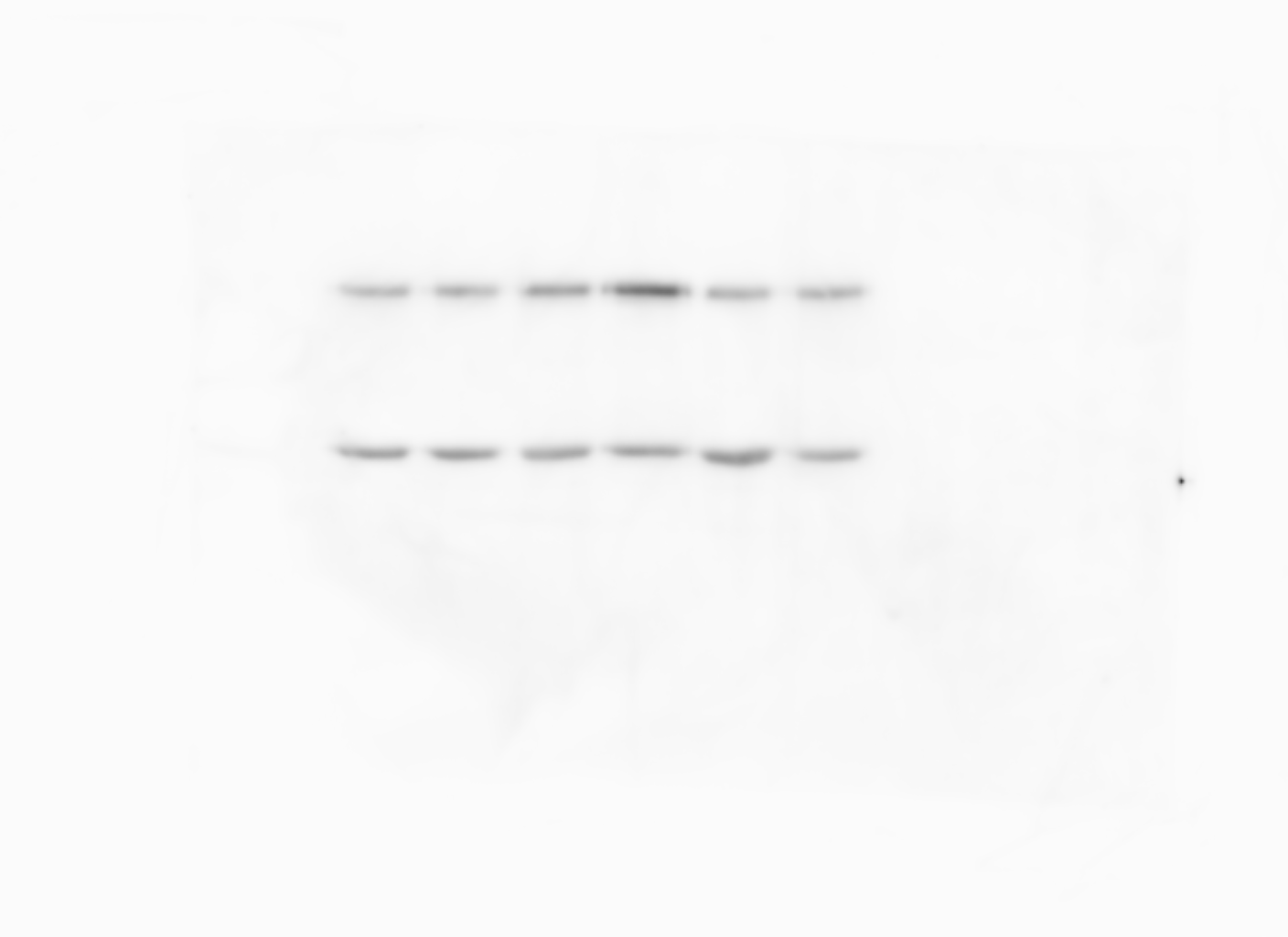

Supplement: Figure 2—source data 1. [file elife-69348-fig2-data1.zip › Figure 2C-source data 1/Raw blot/B-actin raw.tif]

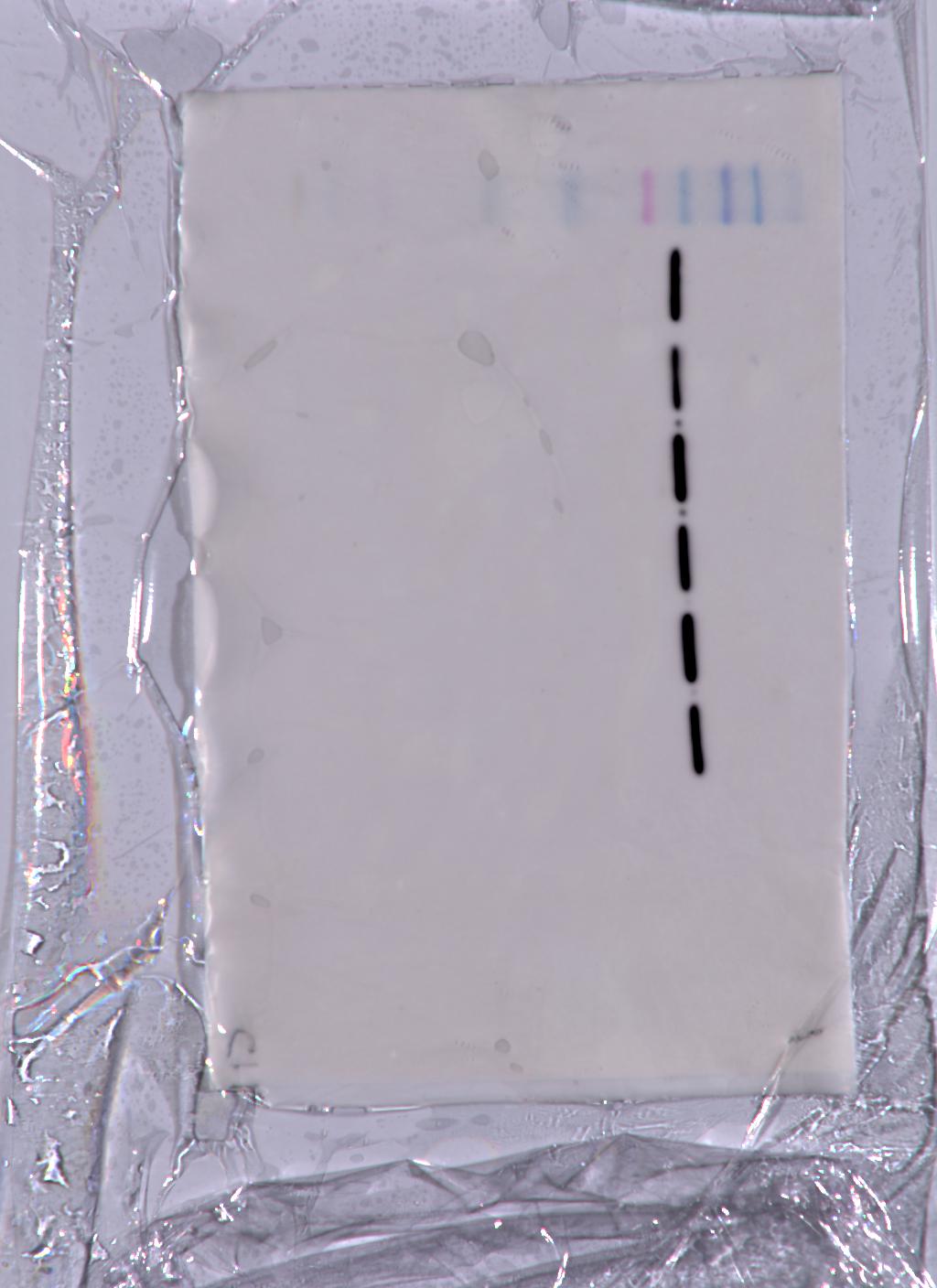

Supplement: Figure 2—figure supplement 1—source data 1. [file elife-69348-fig2-figsupp1-data1.zip › FIgure2-figure supplement 1-source data 1/FIgure2-figure supplement 1-source data 1a/Raw blot/Active B-catenin with MW ladder.tif]

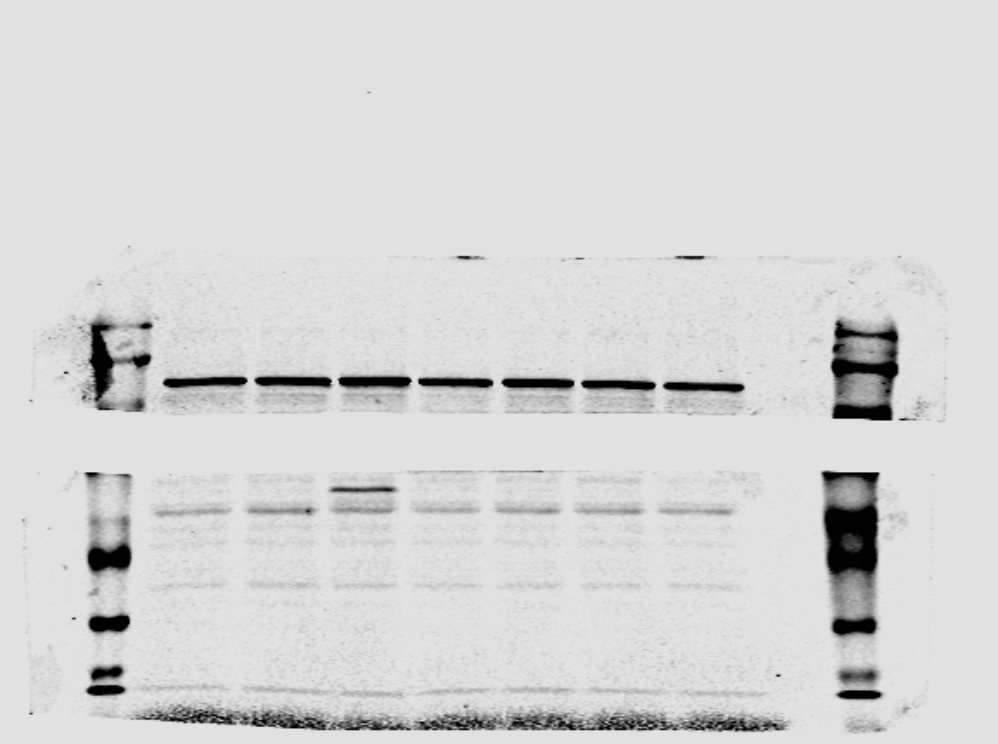

Supplement: Figure 2—figure supplement 1—source data 1. [file elife-69348-fig2-figsupp1-data1.zip › FIgure2-figure supplement 1-source data 1/FIgure2-figure supplement 1-source data 1a/Raw blot/P-smad raw.tif]

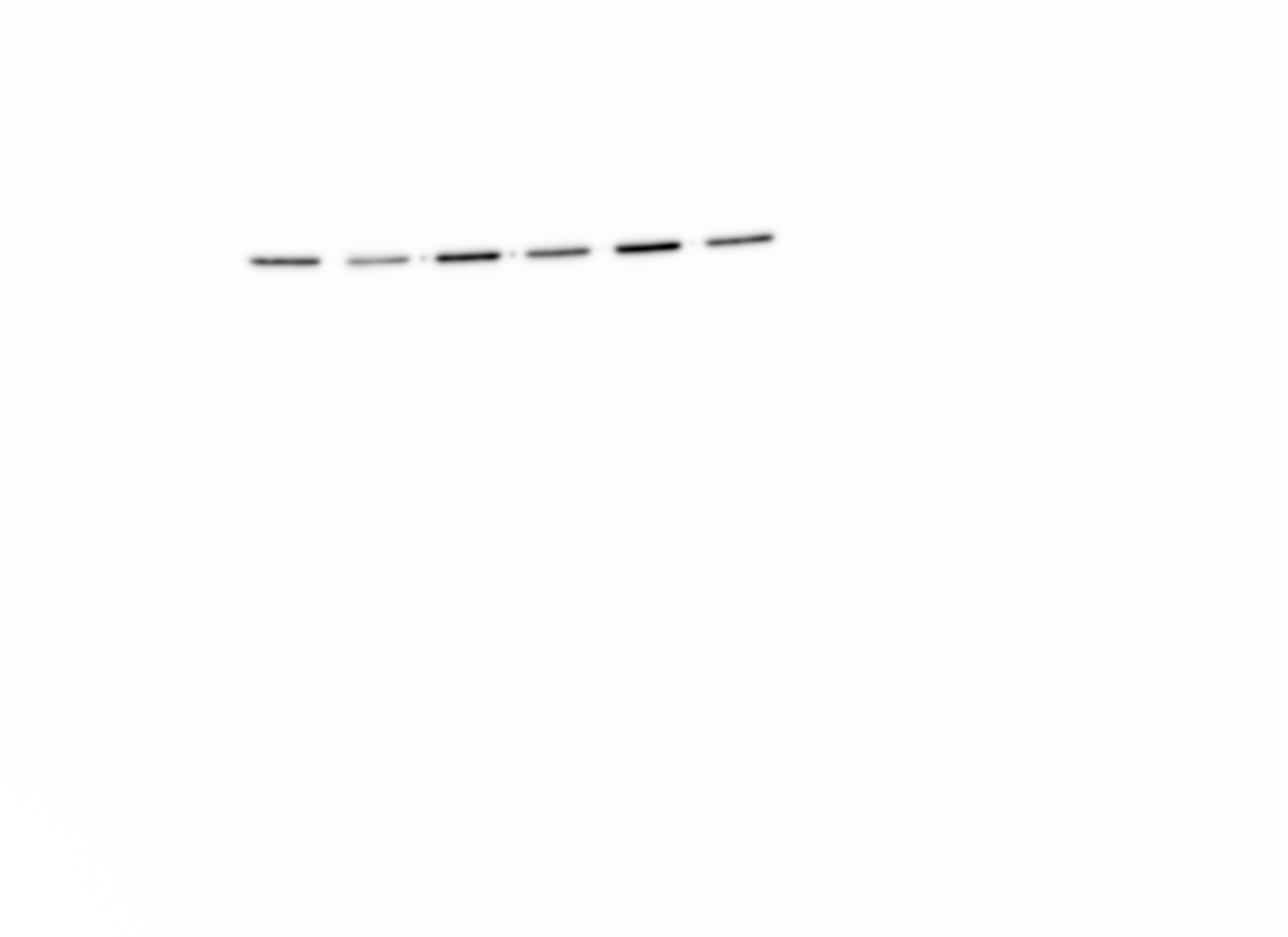

Supplement: Figure 2—figure supplement 1—source data 1. [file elife-69348-fig2-figsupp1-data1.zip › FIgure2-figure supplement 1-source data 1/FIgure2-figure supplement 1-source data 1a/Raw blot/Active B-catenin raw.tif]

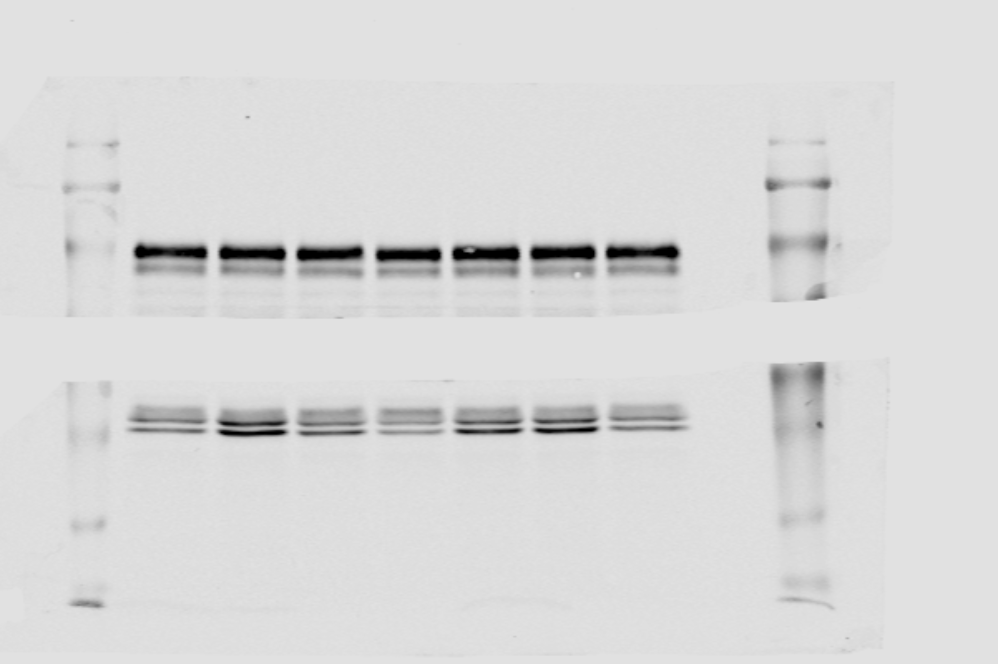

Supplement: Figure 2—figure supplement 1—source data 1. [file elife-69348-fig2-figsupp1-data1.zip › FIgure2-figure supplement 1-source data 1/FIgure2-figure supplement 1-source data 1a/Raw blot/P-Erk raw.tif]

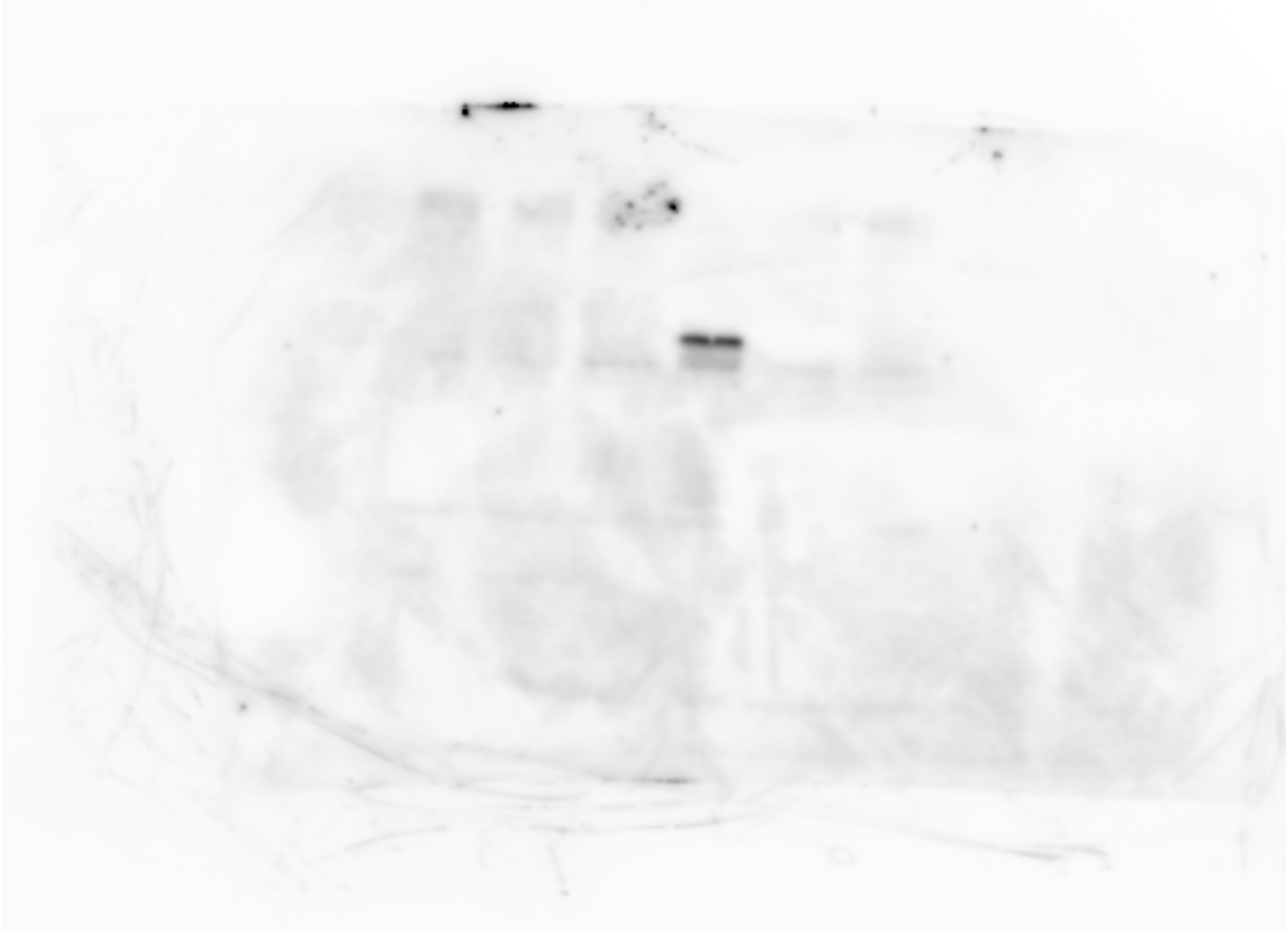

Supplement: Figure 2—figure supplement 1—source data 1. [file elife-69348-fig2-figsupp1-data1.zip › FIgure2-figure supplement 1-source data 1/FIgure2-figure supplement 1-source data 1a/Raw blot/HIF1A raw.tiff]

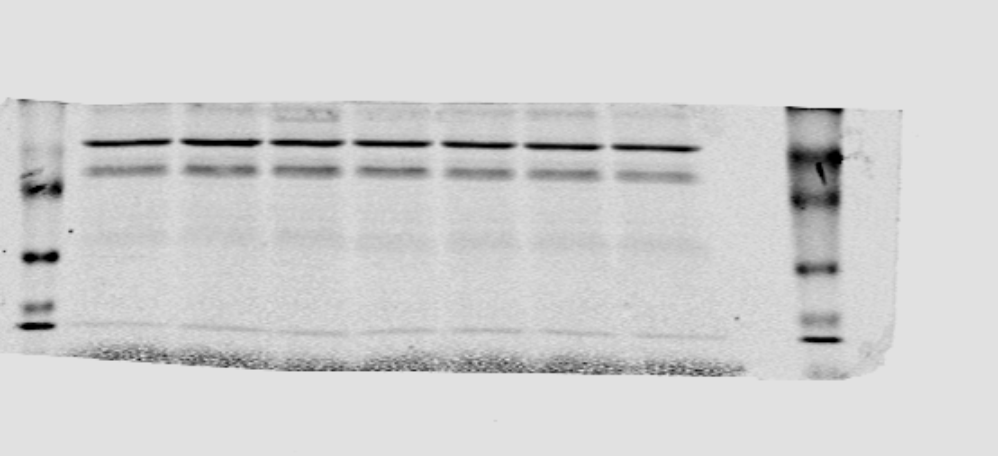

Supplement: Figure 2—figure supplement 1—source data 1. [file elife-69348-fig2-figsupp1-data1.zip › FIgure2-figure supplement 1-source data 1/FIgure2-figure supplement 1-source data 1a/Raw blot/B-tubulin raw.tif]

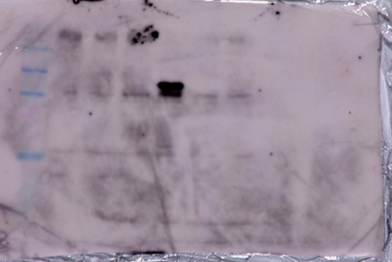

Supplement: Figure 2—figure supplement 1—source data 1. [file elife-69348-fig2-figsupp1-data1.zip › FIgure2-figure supplement 1-source data 1/FIgure2-figure supplement 1-source data 1a/Raw blot/HIF1A with MW ladder.tif]

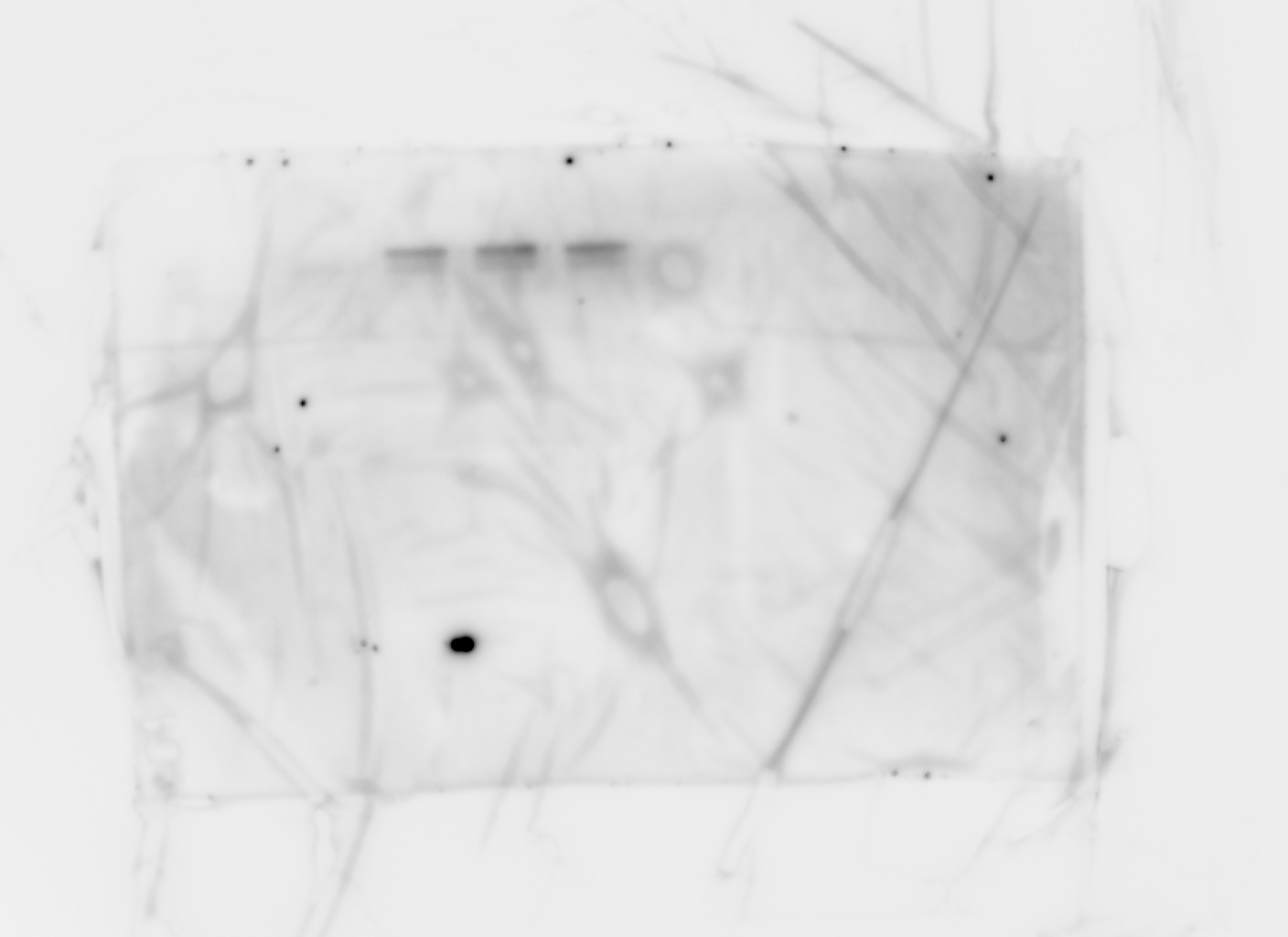

Supplement: Figure 2—figure supplement 1—source data 1. [file elife-69348-fig2-figsupp1-data1.zip › FIgure2-figure supplement 1-source data 1/FIgure2-figure supplement 1-source data 1d/Raw blot/HIF1A raw.tif]

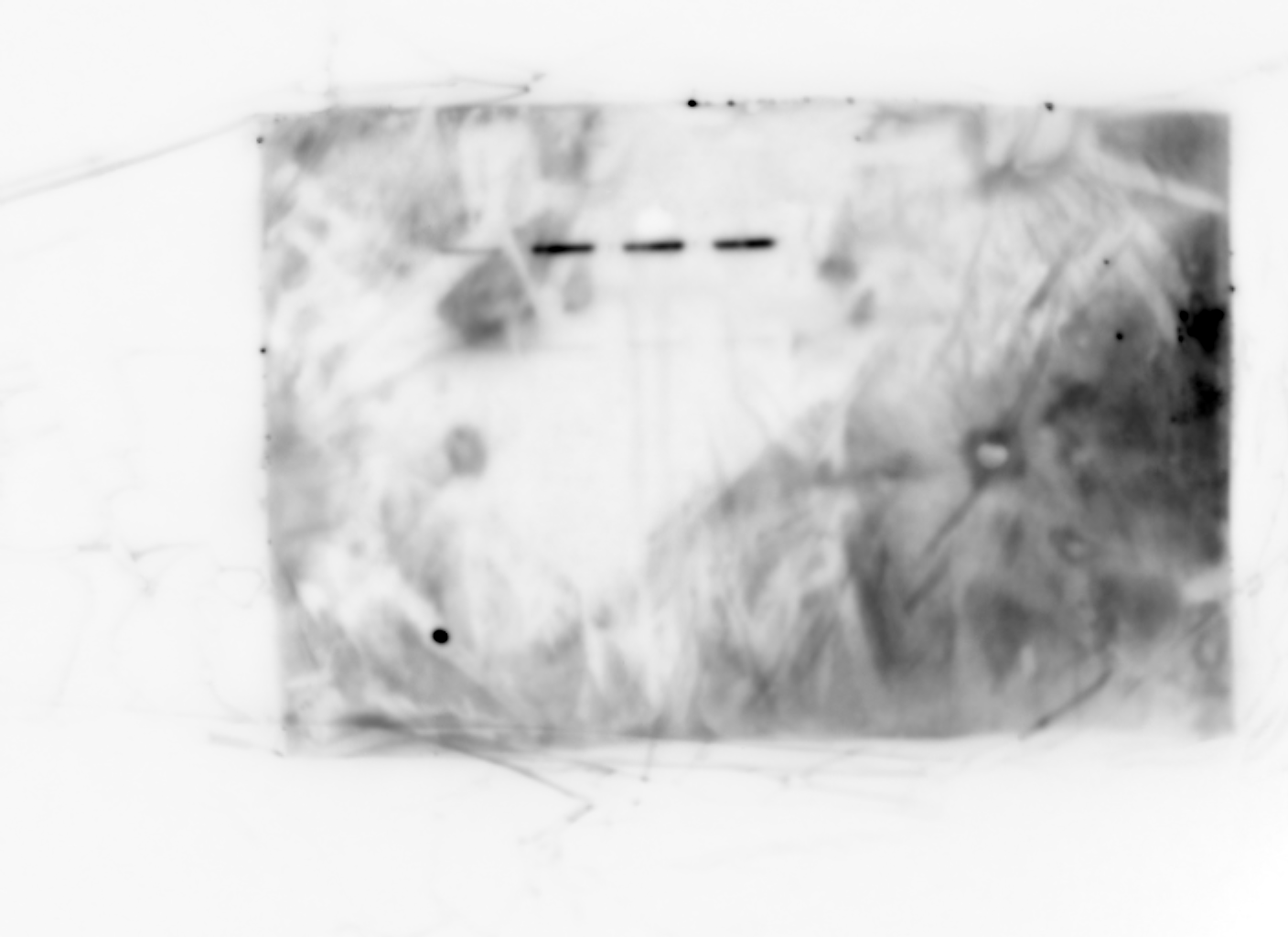

Supplement: Figure 2—figure supplement 1—source data 1. [file elife-69348-fig2-figsupp1-data1.zip › FIgure2-figure supplement 1-source data 1/FIgure2-figure supplement 1-source data 1d/Raw blot/PLOD2 raw.tif]

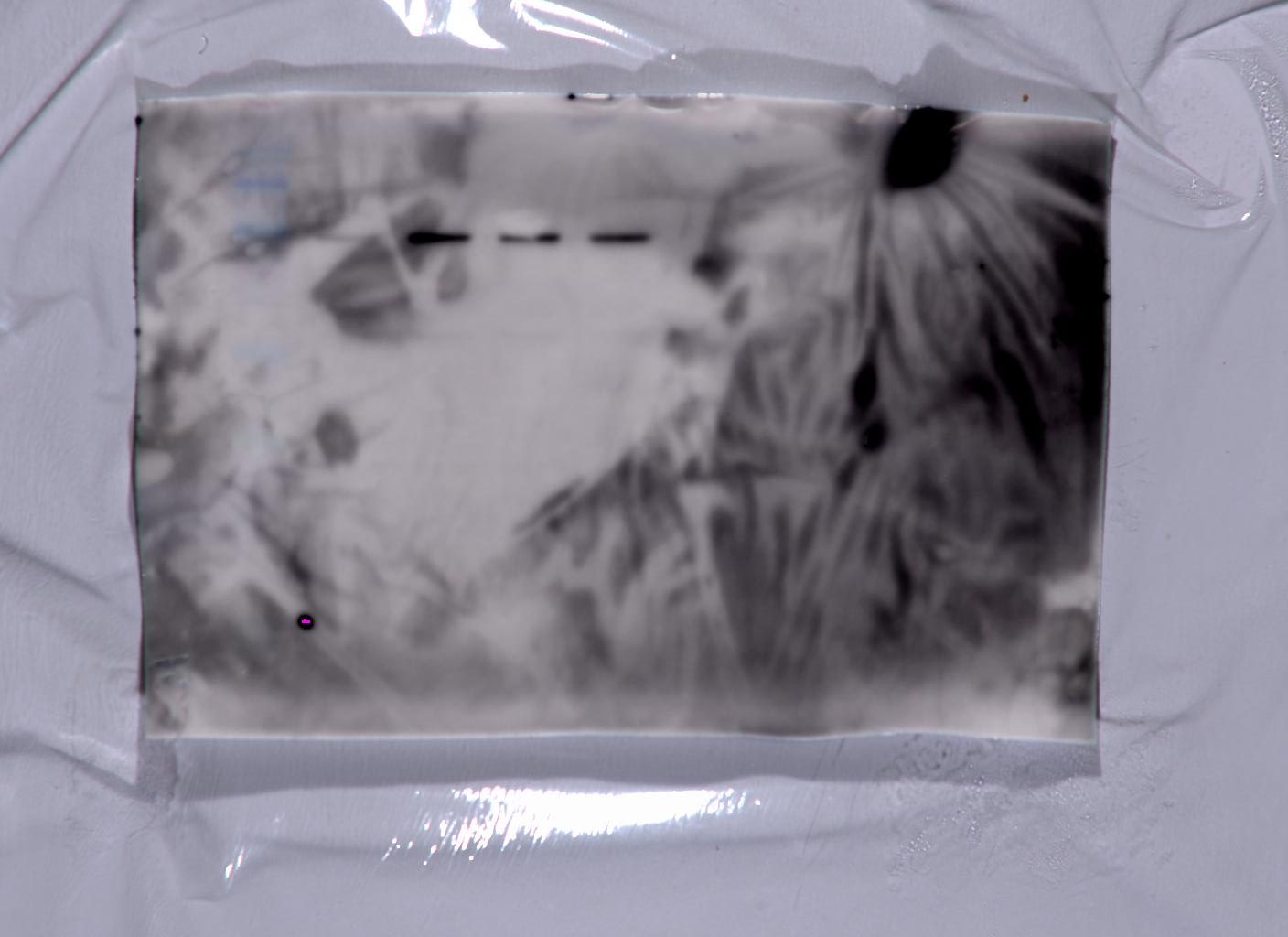

Supplement: Figure 2—figure supplement 1—source data 1. [file elife-69348-fig2-figsupp1-data1.zip › FIgure2-figure supplement 1-source data 1/FIgure2-figure supplement 1-source data 1d/Raw blot/PLOD2 with MW ladder.tif]

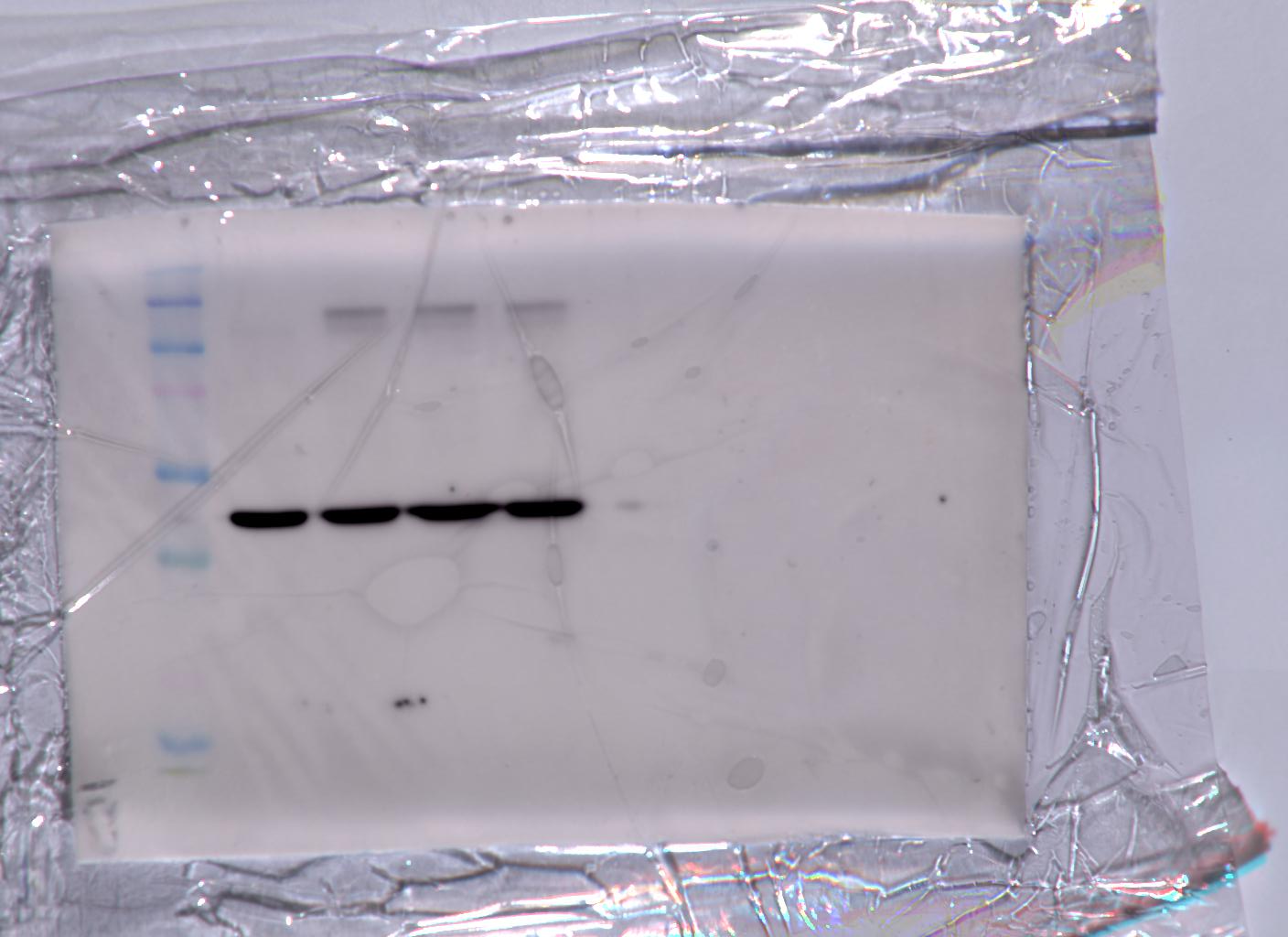

Supplement: Figure 2—figure supplement 1—source data 1. [file elife-69348-fig2-figsupp1-data1.zip › FIgure2-figure supplement 1-source data 1/FIgure2-figure supplement 1-source data 1d/Raw blot/B-actin with MW ladder.tif]

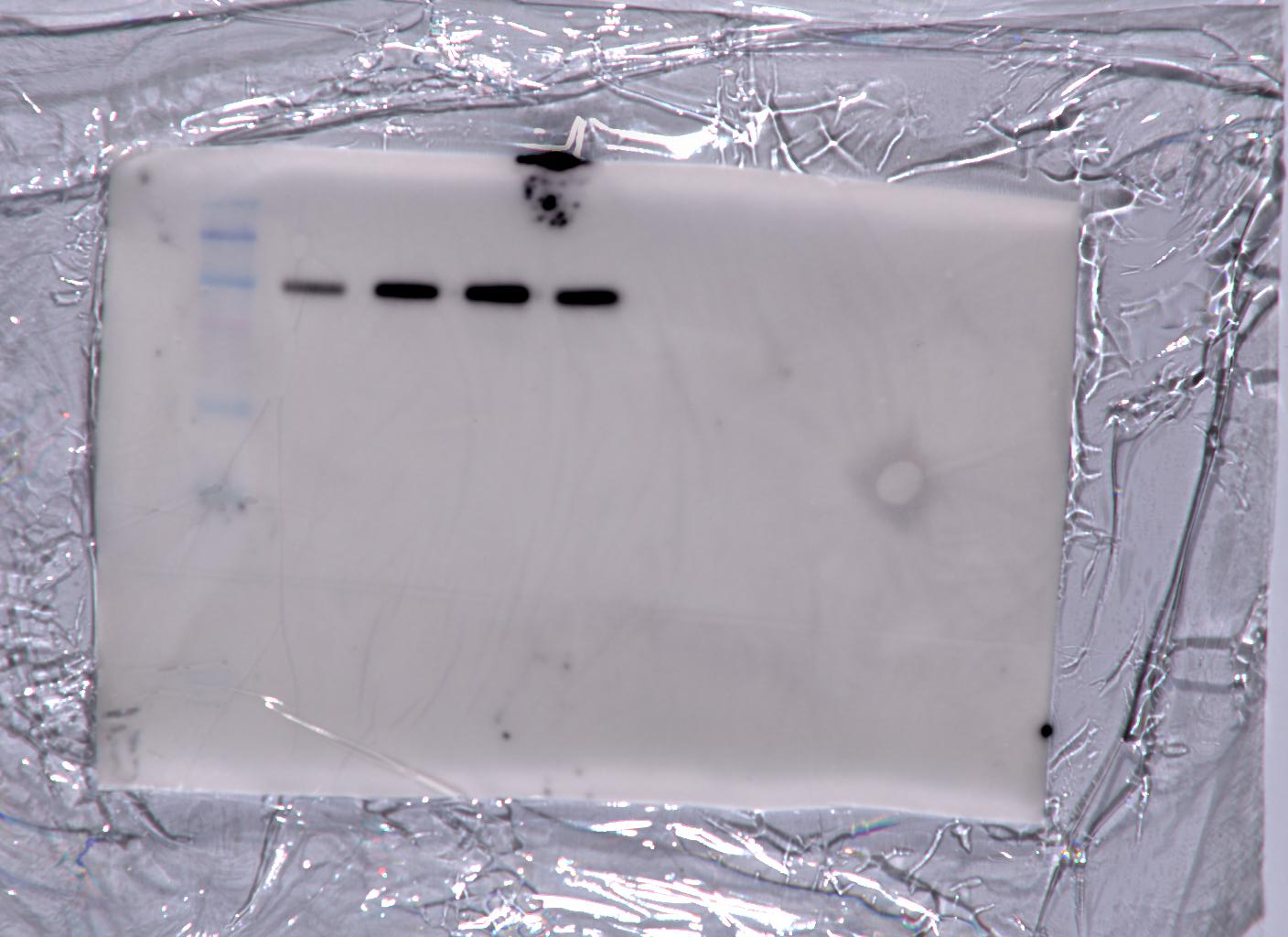

Supplement: Figure 2—figure supplement 1—source data 1. [file elife-69348-fig2-figsupp1-data1.zip › FIgure2-figure supplement 1-source data 1/FIgure2-figure supplement 1-source data 1d/Raw blot/LOXL2 with MW ladder.tif]

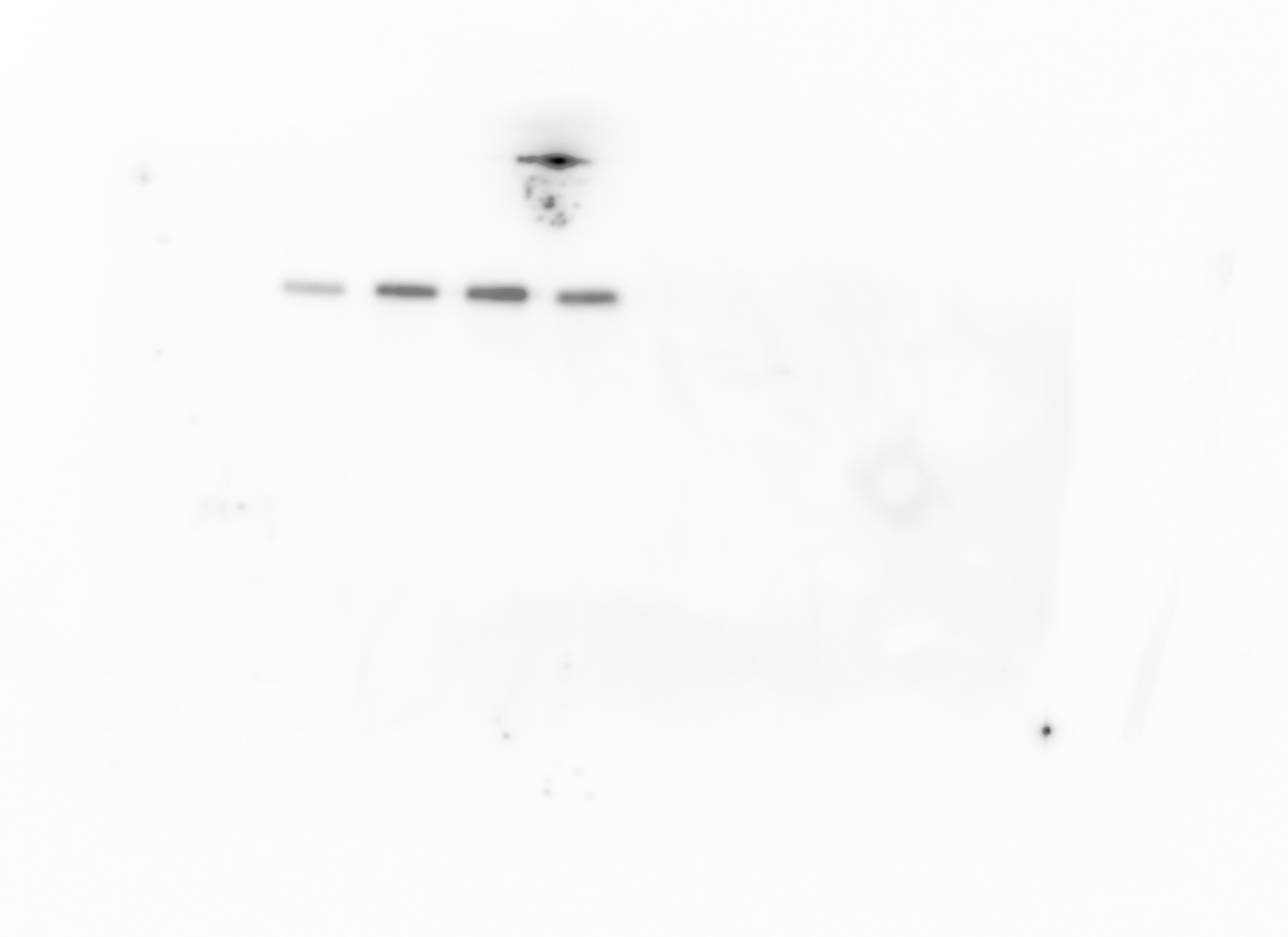

Supplement: Figure 2—figure supplement 1—source data 1. [file elife-69348-fig2-figsupp1-data1.zip › FIgure2-figure supplement 1-source data 1/FIgure2-figure supplement 1-source data 1d/Raw blot/LOXL2 raw.tif]

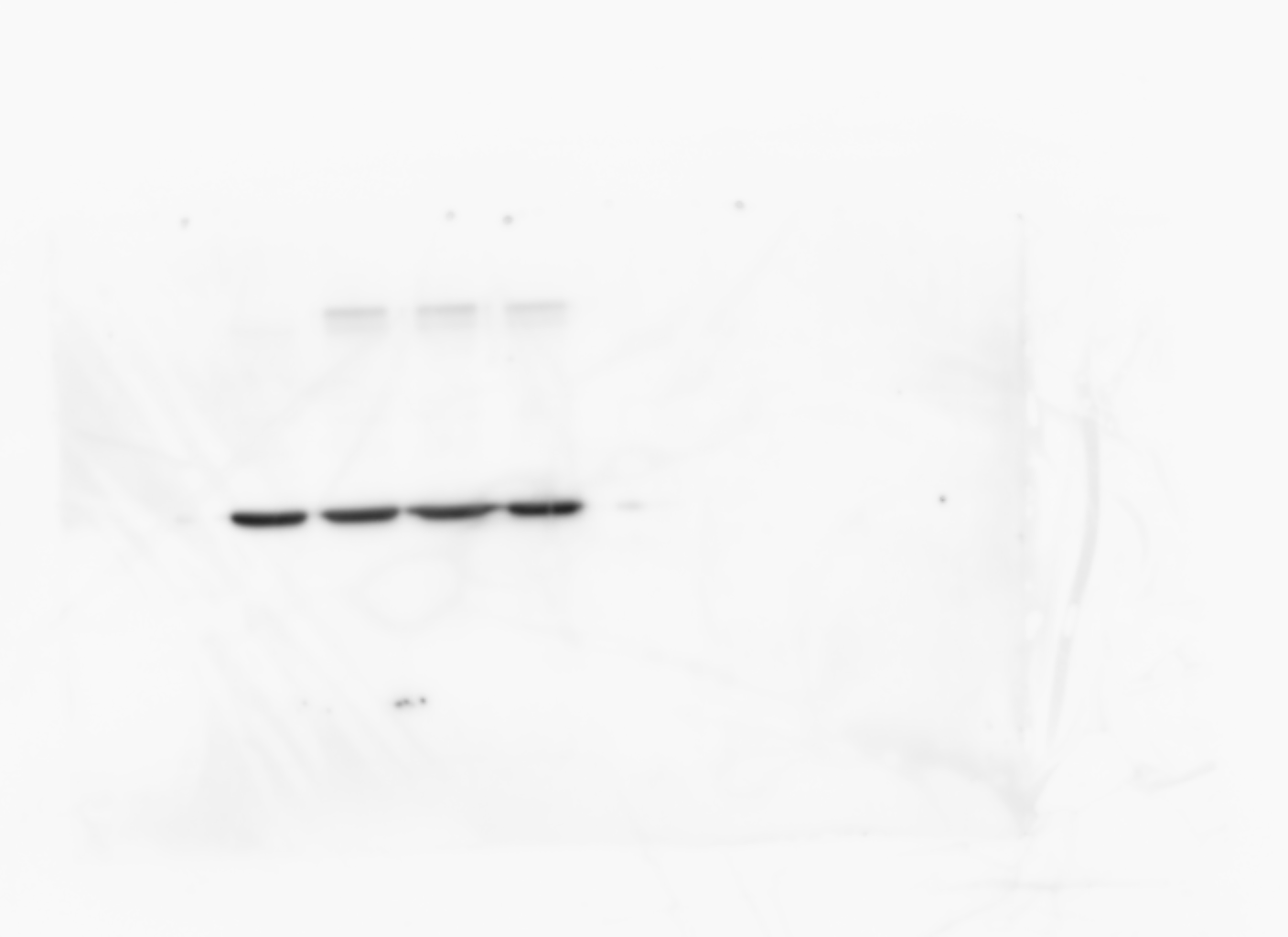

Supplement: Figure 2—figure supplement 1—source data 1. [file elife-69348-fig2-figsupp1-data1.zip › FIgure2-figure supplement 1-source data 1/FIgure2-figure supplement 1-source data 1d/Raw blot/B-actin raw.tif]

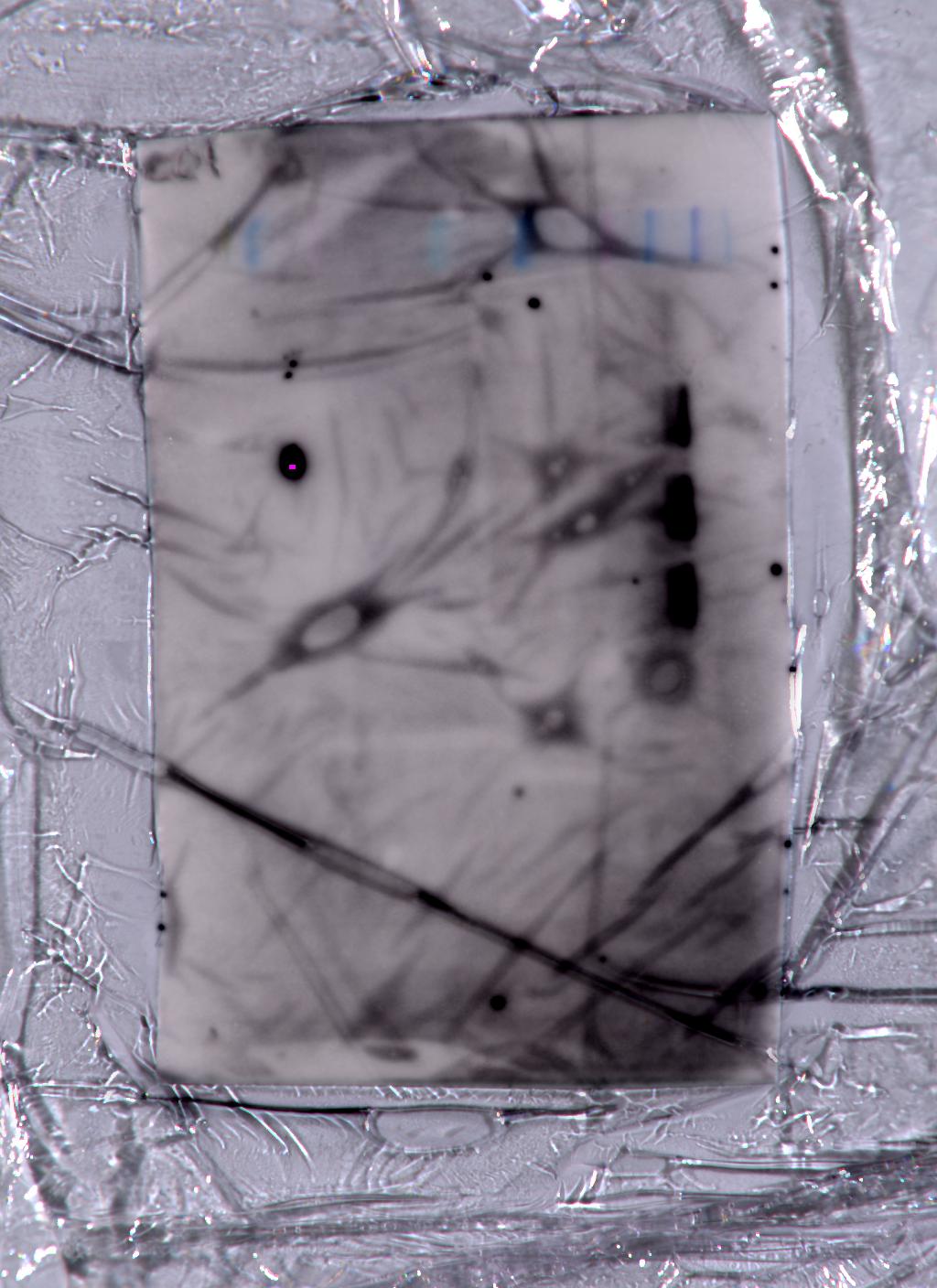

Supplement: Figure 2—figure supplement 1—source data 1. [file elife-69348-fig2-figsupp1-data1.zip › FIgure2-figure supplement 1-source data 1/FIgure2-figure supplement 1-source data 1d/Raw blot/HIF1A with MW ladder.tif]

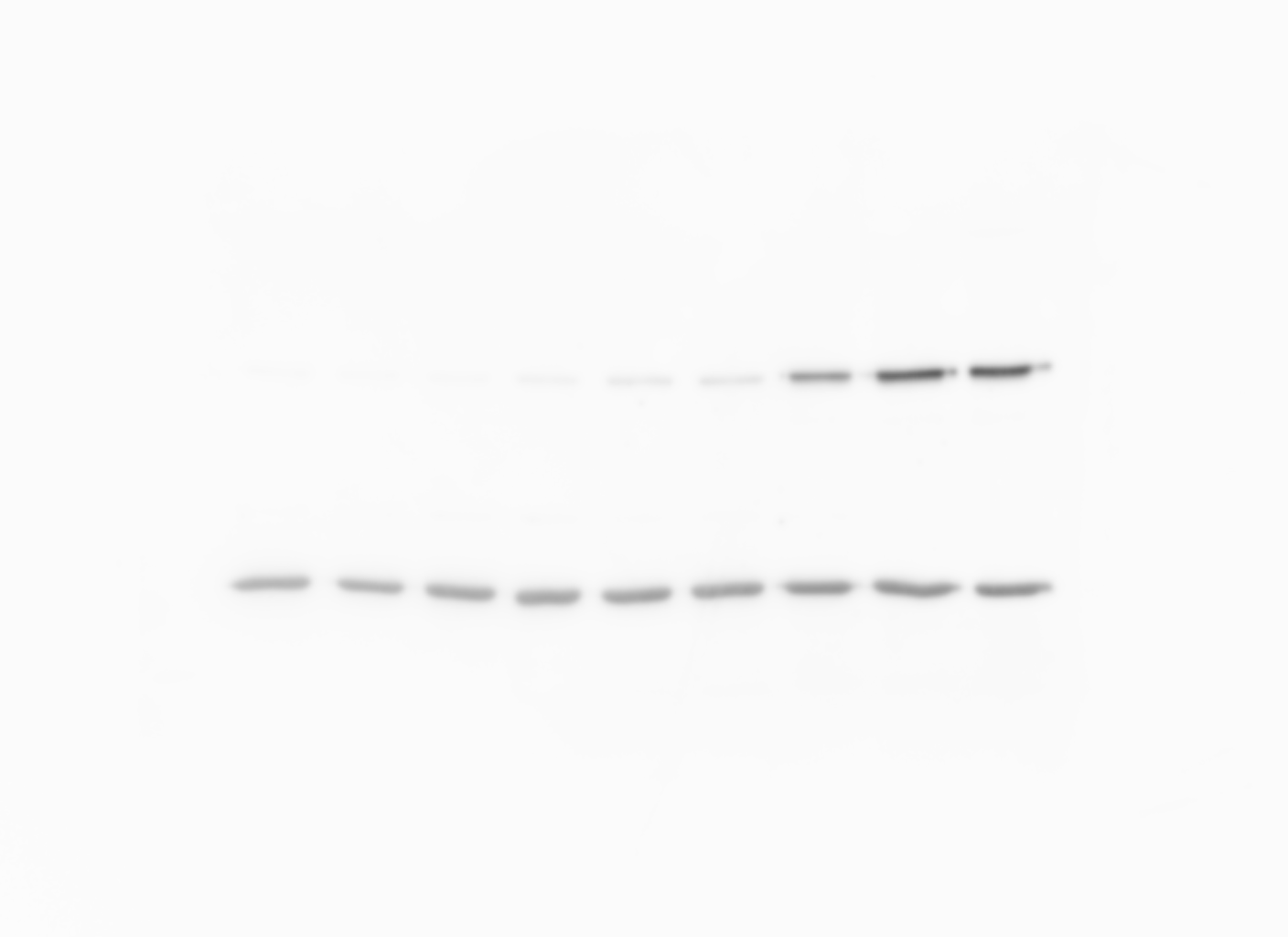

Supplement: Figure 2—figure supplement 1—source data 1. [file elife-69348-fig2-figsupp1-data1.zip › FIgure2-figure supplement 1-source data 1/FIgure2-figure supplement 1-source data 1b/Raw blot/PLOD2 raw.tif]

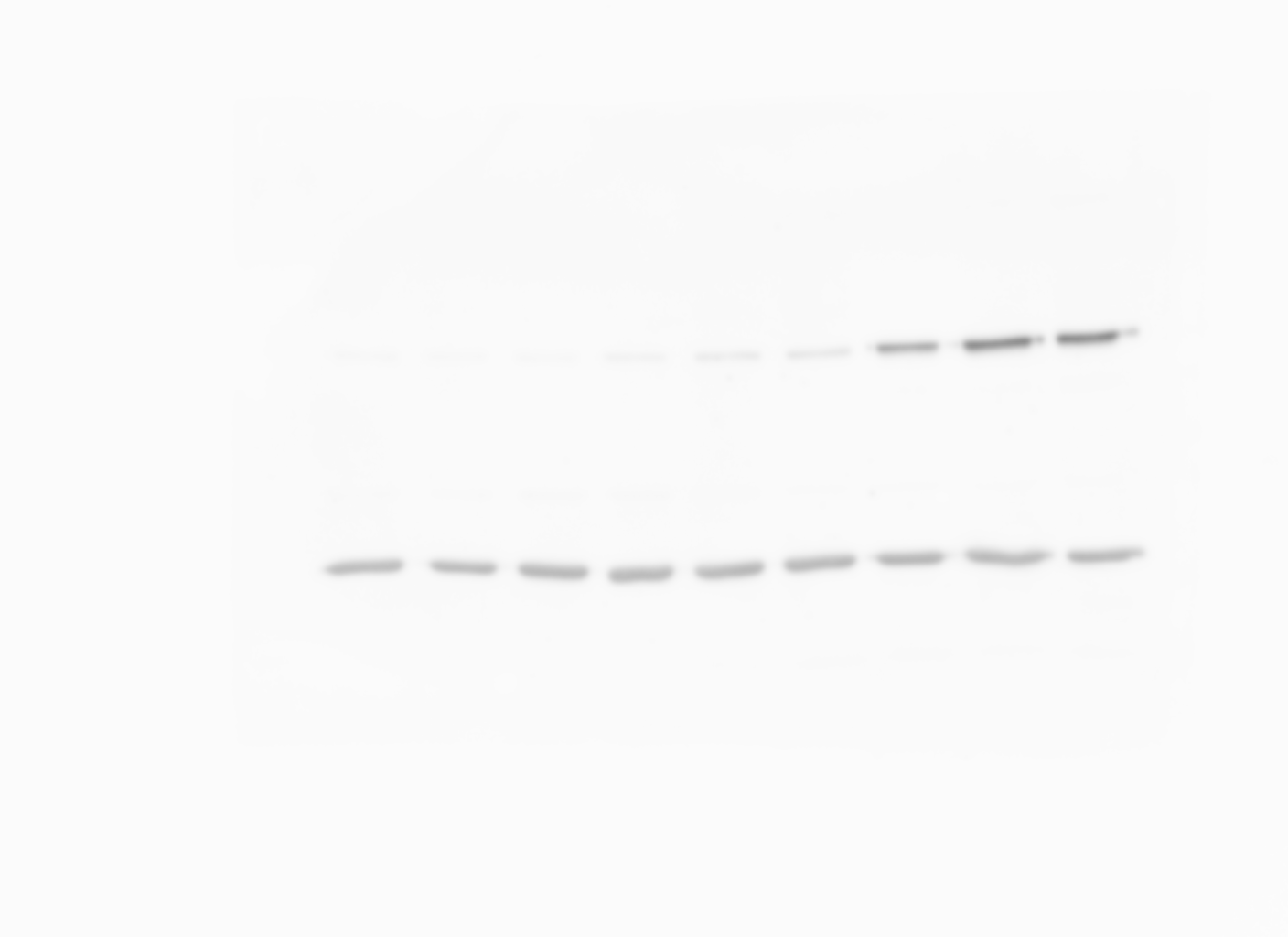

Supplement: Figure 2—figure supplement 1—source data 1. [file elife-69348-fig2-figsupp1-data1.zip › FIgure2-figure supplement 1-source data 1/FIgure2-figure supplement 1-source data 1b/Raw blot/B-actin for PLOD2 raw.tif]

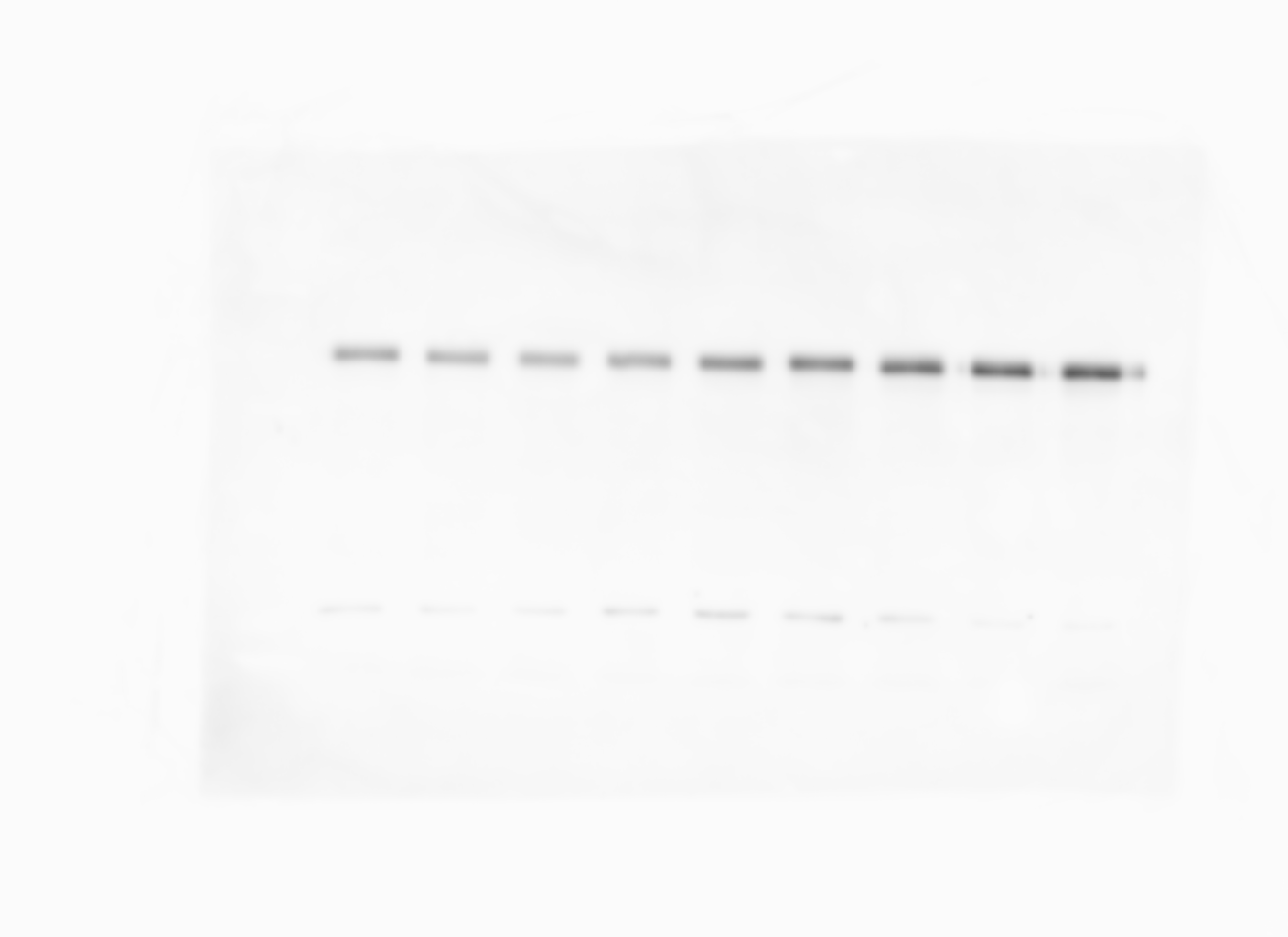

Supplement: Figure 2—figure supplement 1—source data 1. [file elife-69348-fig2-figsupp1-data1.zip › FIgure2-figure supplement 1-source data 1/FIgure2-figure supplement 1-source data 1b/Raw blot/LOXL2 raw.tif]

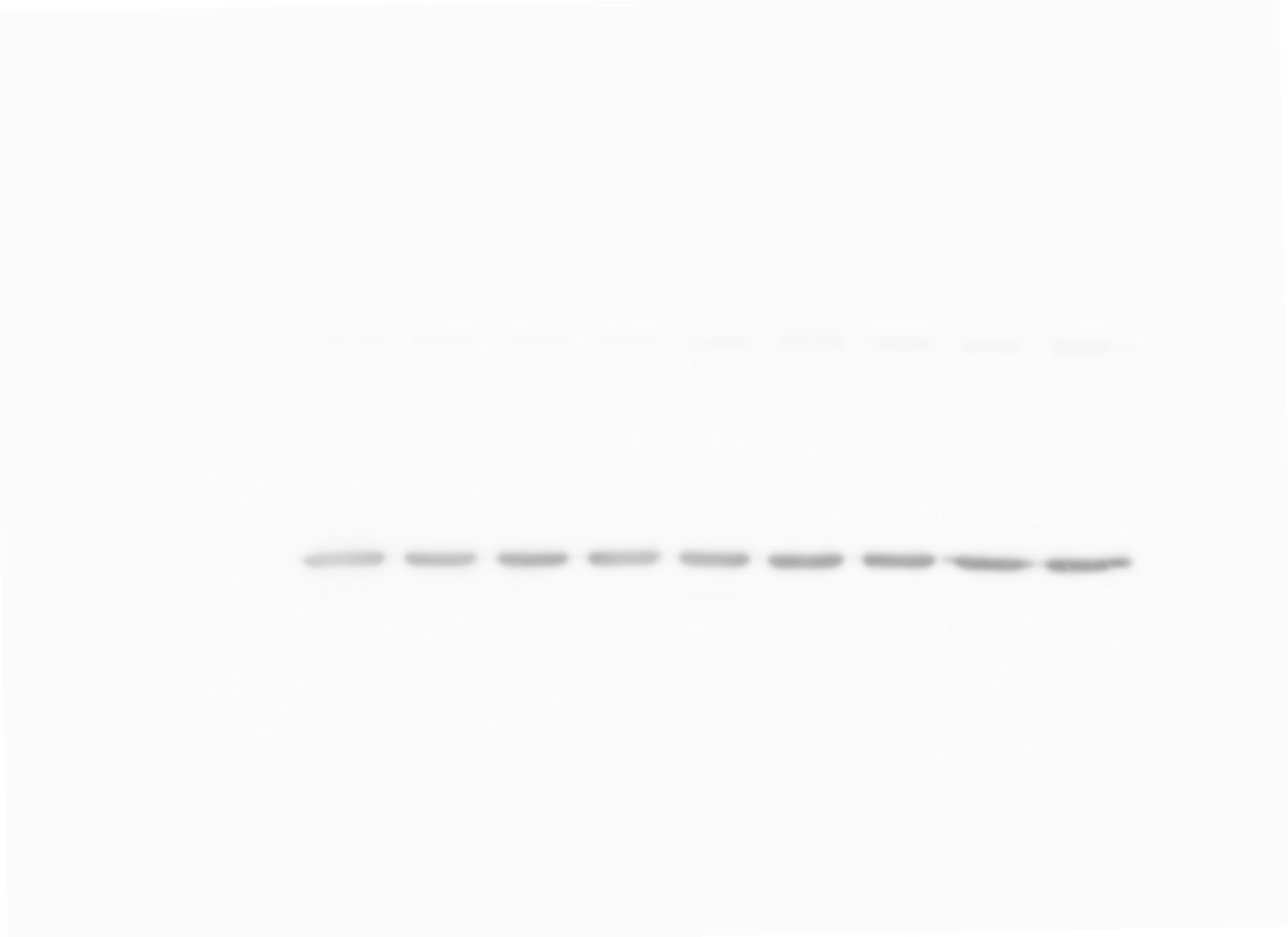

Supplement: Figure 2—figure supplement 1—source data 1. [file elife-69348-fig2-figsupp1-data1.zip › FIgure2-figure supplement 1-source data 1/FIgure2-figure supplement 1-source data 1b/Raw blot/B-actin for LOXL2 raw.tif]

## Slide 1
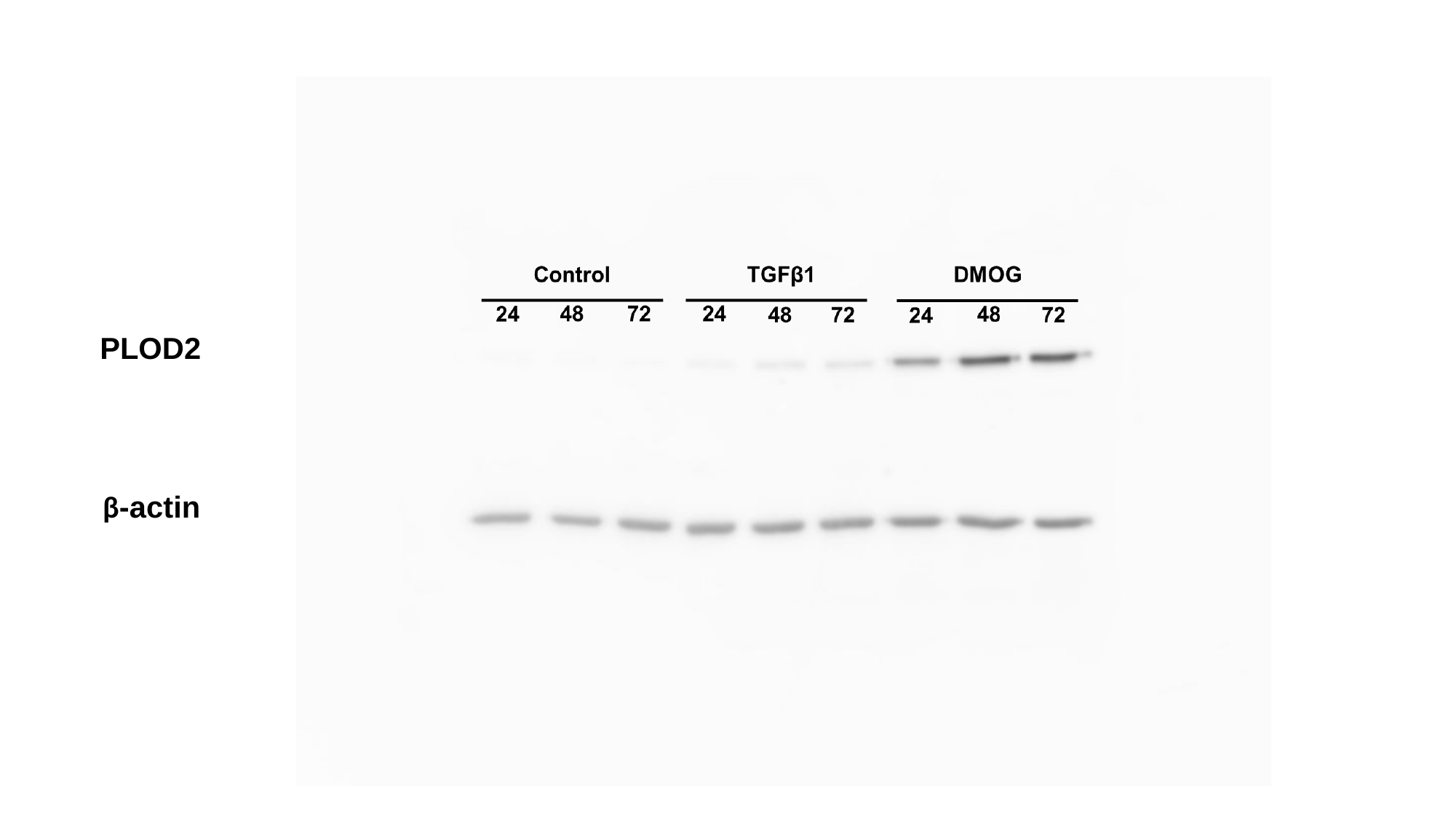

PLOD2
β-actin

## Slide 2
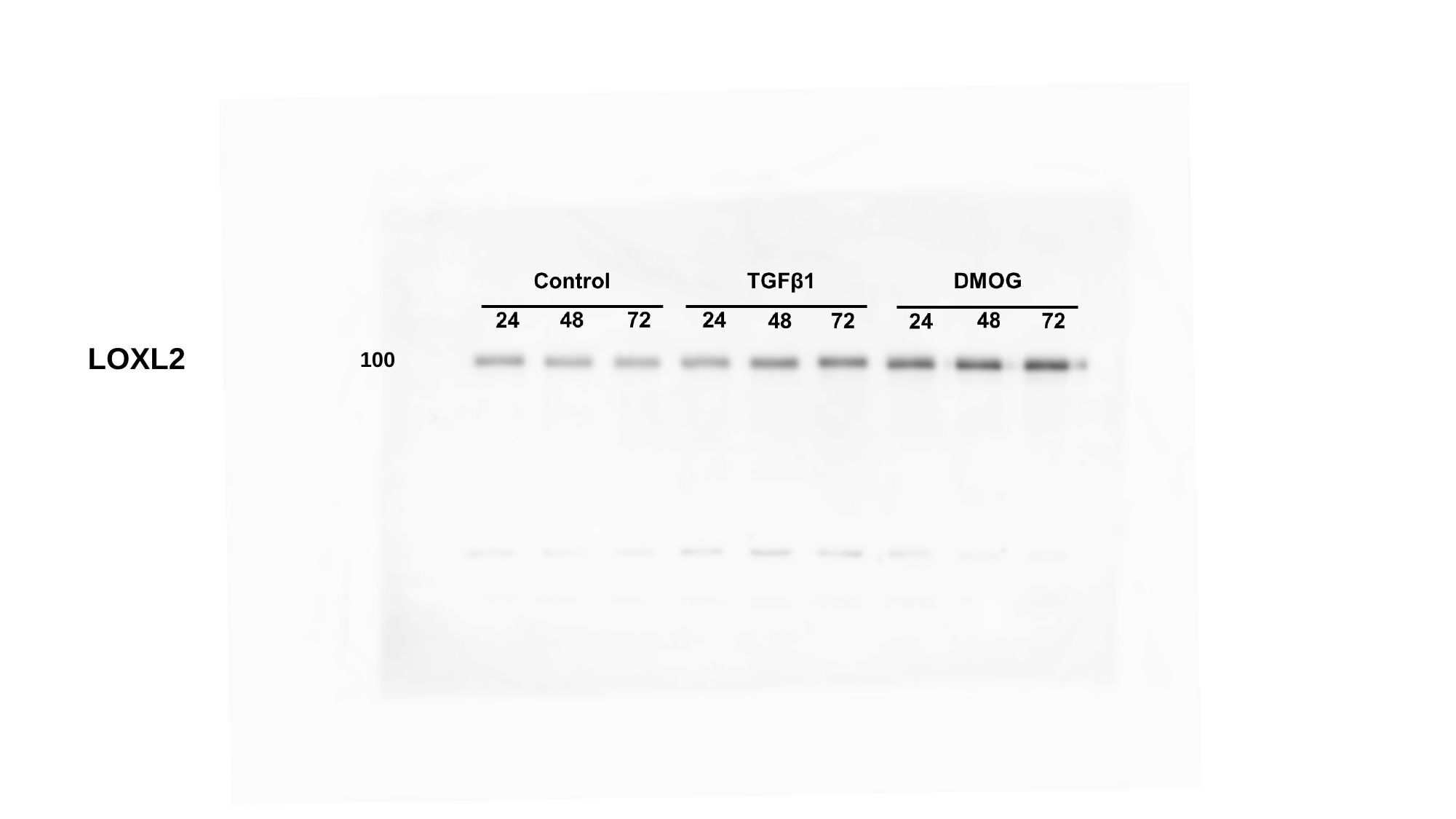

LOXL2
100

## Slide 3
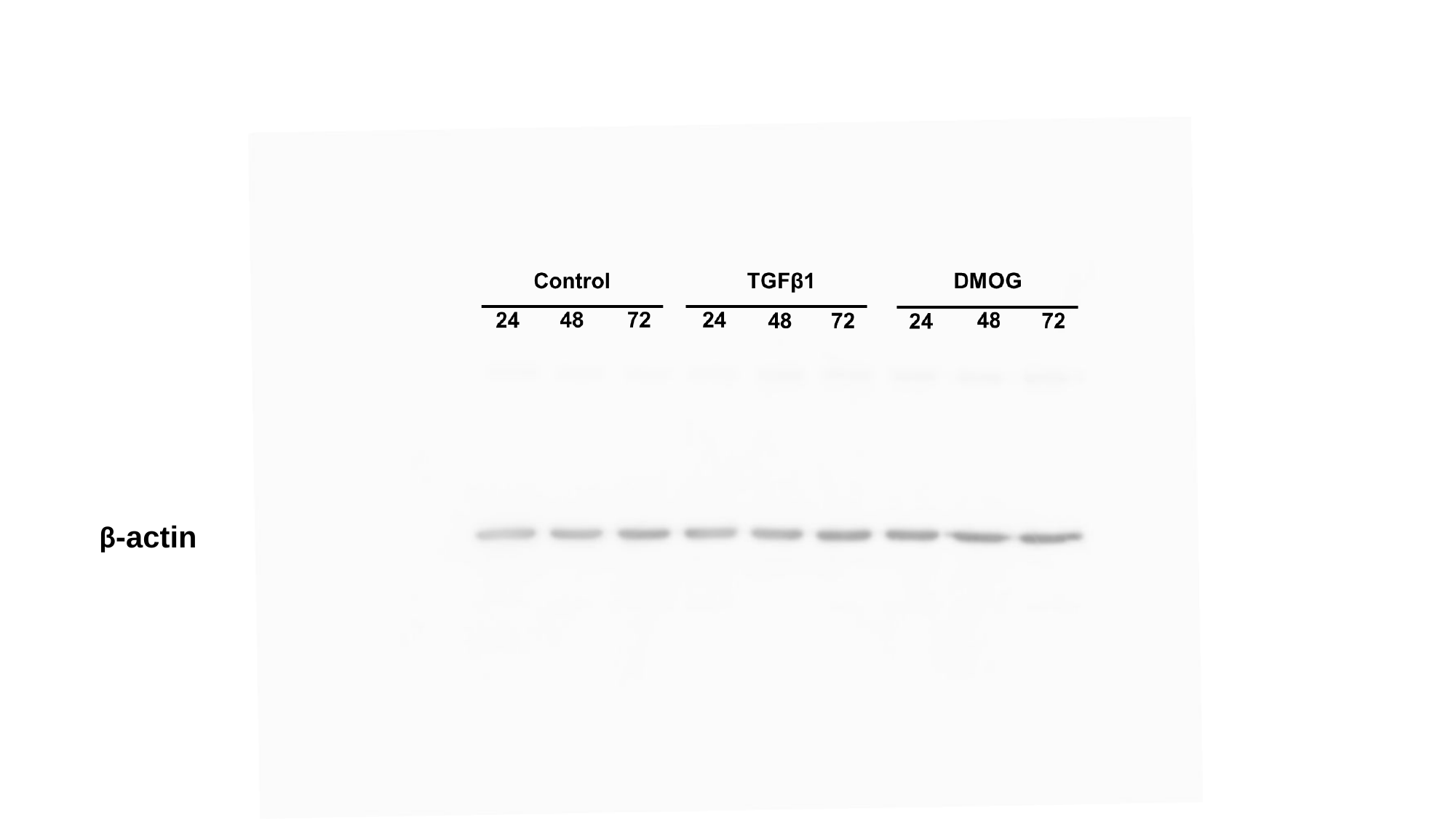

β-actin

Supplement: Figure 2—figure supplement 1—source data 1. [file elife-69348-fig2-figsupp1-data1.zip › FIgure2-figure supplement 1-source data 1/FIgure2-figure supplement 1-source data 1b/FIgure2-figure supplement 1-source data 1b labelled.pptx]

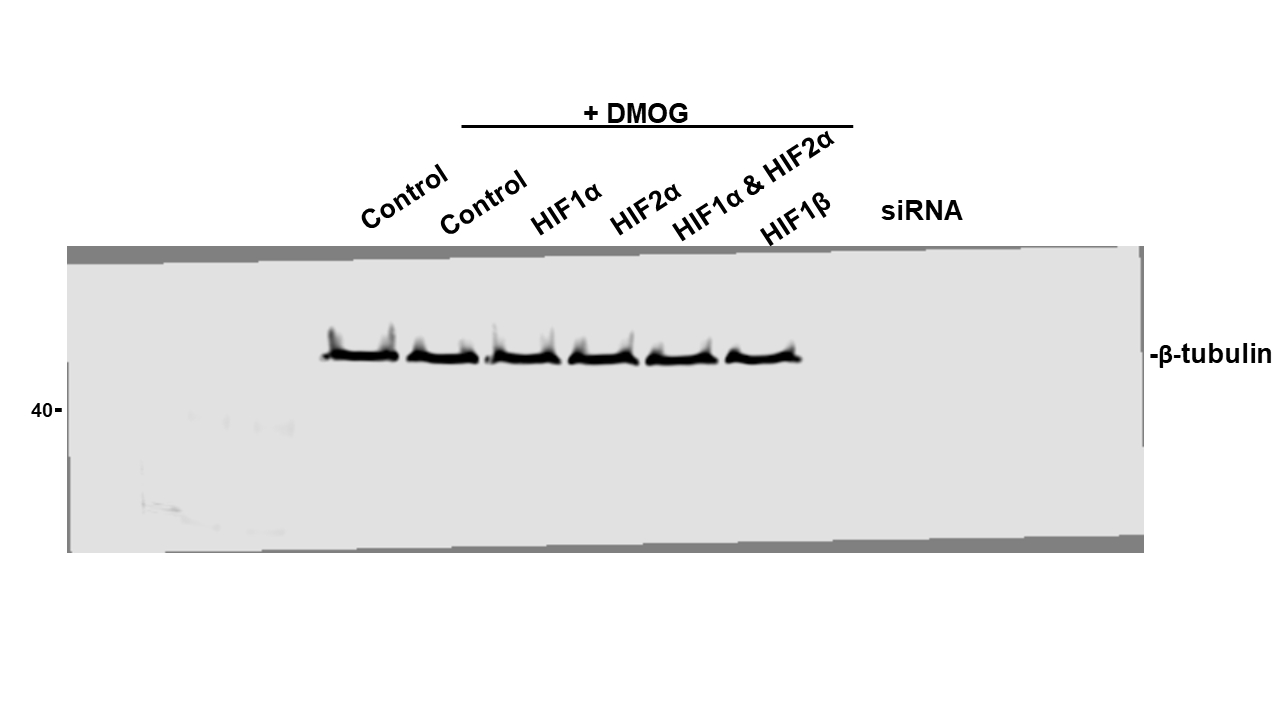

Supplement: Figure 3—source data 1. [file elife-69348-fig3-data1.zip › Figure 3-source data 1/labelled raw blot/BTUBULIN.TIF]

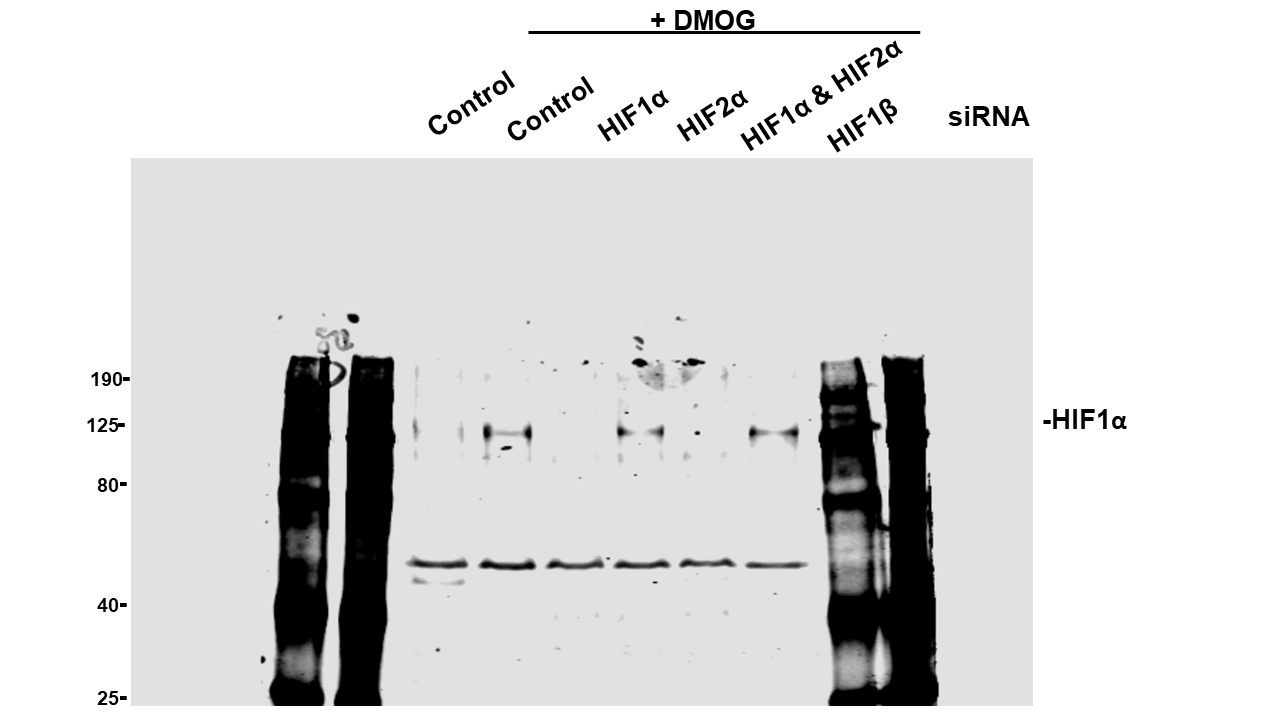

Supplement: Figure 3—source data 1. [file elife-69348-fig3-data1.zip › Figure 3-source data 1/labelled raw blot/HIF1A.TIF]

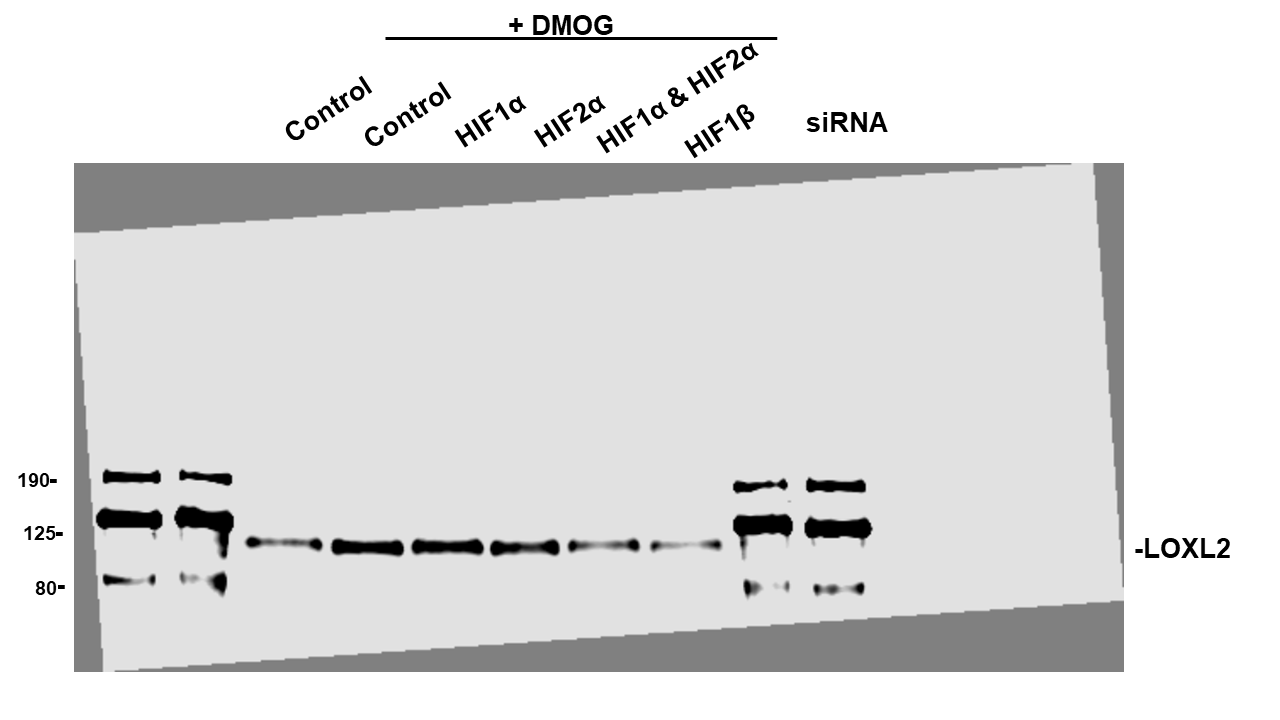

Supplement: Figure 3—source data 1. [file elife-69348-fig3-data1.zip › Figure 3-source data 1/labelled raw blot/LOXL2.TIF]

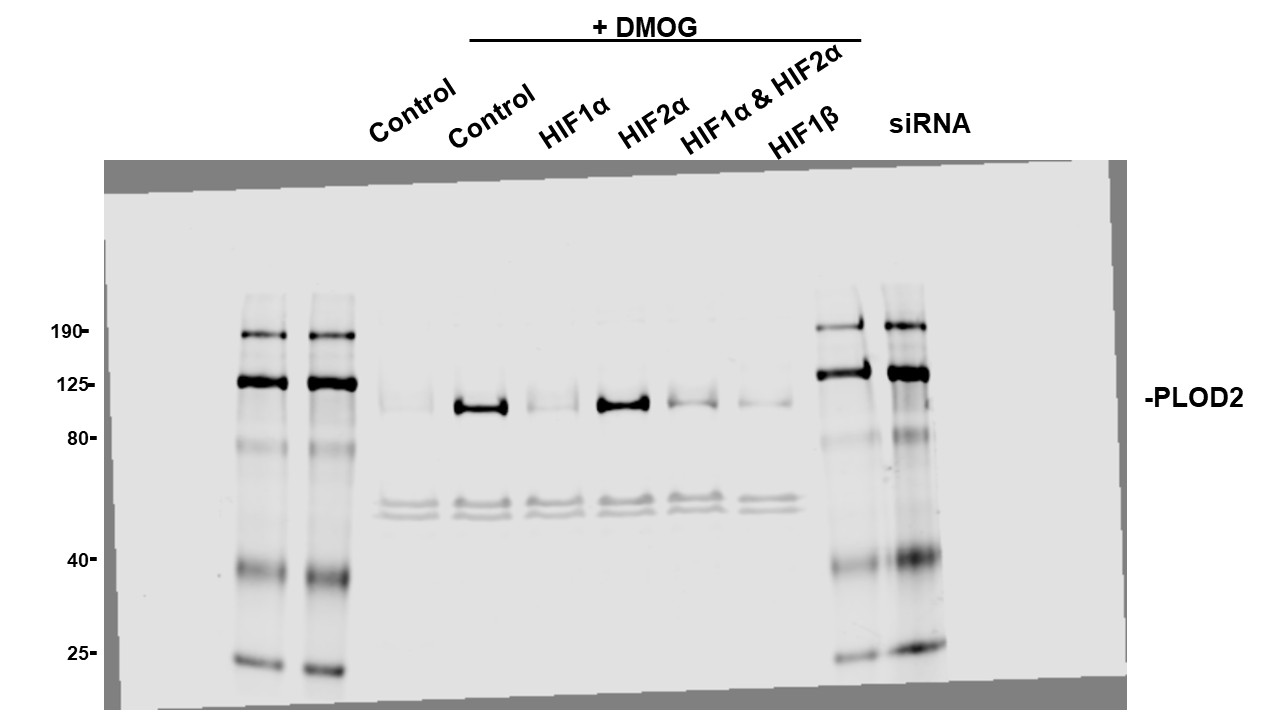

Supplement: Figure 3—source data 1. [file elife-69348-fig3-data1.zip › Figure 3-source data 1/labelled raw blot/PLOD2.TIF]

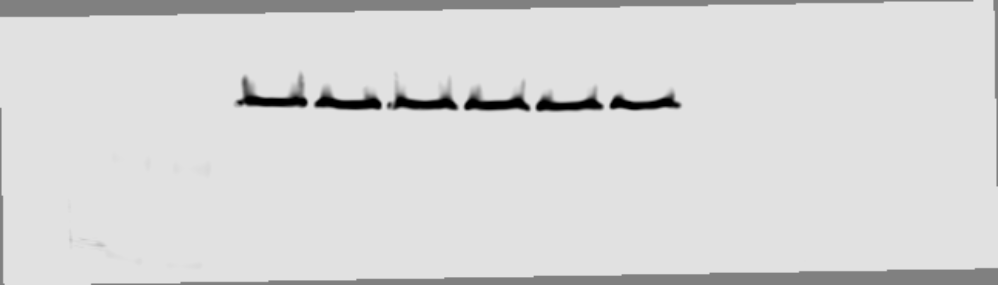

Supplement: Figure 3—source data 1. [file elife-69348-fig3-data1.zip › Figure 3-source data 1/raw blot/BTUBULIN.tif]

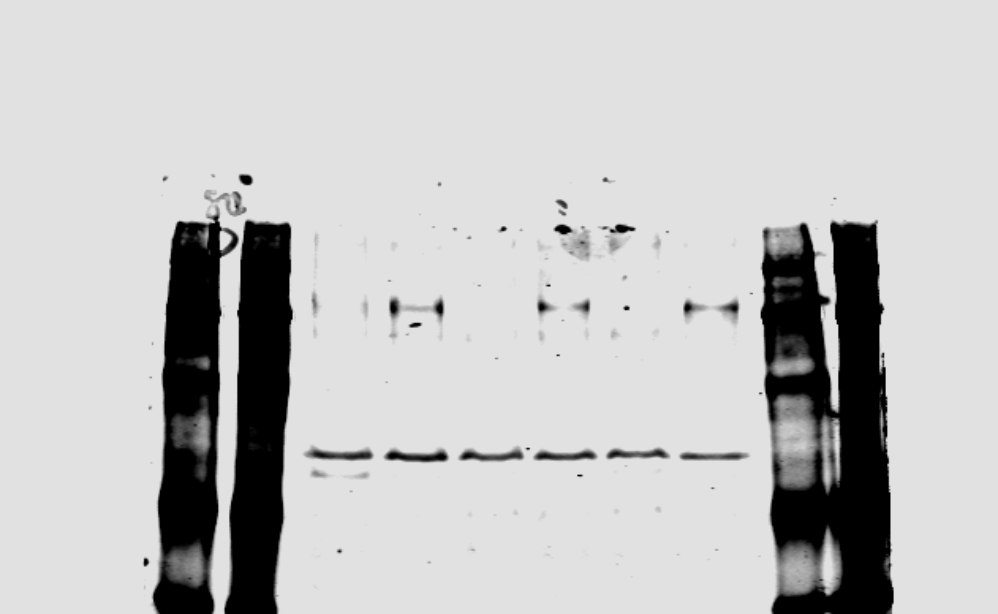

Supplement: Figure 3—source data 1. [file elife-69348-fig3-data1.zip › Figure 3-source data 1/raw blot/HIF1A.tif]

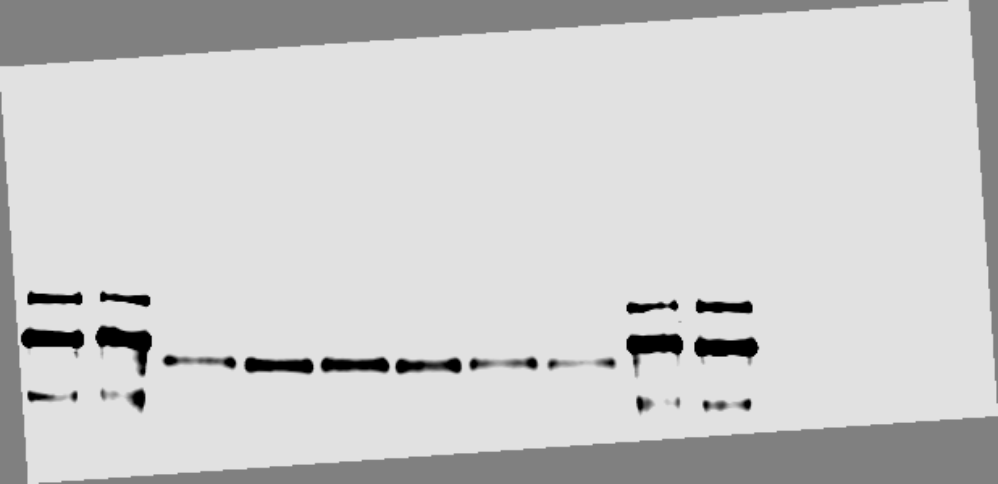

Supplement: Figure 3—source data 1. [file elife-69348-fig3-data1.zip › Figure 3-source data 1/raw blot/LOXL2.tif]

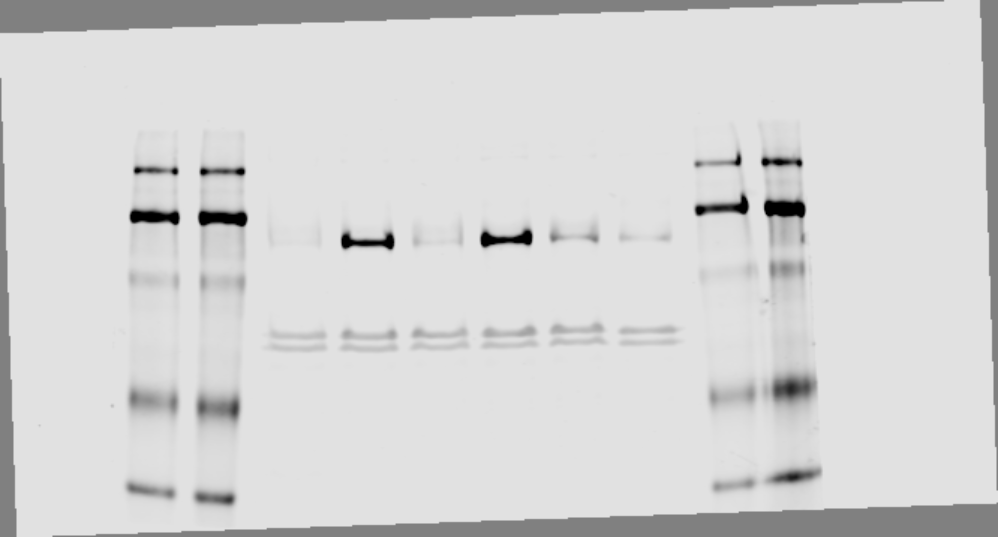

Supplement: Figure 3—source data 1. [file elife-69348-fig3-data1.zip › Figure 3-source data 1/raw blot/PLOD2.tif]

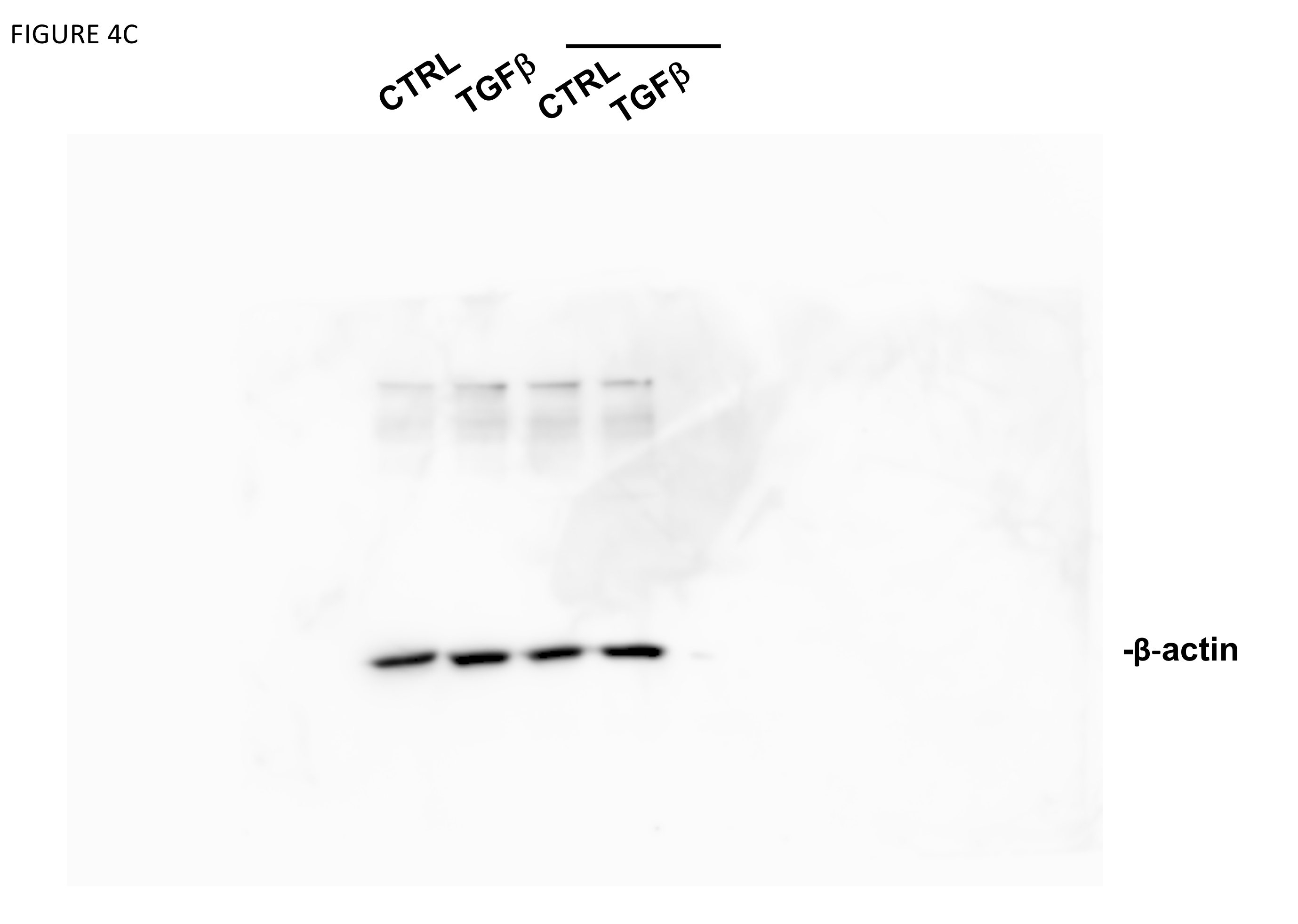

Supplement: Figure 4—source data 1. [file elife-69348-fig4-data1.zip › Figure 4-source data 1/Figure 4C/labelled raw blot/BETAACTIN.tiff]

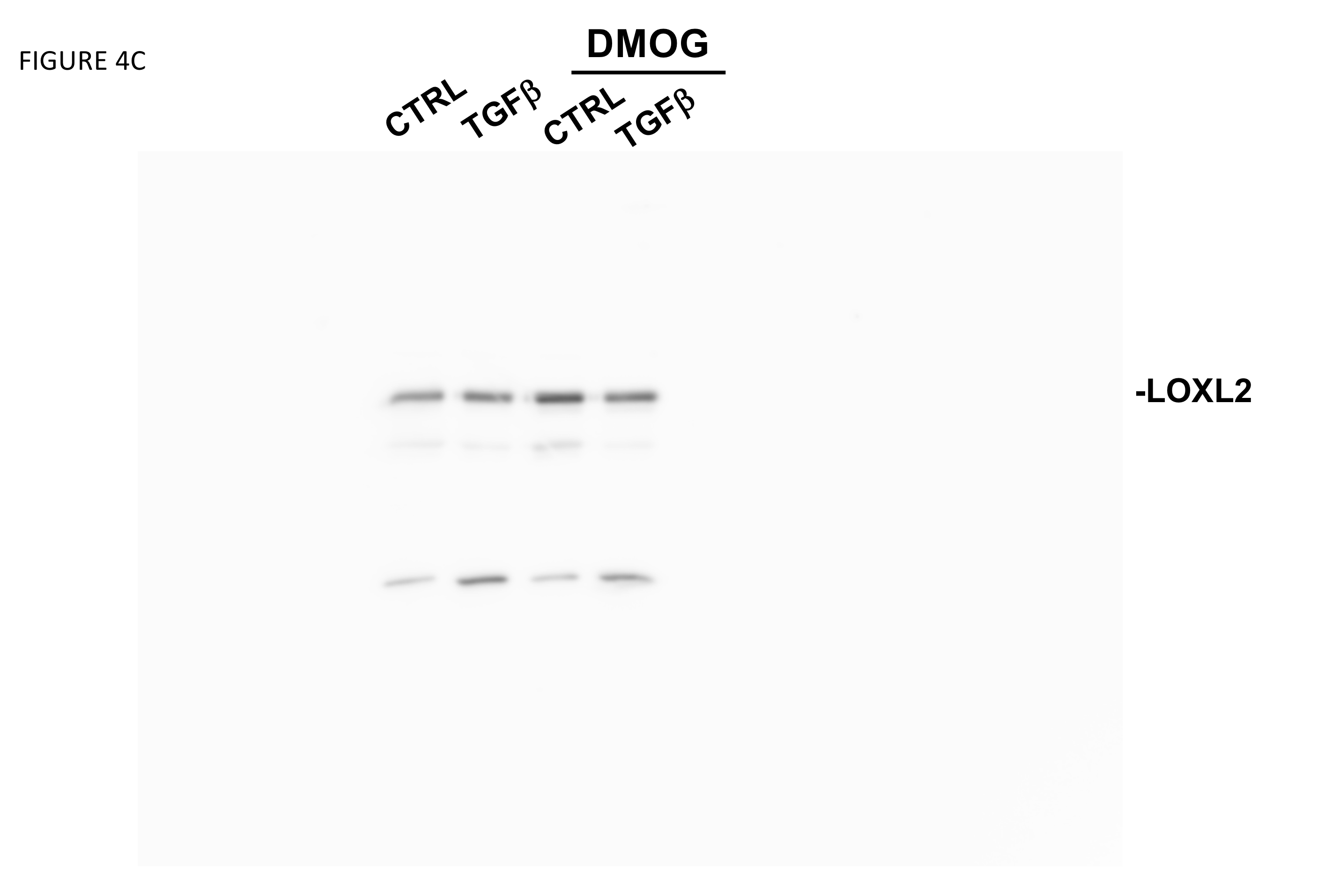

Supplement: Figure 4—source data 1. [file elife-69348-fig4-data1.zip › Figure 4-source data 1/Figure 4C/labelled raw blot/LOXL2.tiff]

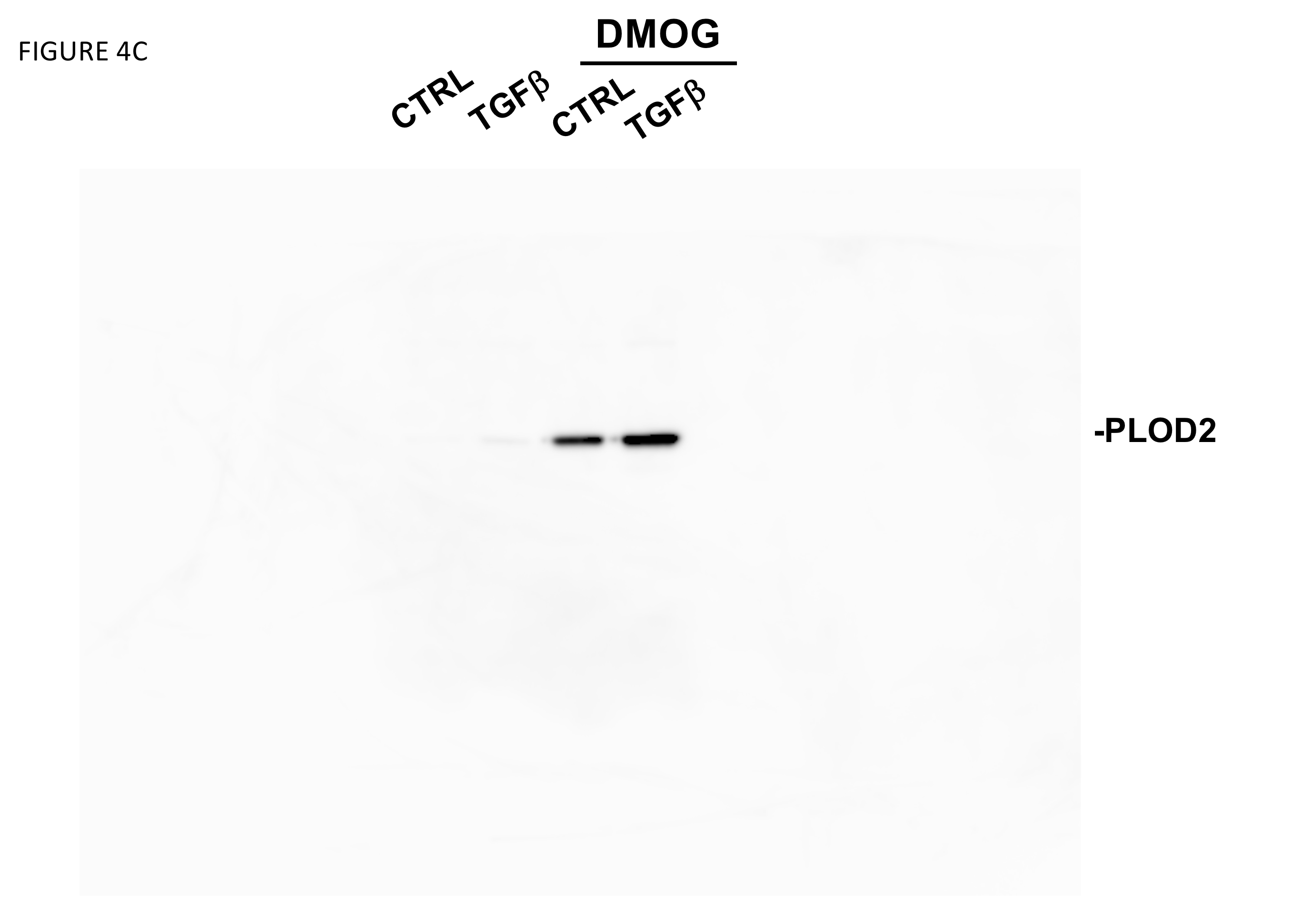

Supplement: Figure 4—source data 1. [file elife-69348-fig4-data1.zip › Figure 4-source data 1/Figure 4C/labelled raw blot/PLOD2.tiff]

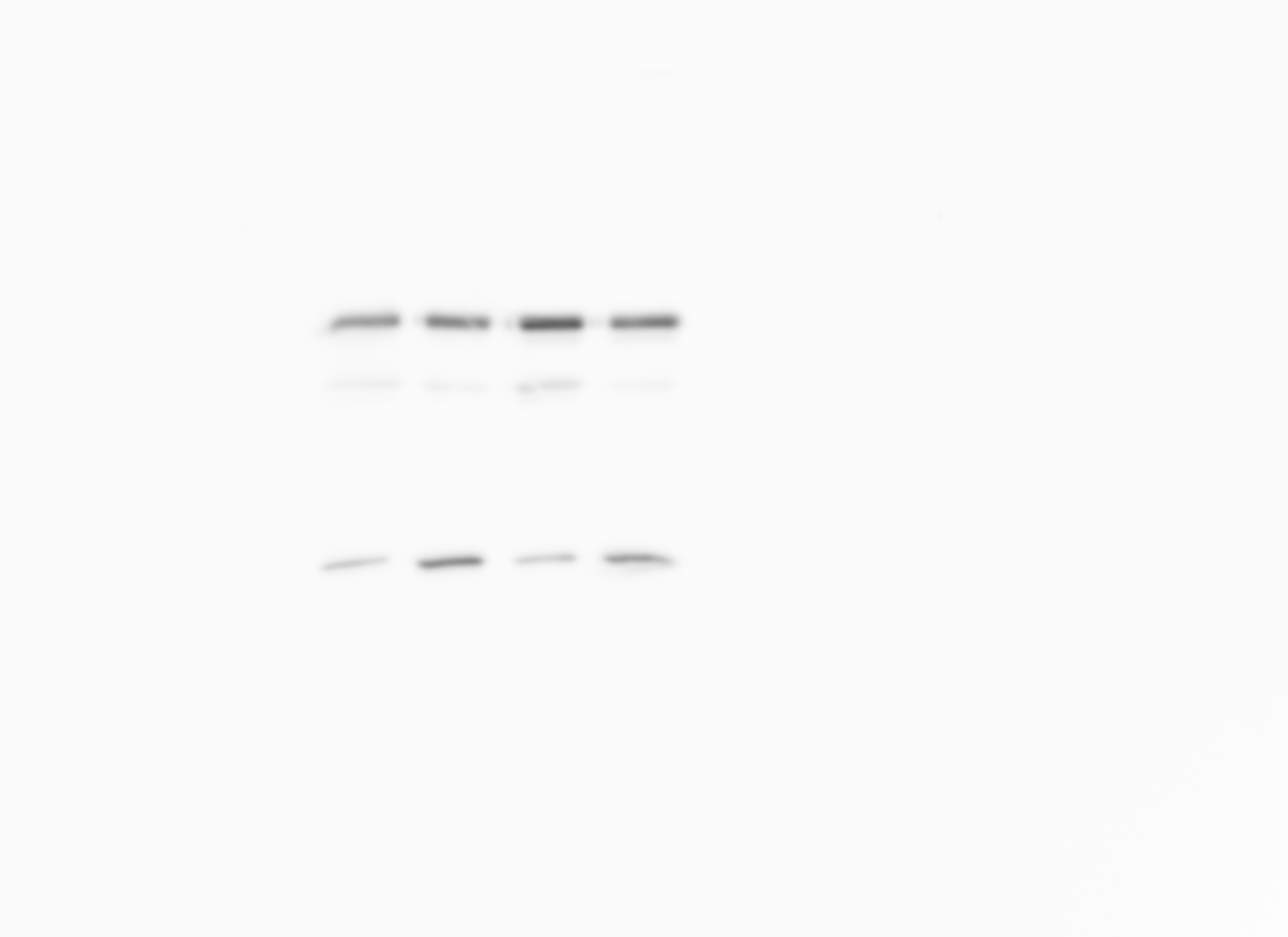

Supplement: Figure 4—source data 1. [file elife-69348-fig4-data1.zip › Figure 4-source data 1/Figure 4C/raw blot/LOXL2.tif]

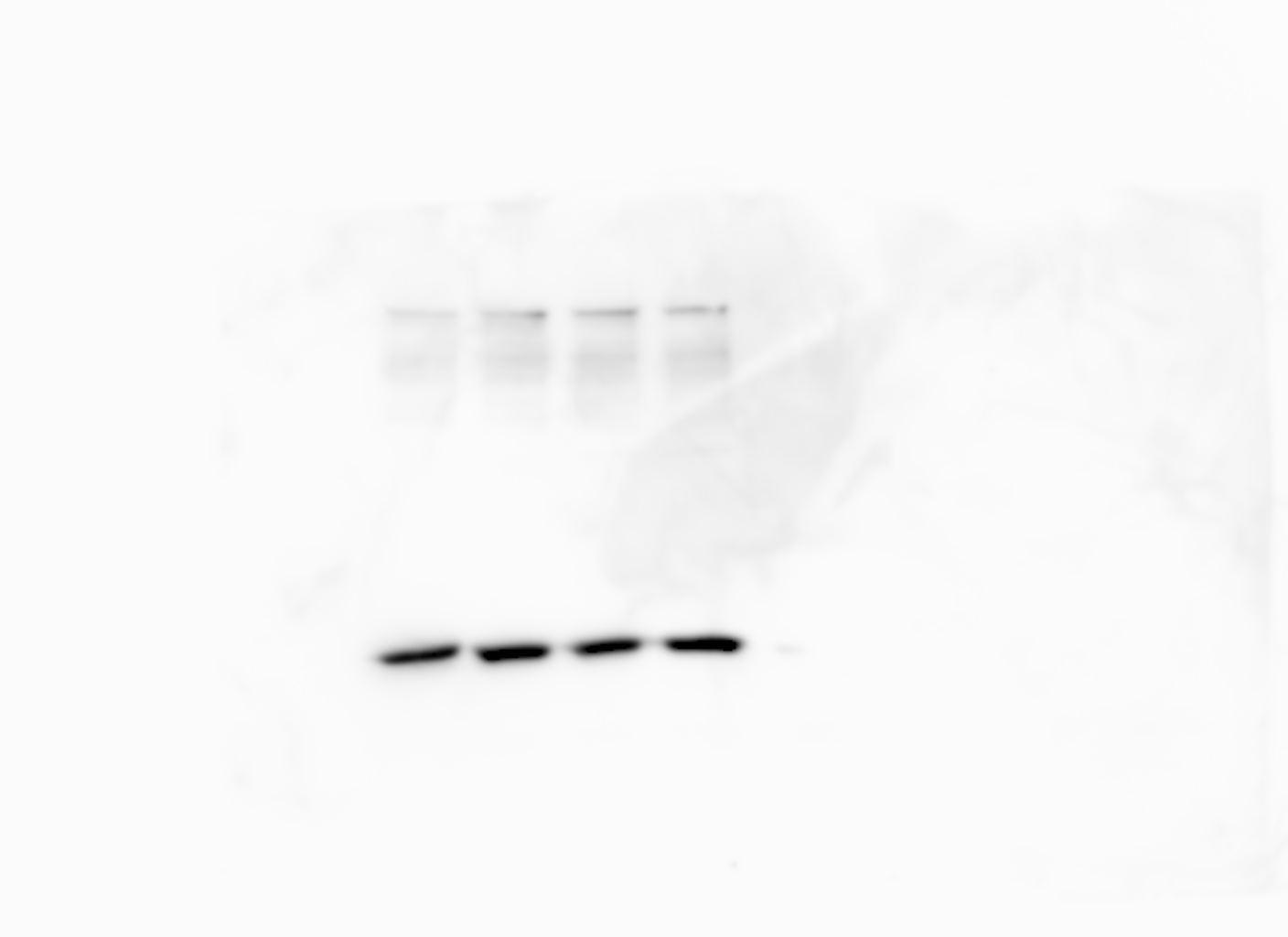

Supplement: Figure 4—source data 1. [file elife-69348-fig4-data1.zip › Figure 4-source data 1/Figure 4C/raw blot/BETAACTIN.tif]

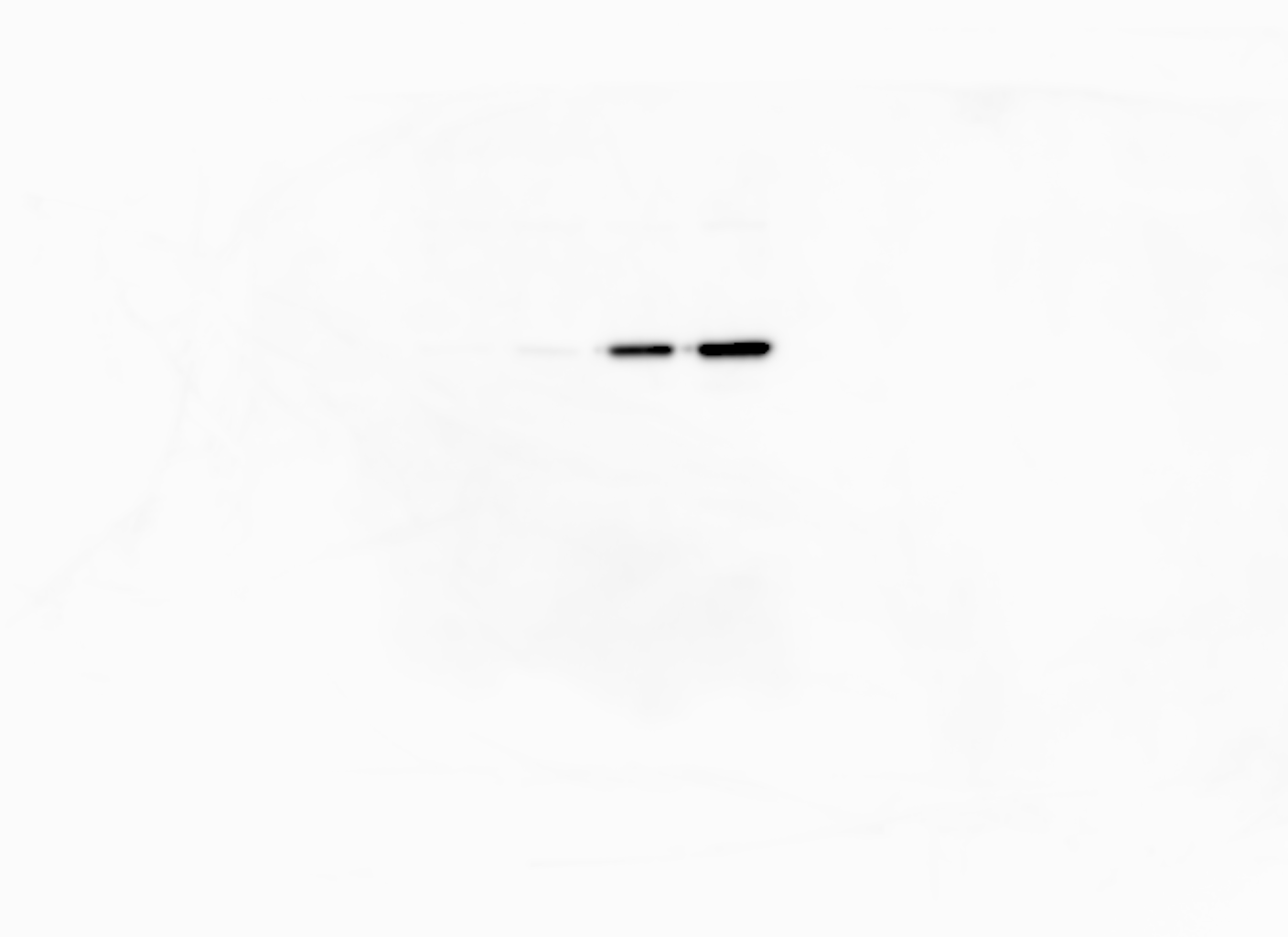

Supplement: Figure 4—source data 1. [file elife-69348-fig4-data1.zip › Figure 4-source data 1/Figure 4C/raw blot/PLOD2.tif]

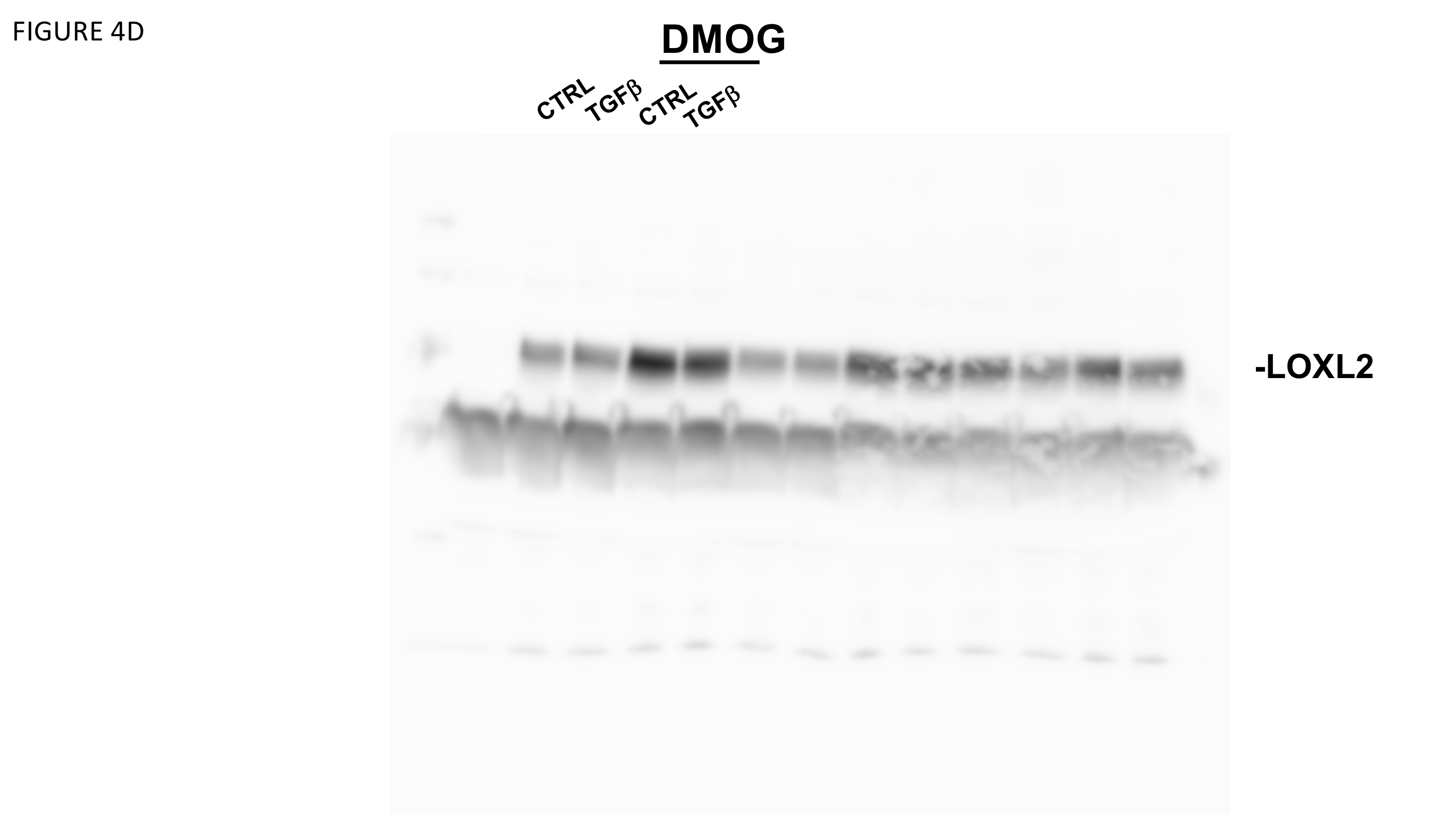

Supplement: Figure 4—source data 1. [file elife-69348-fig4-data1.zip › Figure 4-source data 1/Figure 4D/labelled raw blot/loxl2.tiff]

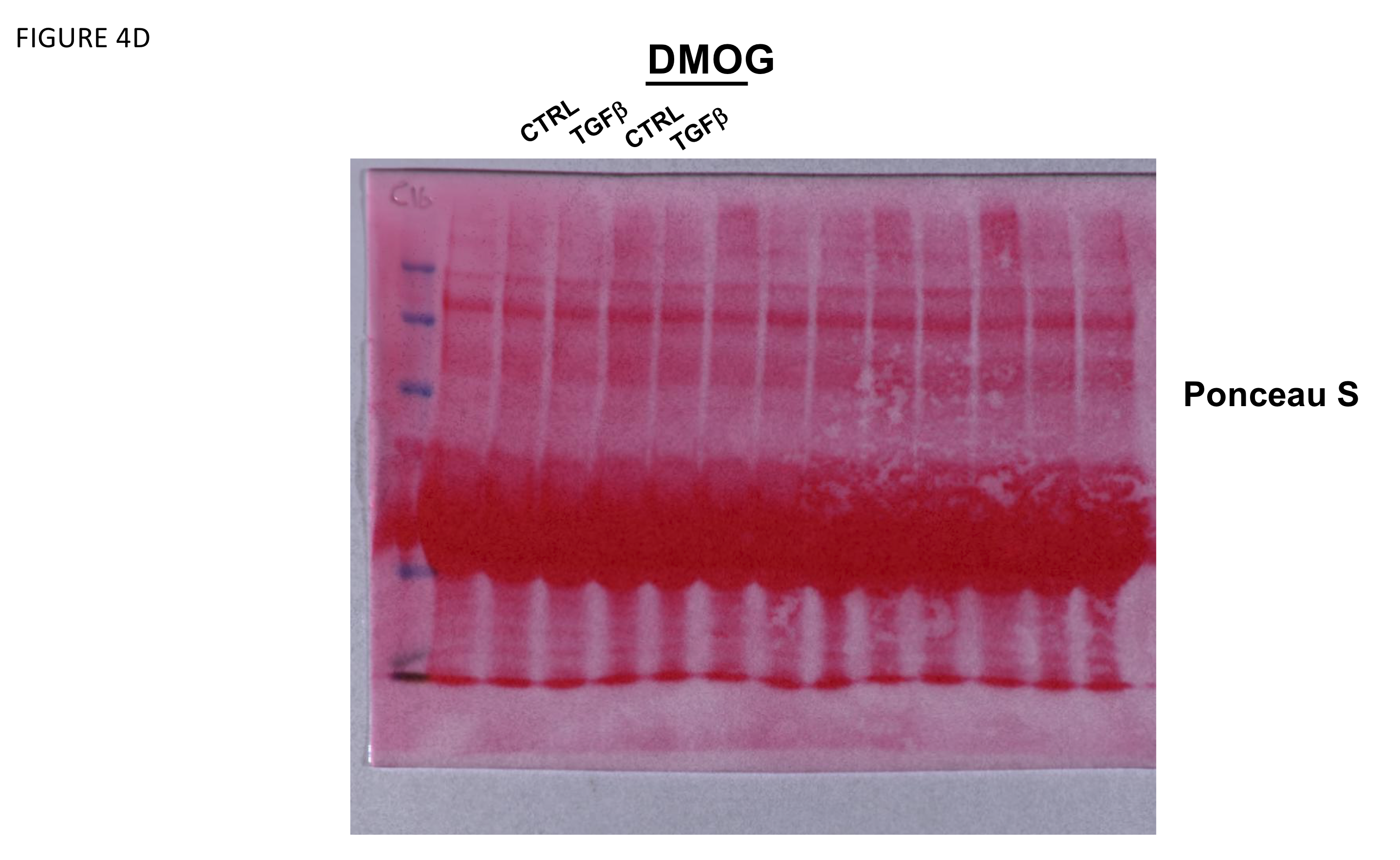

Supplement: Figure 4—source data 1. [file elife-69348-fig4-data1.zip › Figure 4-source data 1/Figure 4D/labelled raw blot/ponseaus.tiff]

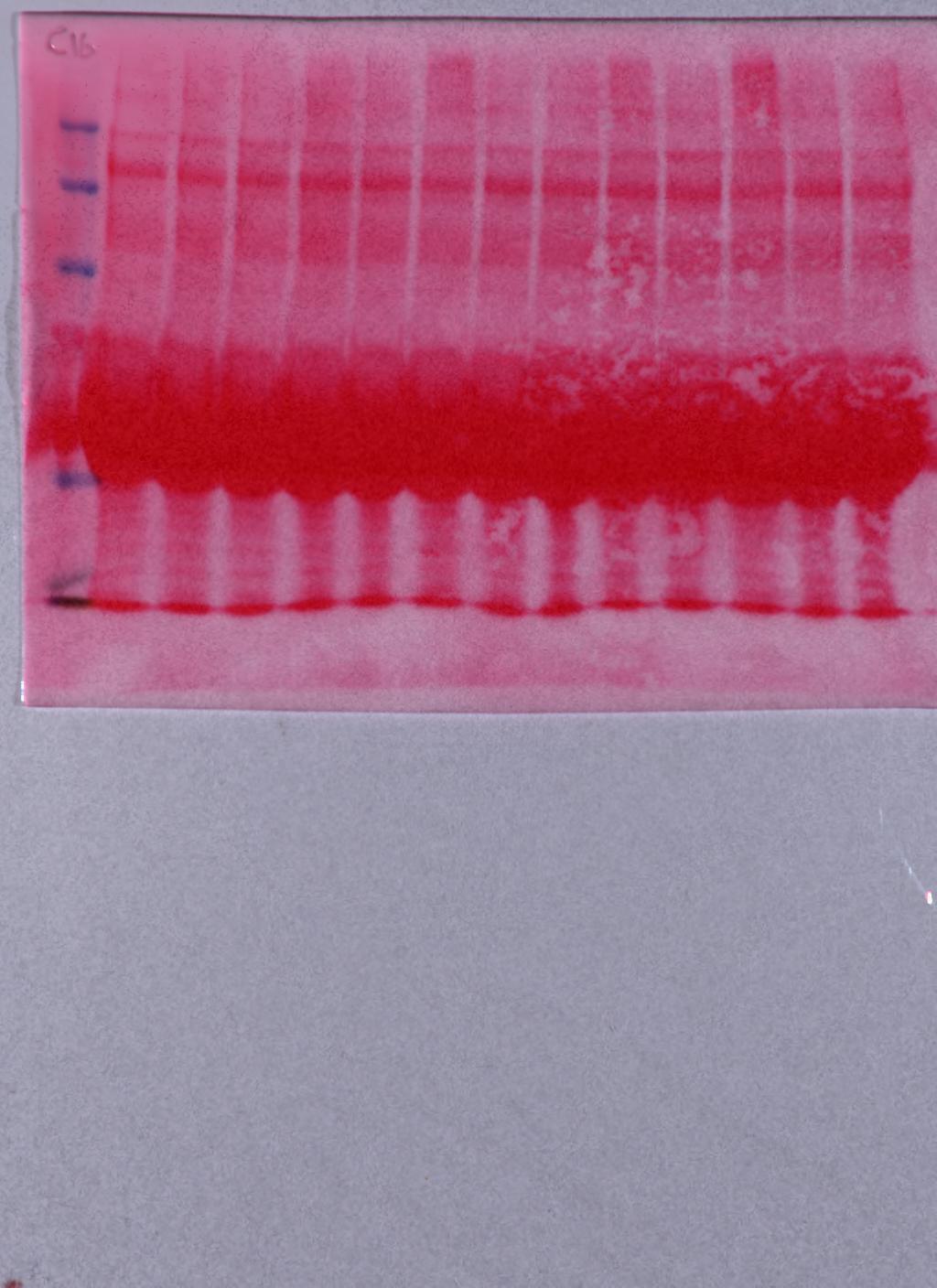

Supplement: Figure 4—source data 1. [file elife-69348-fig4-data1.zip › Figure 4-source data 1/Figure 4D/raw blot/PONSEAUS.jpg]

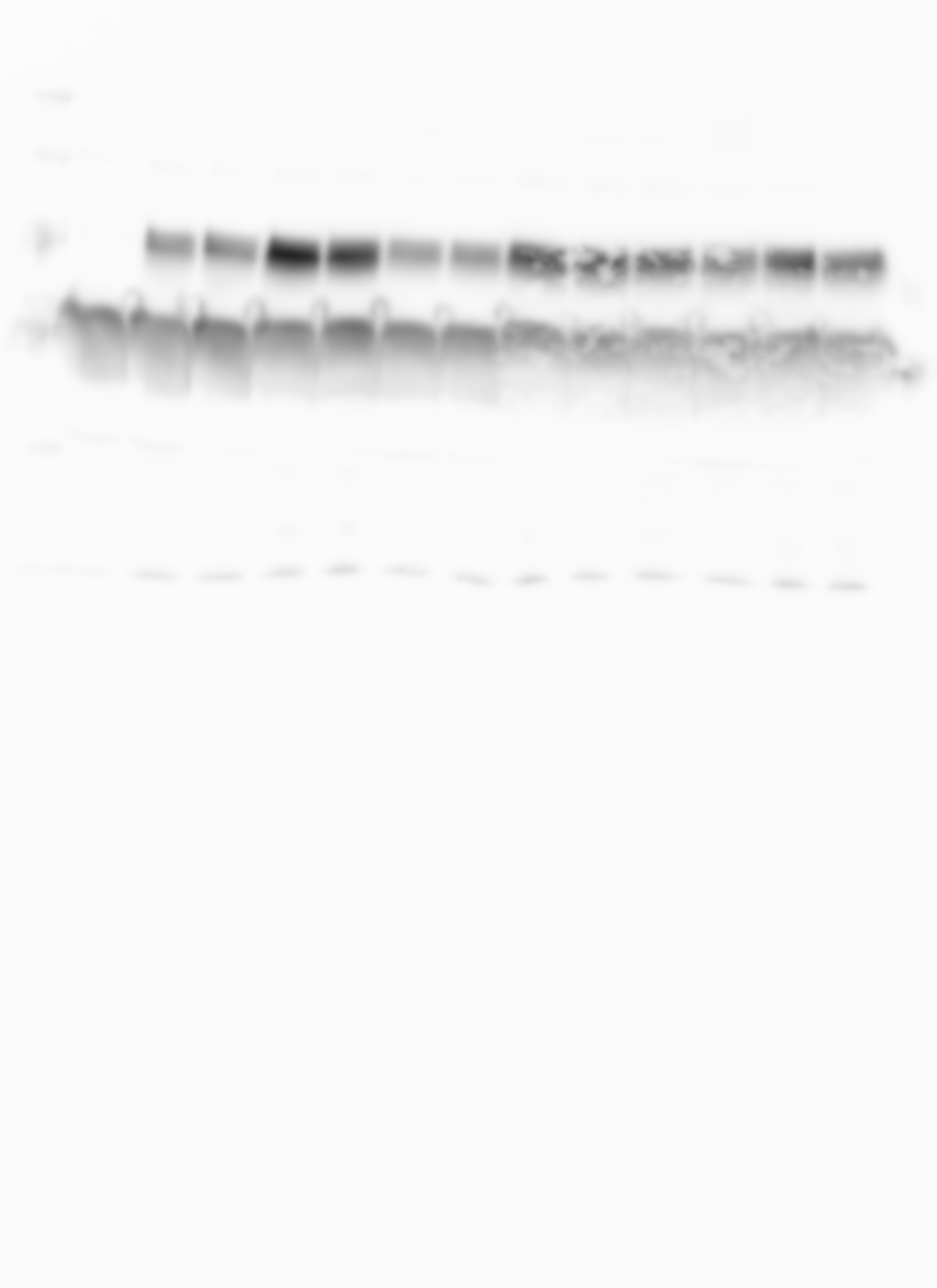

Supplement: Figure 4—source data 1. [file elife-69348-fig4-data1.zip › Figure 4-source data 1/Figure 4D/raw blot/LOXL2SECRETED.tif]

## Slide 1
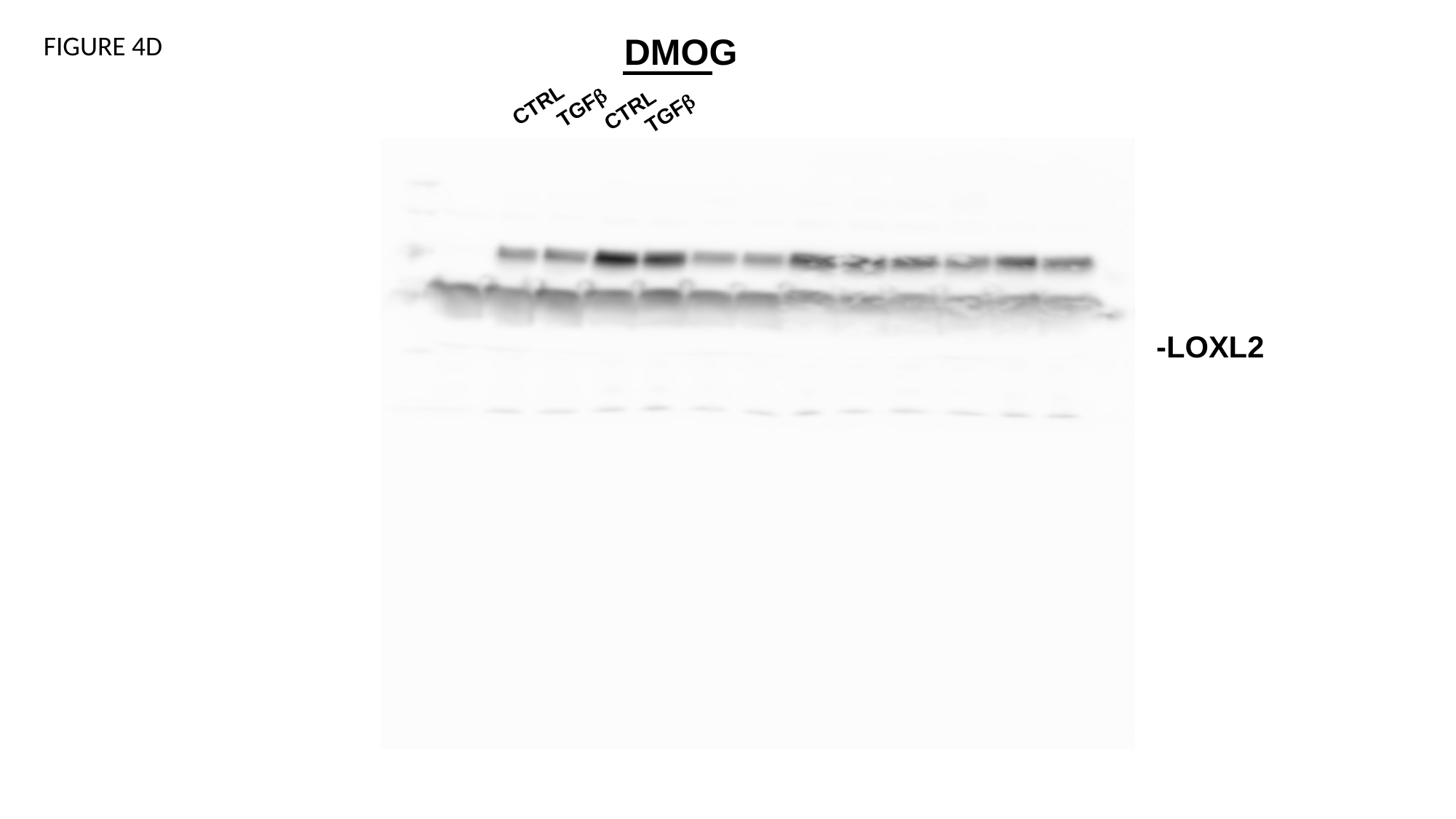

FIGURE 4D
DMOG
 TGFb
 CTRL
 TGFb
 CTRL
-LOXL2

## Slide 2
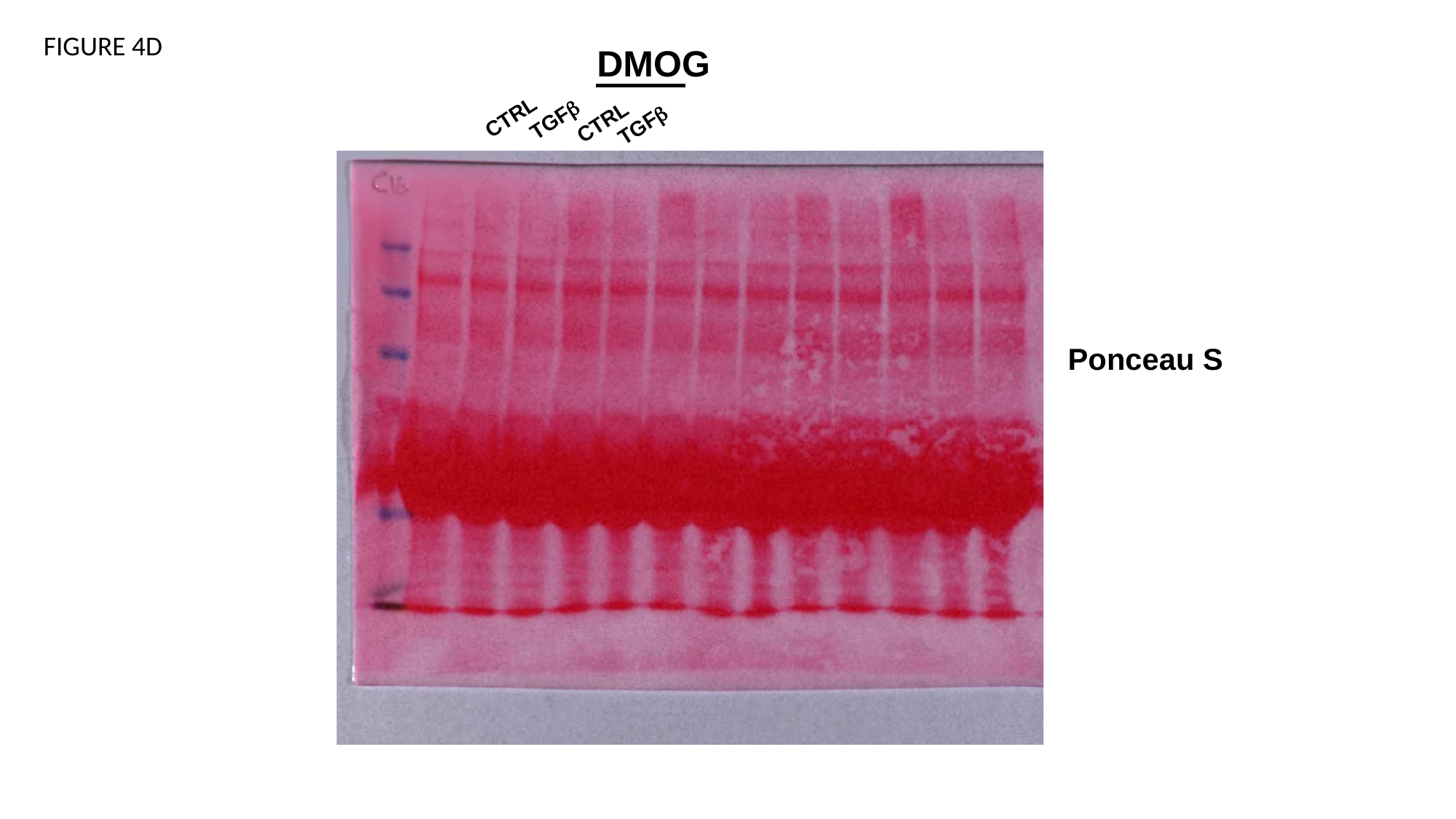

FIGURE 4D
DMOG
 TGFb
 CTRL
 TGFb
 CTRL
Ponceau S

Supplement: Figure 4—source data 1. [file elife-69348-fig4-data1.zip › Figure 4-source data 1/Figure 4D/Figure 4D labelled.pptx]

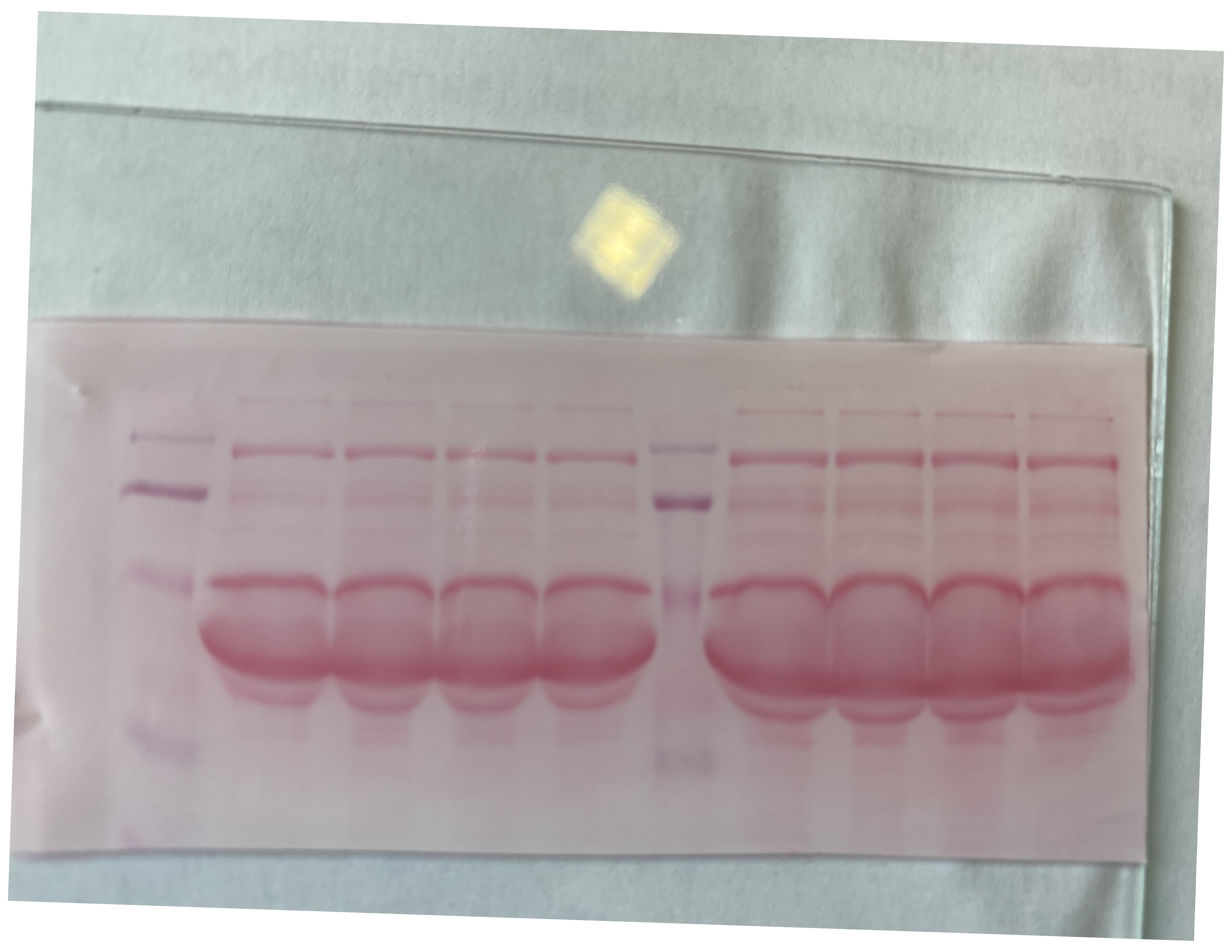

Supplement: Figure 4—figure supplement 1—source data 1. [file elife-69348-fig4-figsupp1-data1.zip › Figure 4-figure supplement 1ΓÇösource data 1/raw blot/Ponceau S.tif]

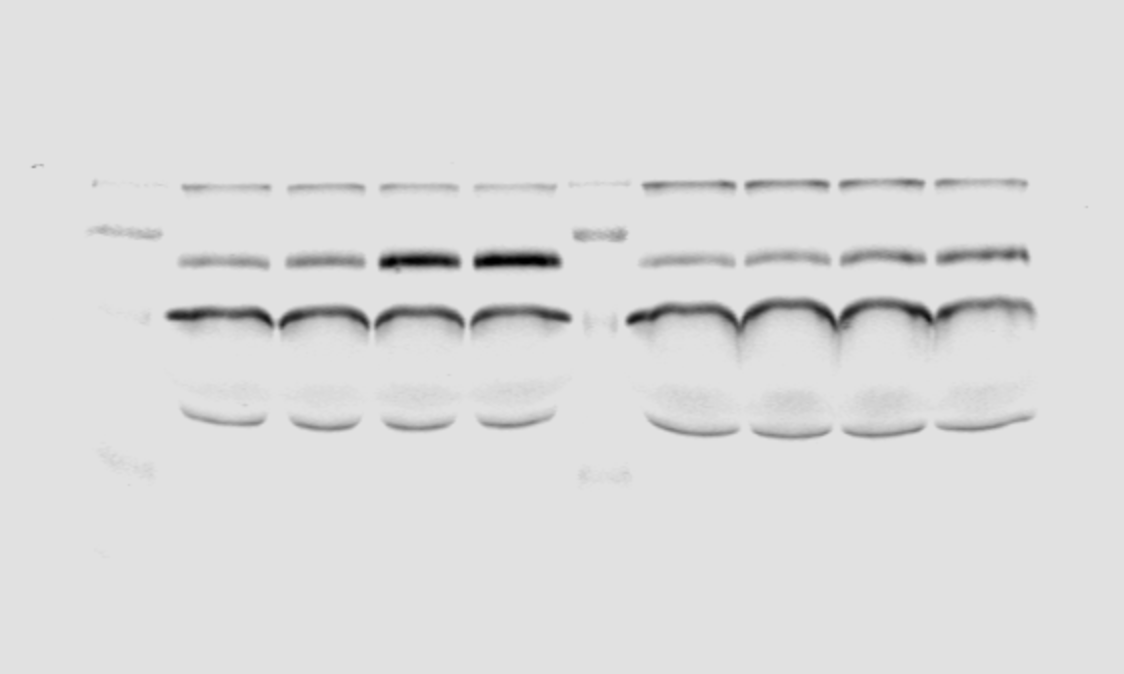

Supplement: Figure 4—figure supplement 1—source data 1. [file elife-69348-fig4-figsupp1-data1.zip › Figure 4-figure supplement 1ΓÇösource data 1/raw blot/LOXL2.tif]

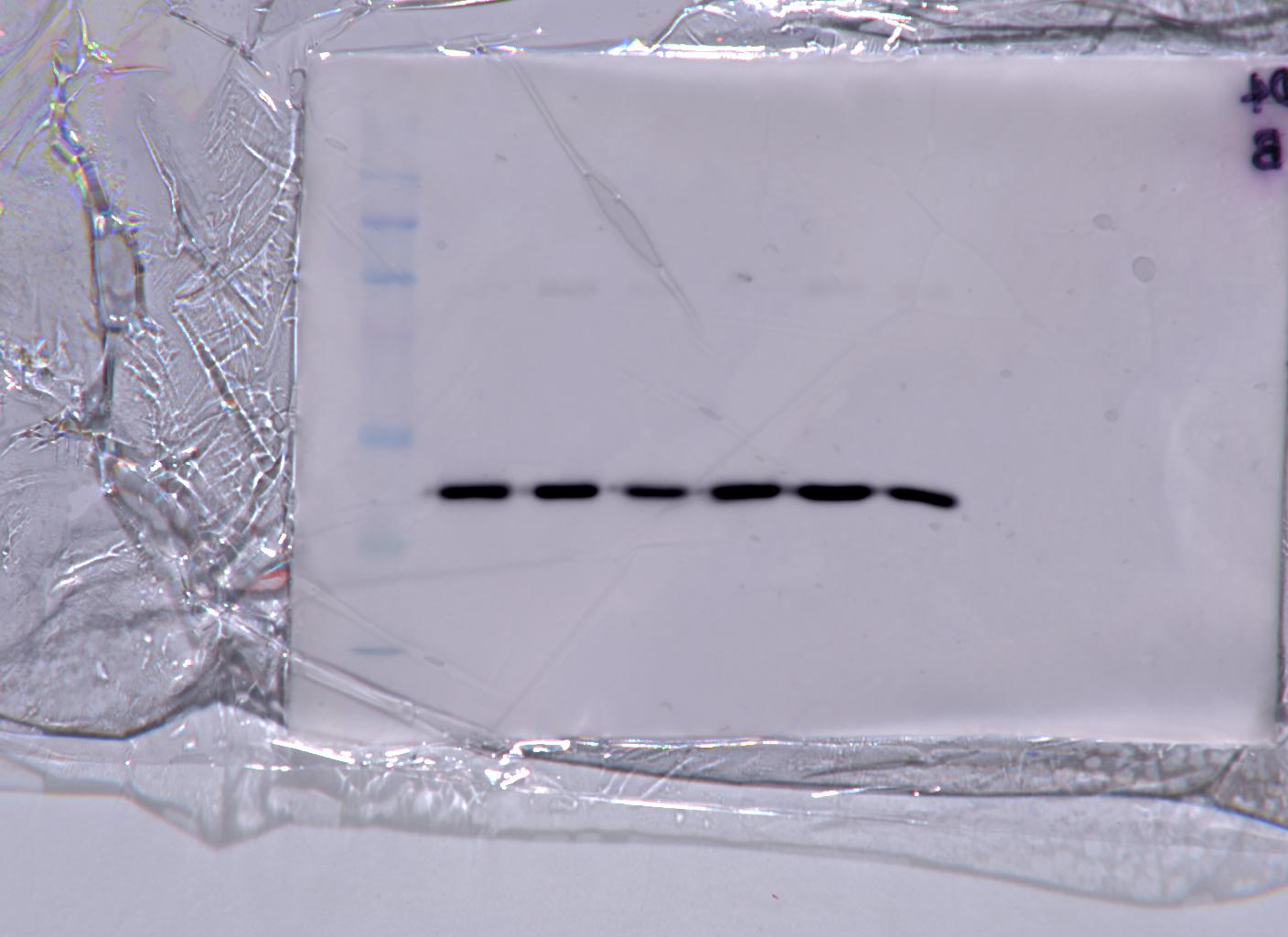

Supplement: Figure 5—figure supplement 1—source data 1. [file elife-69348-fig5-figsupp1-data1.zip › Figure 5ΓÇöfigure supplement 1-source data 1/Figure 5ΓÇöfigure supplement 1-source data 1b/Raw blot/B-actin with MW ladder.tif]

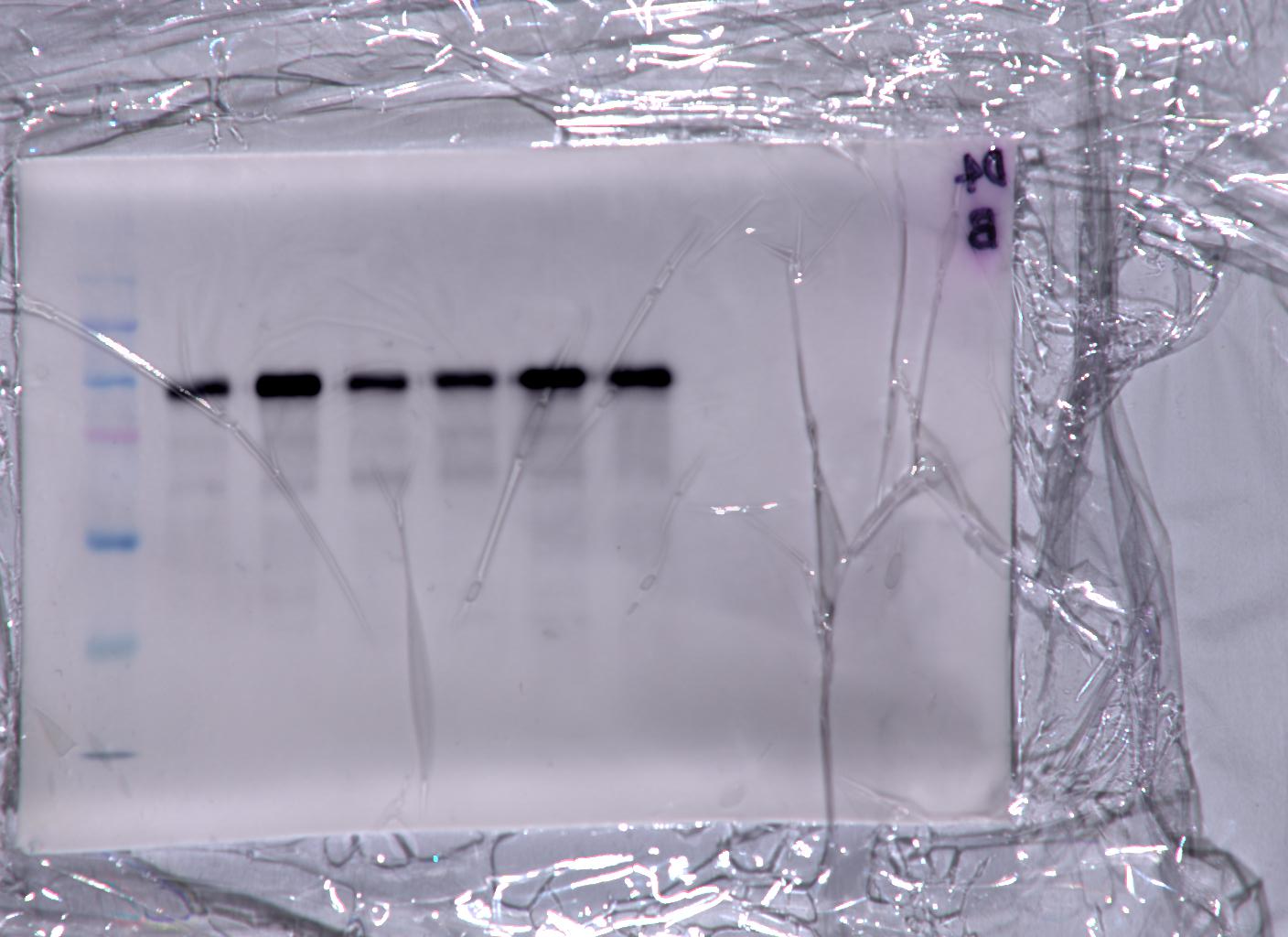

Supplement: Figure 5—figure supplement 1—source data 1. [file elife-69348-fig5-figsupp1-data1.zip › Figure 5ΓÇöfigure supplement 1-source data 1/Figure 5ΓÇöfigure supplement 1-source data 1b/Raw blot/LOXL2 with MW ladder.tif]

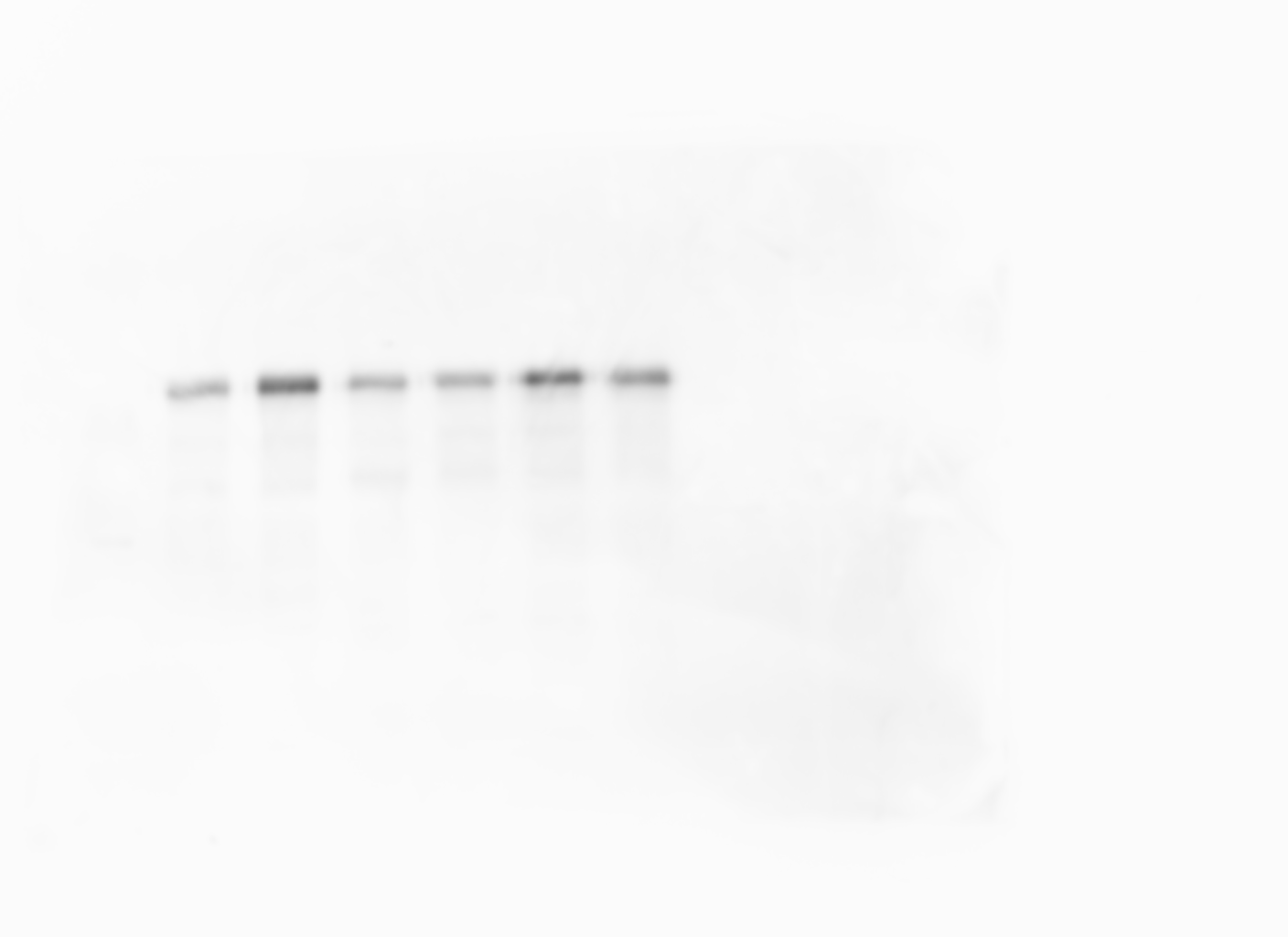

Supplement: Figure 5—figure supplement 1—source data 1. [file elife-69348-fig5-figsupp1-data1.zip › Figure 5ΓÇöfigure supplement 1-source data 1/Figure 5ΓÇöfigure supplement 1-source data 1b/Raw blot/LOXL2 raw.tif]

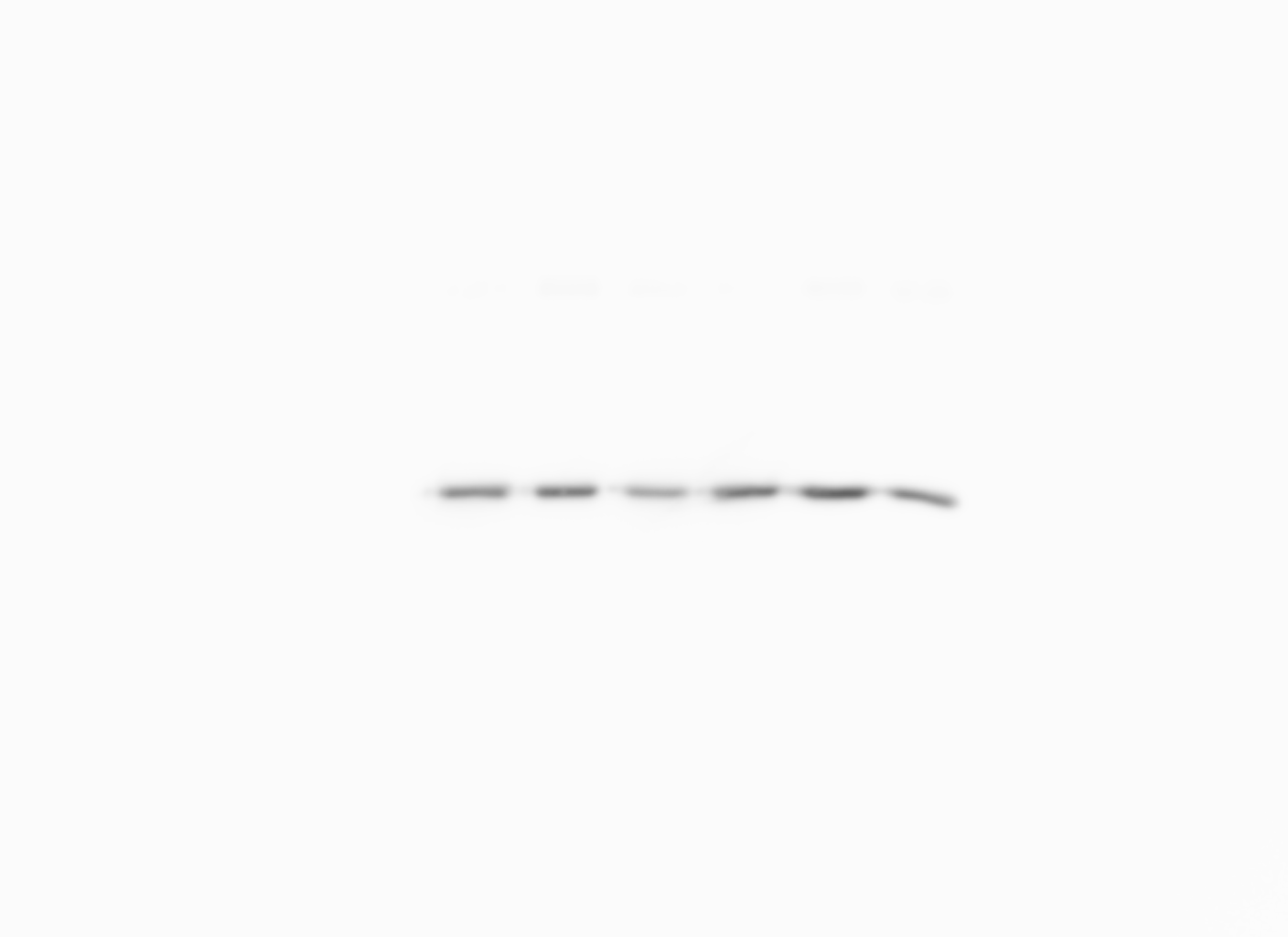

Supplement: Figure 5—figure supplement 1—source data 1. [file elife-69348-fig5-figsupp1-data1.zip › Figure 5ΓÇöfigure supplement 1-source data 1/Figure 5ΓÇöfigure supplement 1-source data 1b/Raw blot/B-actin raw.tif]

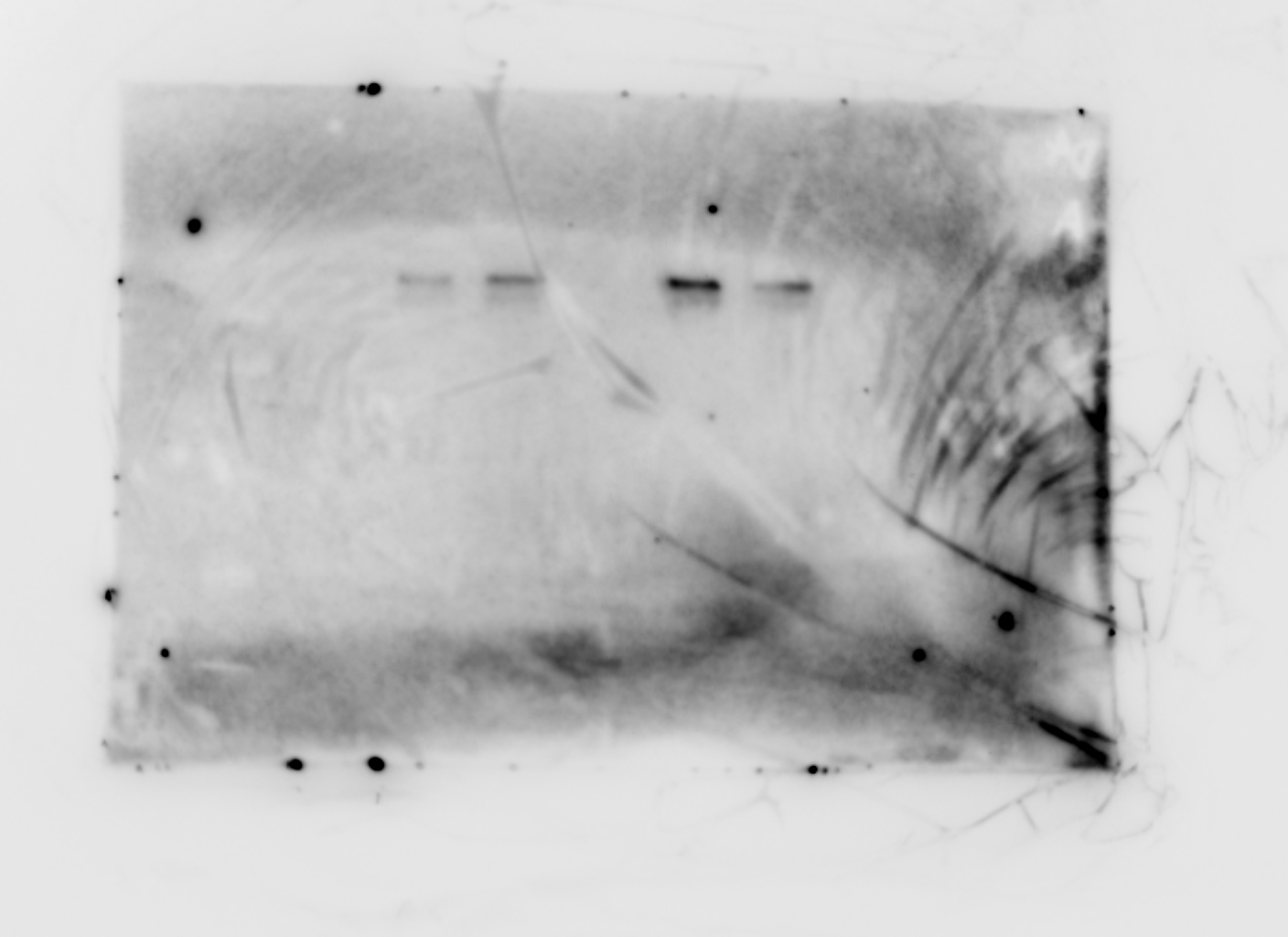

Supplement: Figure 5—figure supplement 1—source data 1. [file elife-69348-fig5-figsupp1-data1.zip › Figure 5ΓÇöfigure supplement 1-source data 1/Figure 5ΓÇöfigure supplement 1-source data 1a/Raw blot/HIF1A raw.tif]

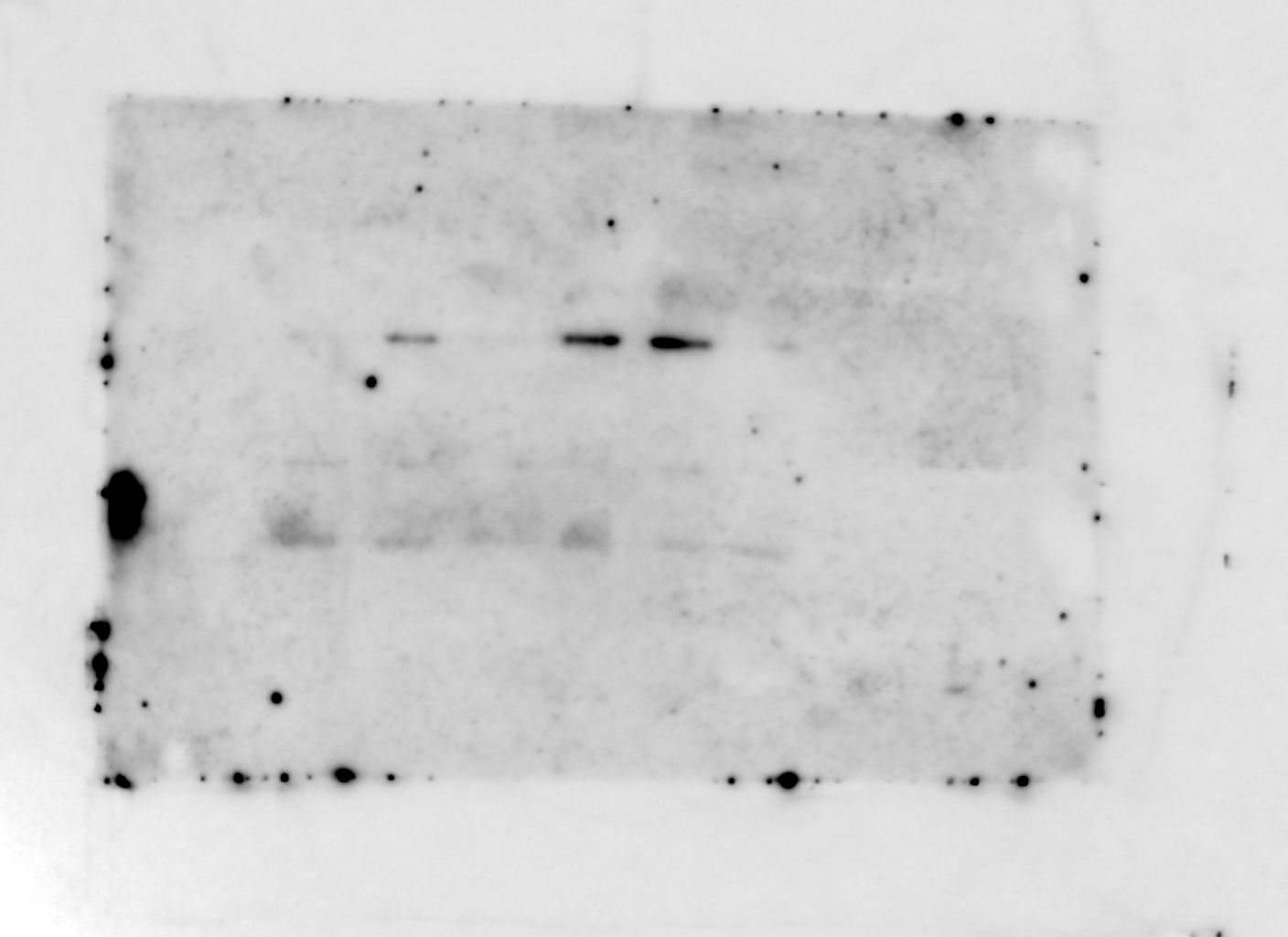

Supplement: Figure 5—figure supplement 1—source data 1. [file elife-69348-fig5-figsupp1-data1.zip › Figure 5ΓÇöfigure supplement 1-source data 1/Figure 5ΓÇöfigure supplement 1-source data 1a/Raw blot/PLOD2 raw.tif]

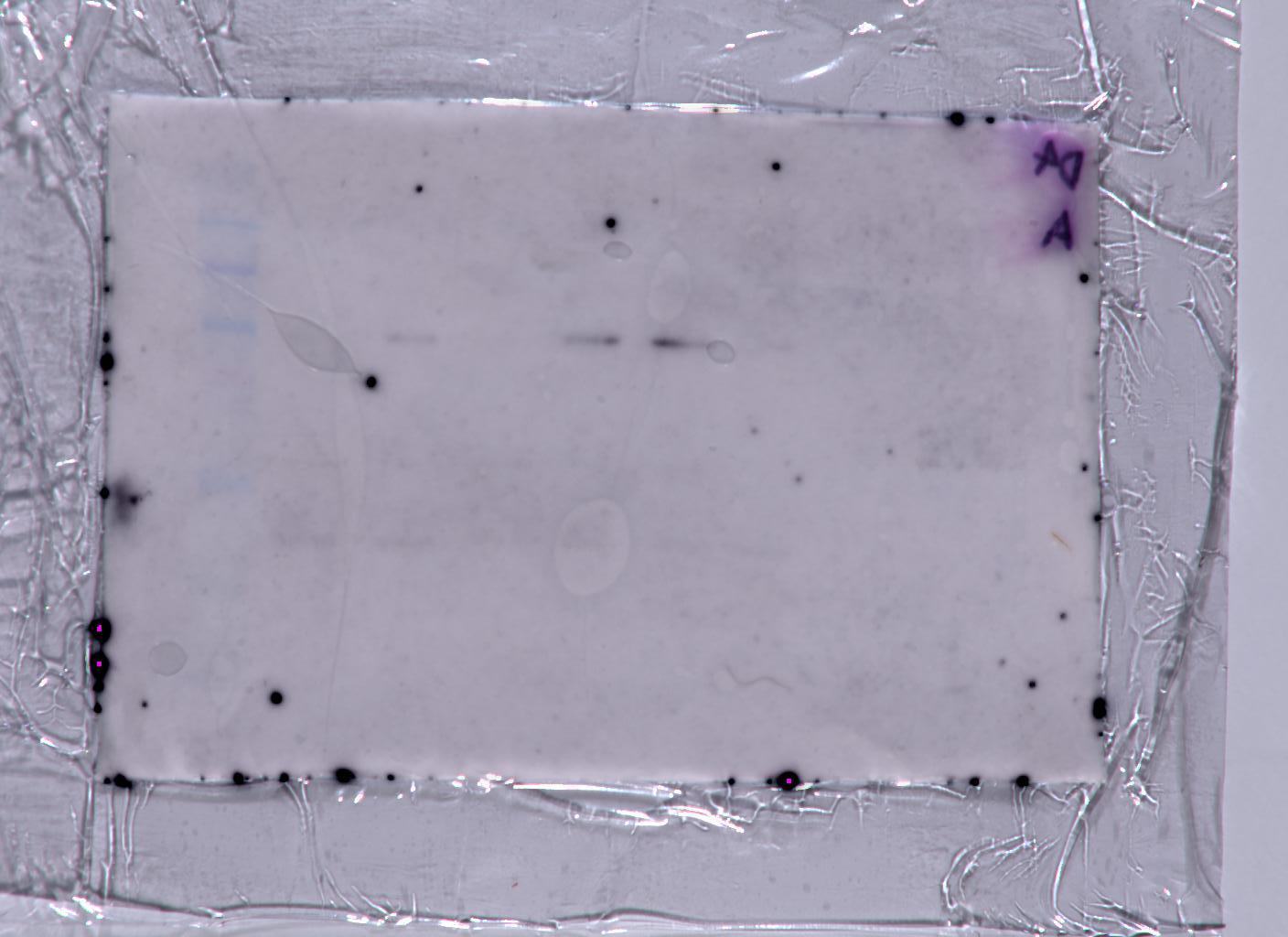

Supplement: Figure 5—figure supplement 1—source data 1. [file elife-69348-fig5-figsupp1-data1.zip › Figure 5ΓÇöfigure supplement 1-source data 1/Figure 5ΓÇöfigure supplement 1-source data 1a/Raw blot/PLOD2 with MW ladder.tif]

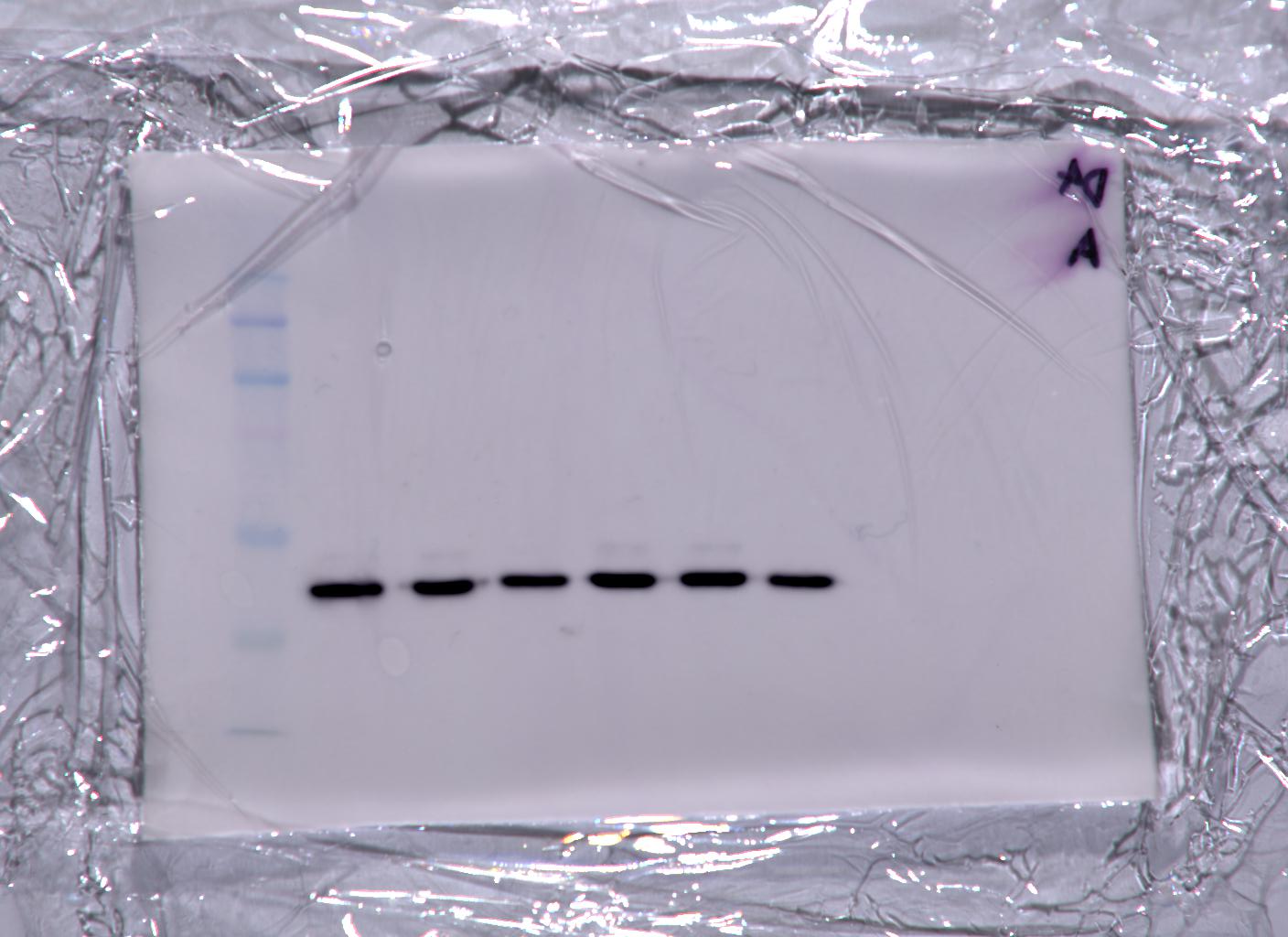

Supplement: Figure 5—figure supplement 1—source data 1. [file elife-69348-fig5-figsupp1-data1.zip › Figure 5ΓÇöfigure supplement 1-source data 1/Figure 5ΓÇöfigure supplement 1-source data 1a/Raw blot/B-actin with MW ladder.tif]

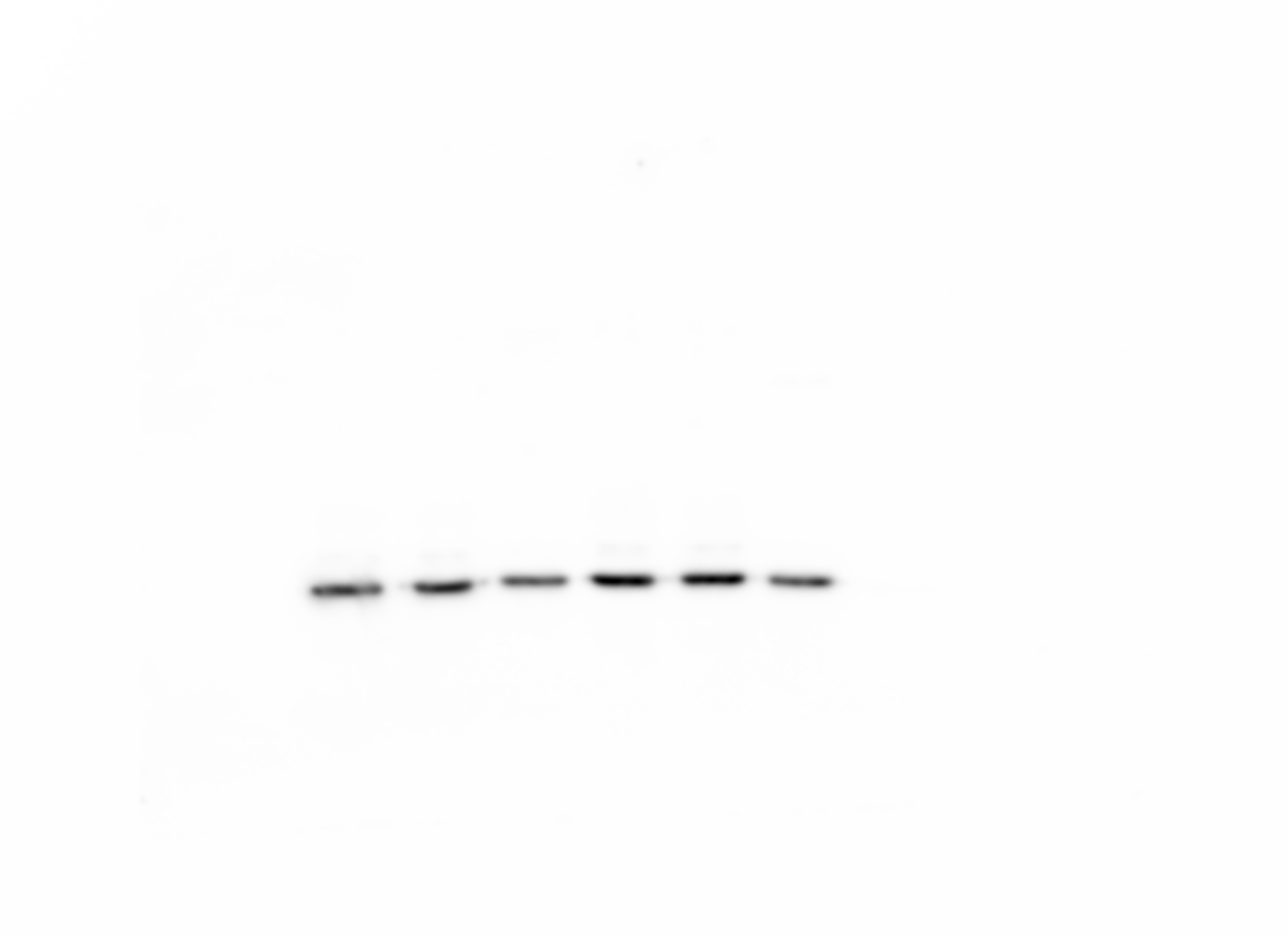

Supplement: Figure 5—figure supplement 1—source data 1. [file elife-69348-fig5-figsupp1-data1.zip › Figure 5ΓÇöfigure supplement 1-source data 1/Figure 5ΓÇöfigure supplement 1-source data 1a/Raw blot/B-actin raw.tif]

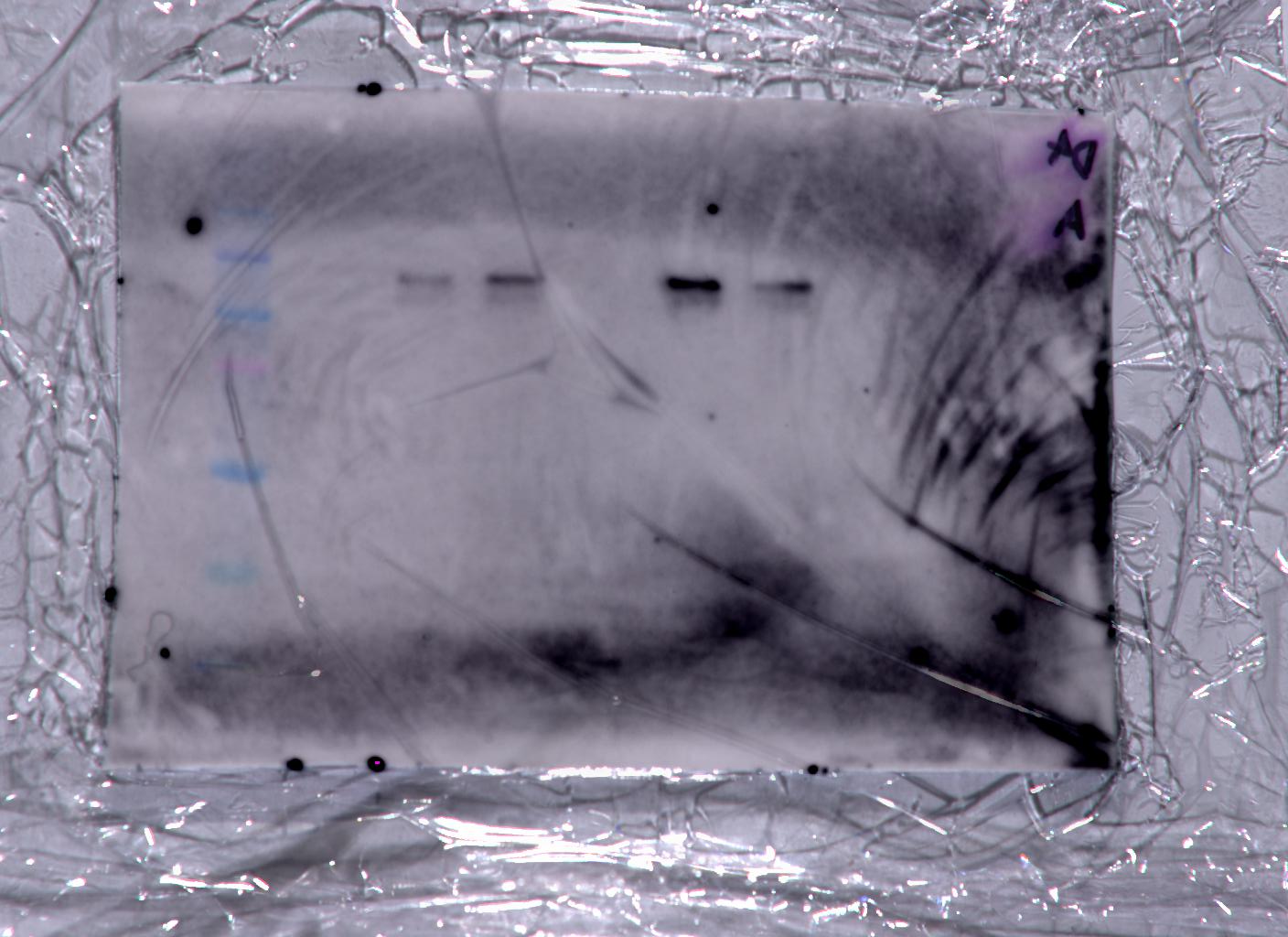

Supplement: Figure 5—figure supplement 1—source data 1. [file elife-69348-fig5-figsupp1-data1.zip › Figure 5ΓÇöfigure supplement 1-source data 1/Figure 5ΓÇöfigure supplement 1-source data 1a/Raw blot/HIF1A with MW ladder.tif]

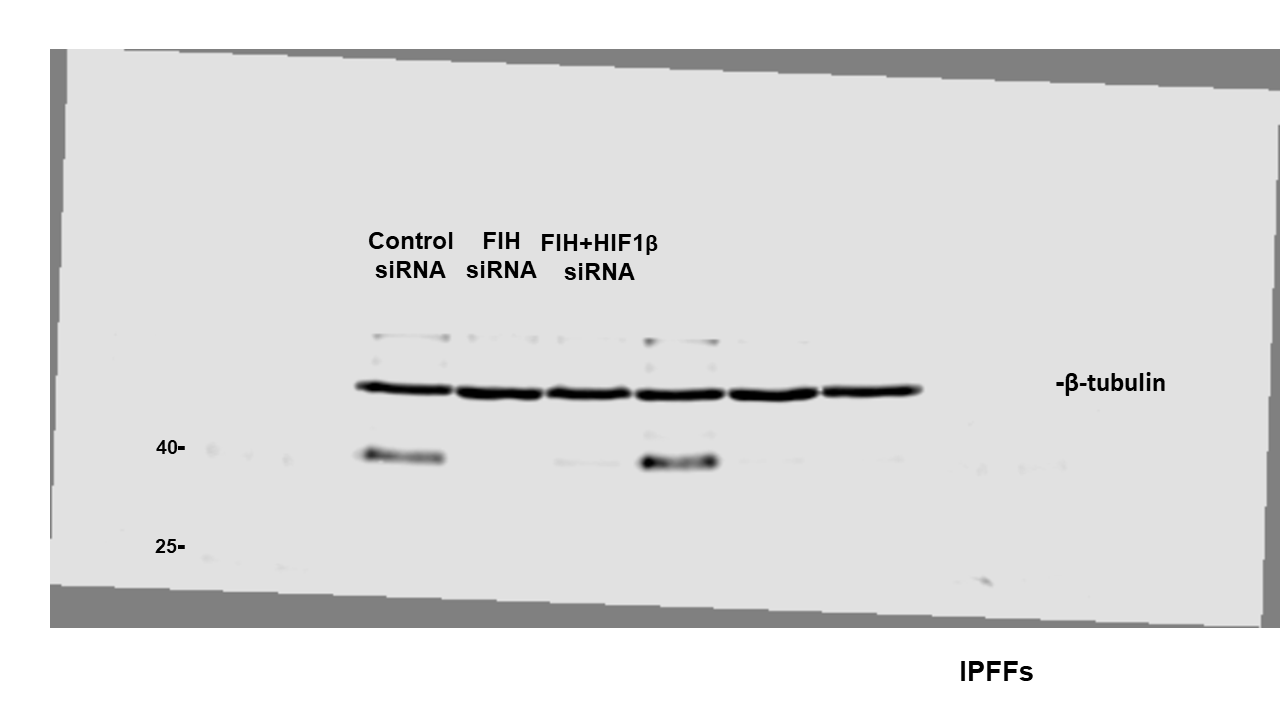

Supplement: Figure 6—source data 1. [file elife-69348-fig6-data1.zip › Figure 6-source data 1/labelled raw blot/BTUBULIN.tif]

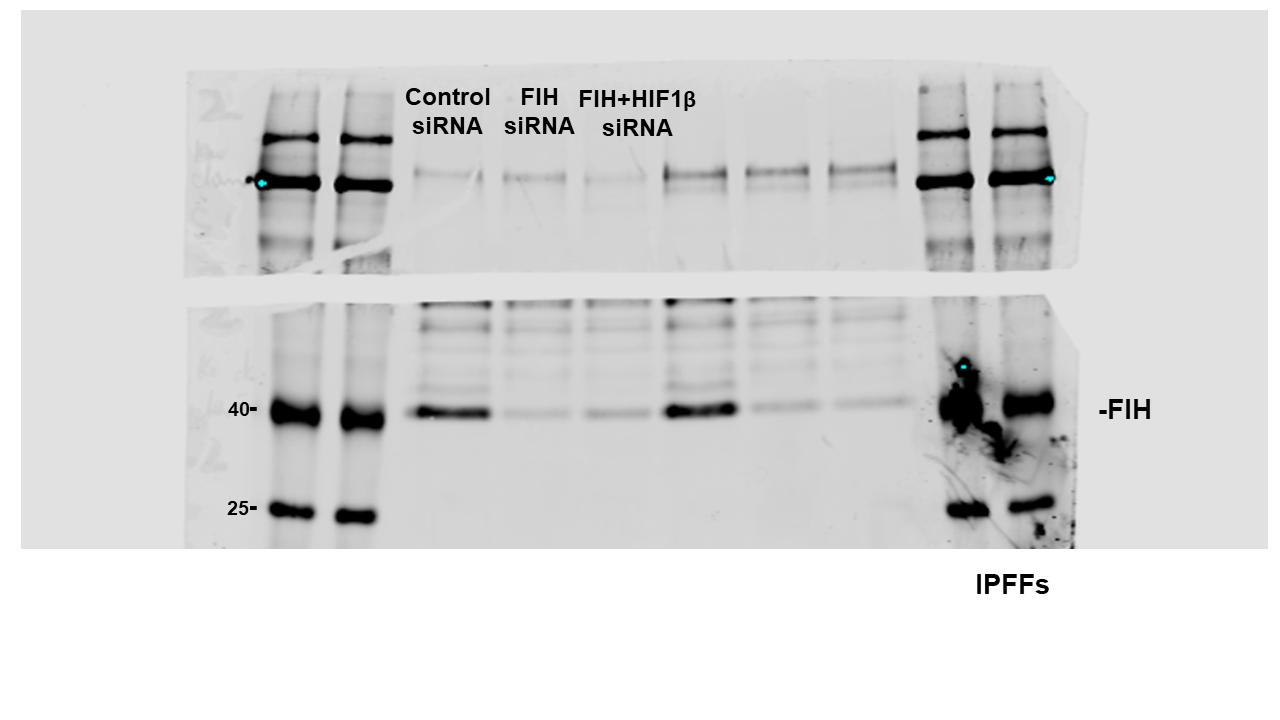

Supplement: Figure 6—source data 1. [file elife-69348-fig6-data1.zip › Figure 6-source data 1/labelled raw blot/FIH.TIF]

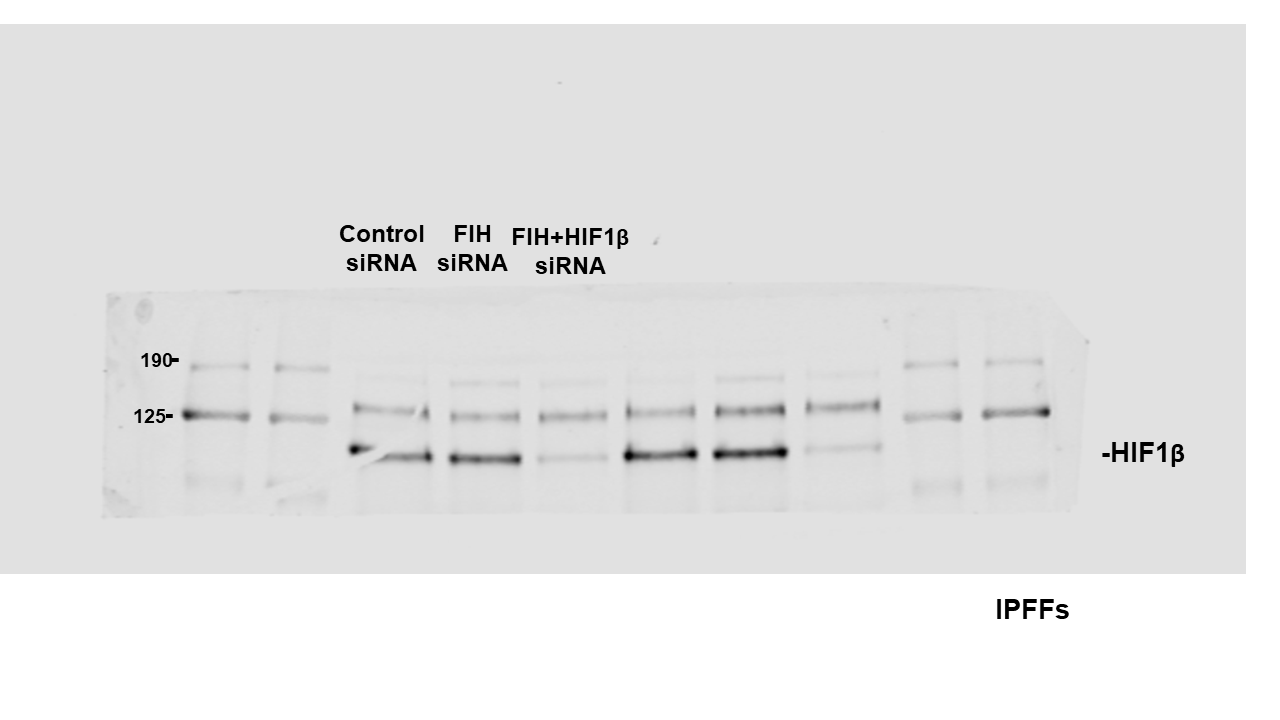

Supplement: Figure 6—source data 1. [file elife-69348-fig6-data1.zip › Figure 6-source data 1/labelled raw blot/HIF1B.TIF]

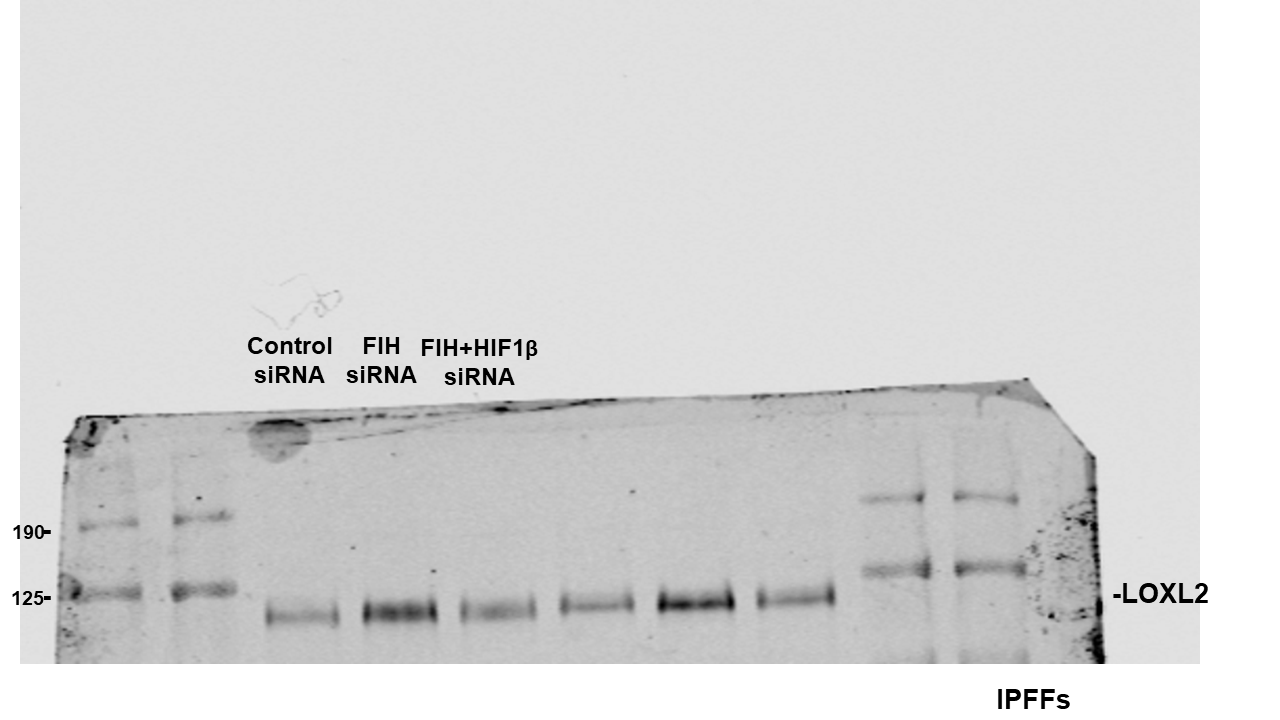

Supplement: Figure 6—source data 1. [file elife-69348-fig6-data1.zip › Figure 6-source data 1/labelled raw blot/LOXL2.TIF]

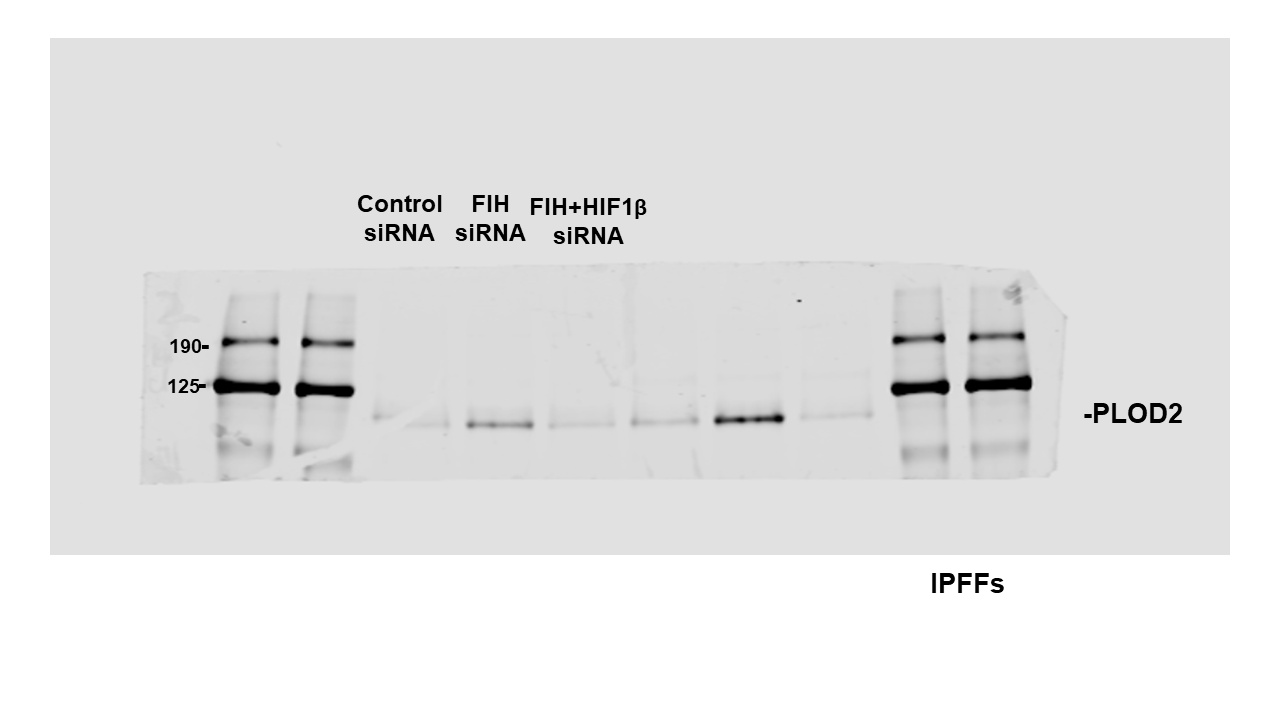

Supplement: Figure 6—source data 1. [file elife-69348-fig6-data1.zip › Figure 6-source data 1/labelled raw blot/PLOD2.TIF]

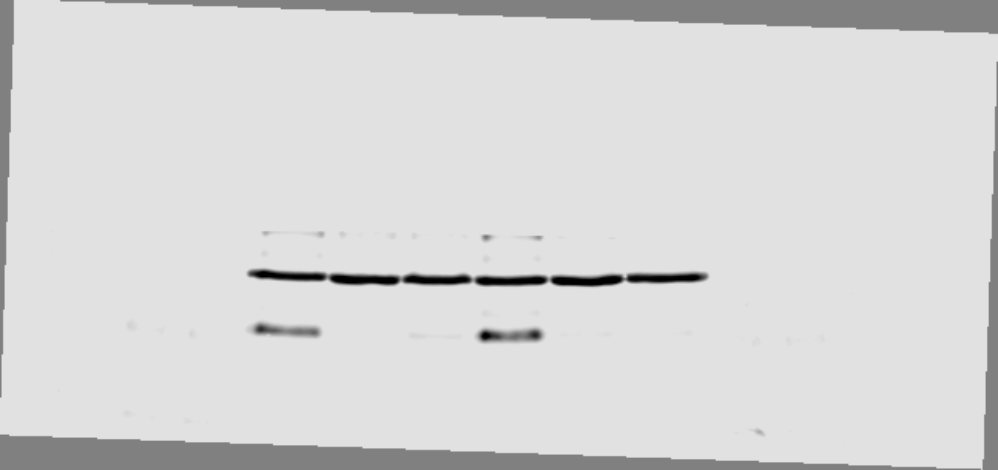

Supplement: Figure 6—source data 1. [file elife-69348-fig6-data1.zip › Figure 6-source data 1/raw blot/BTUBULIN.tif]

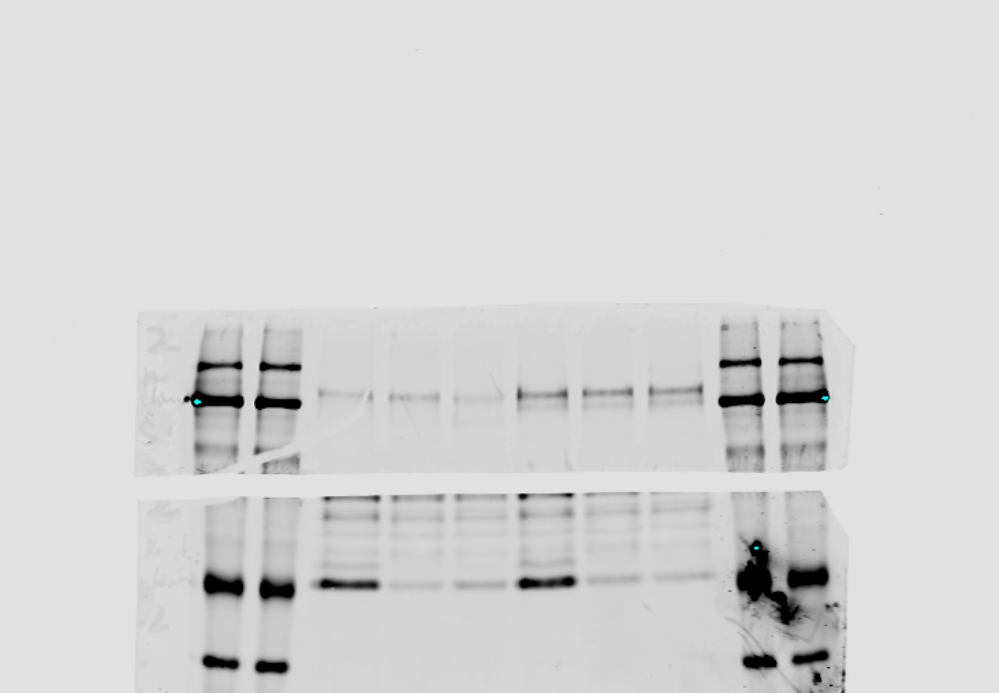

Supplement: Figure 6—source data 1. [file elife-69348-fig6-data1.zip › Figure 6-source data 1/raw blot/FIH.tif]

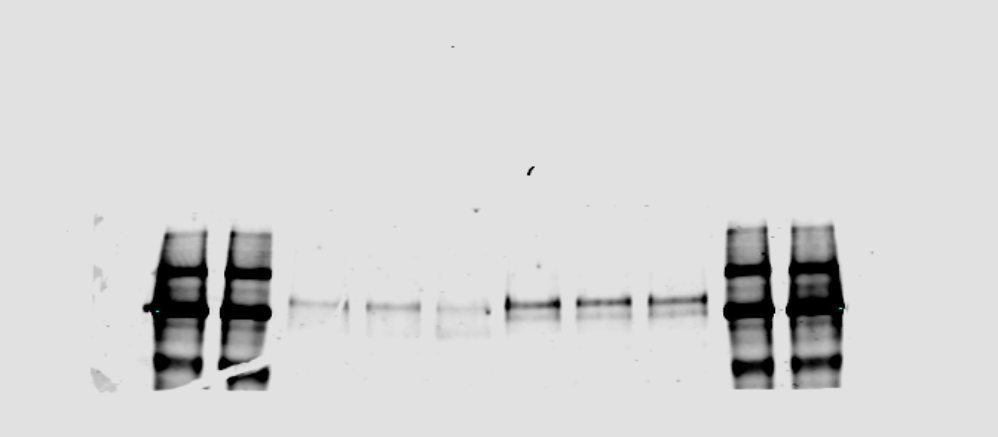

Supplement: Figure 6—source data 1. [file elife-69348-fig6-data1.zip › Figure 6-source data 1/raw blot/HIF1A.tif]

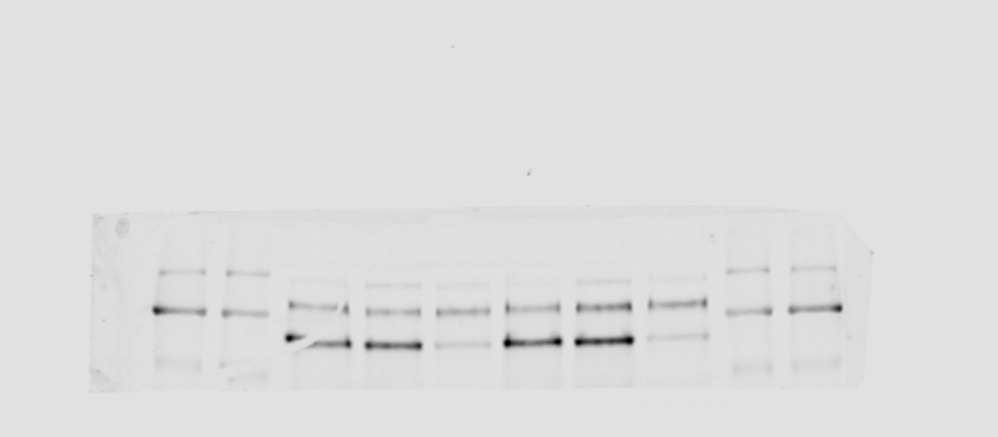

Supplement: Figure 6—source data 1. [file elife-69348-fig6-data1.zip › Figure 6-source data 1/raw blot/HIF1B.tif]

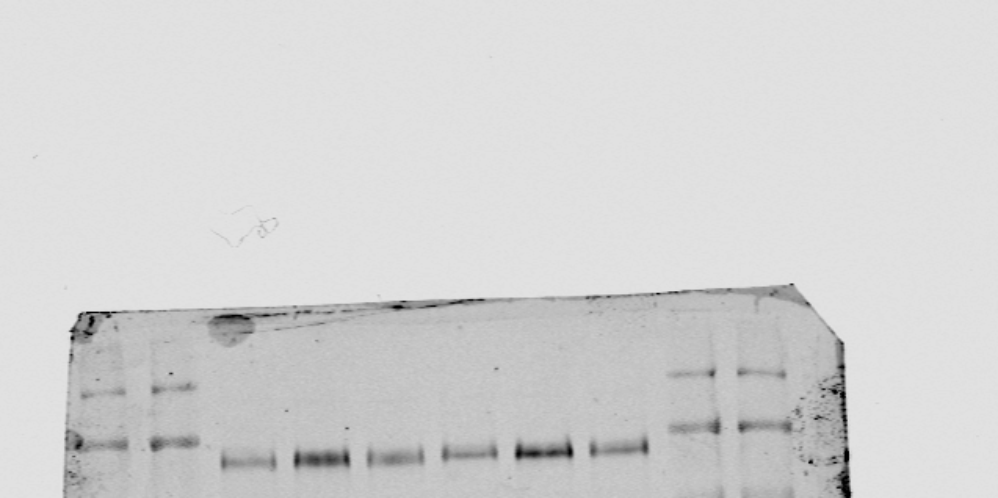

Supplement: Figure 6—source data 1. [file elife-69348-fig6-data1.zip › Figure 6-source data 1/raw blot/LOXL2.tif]

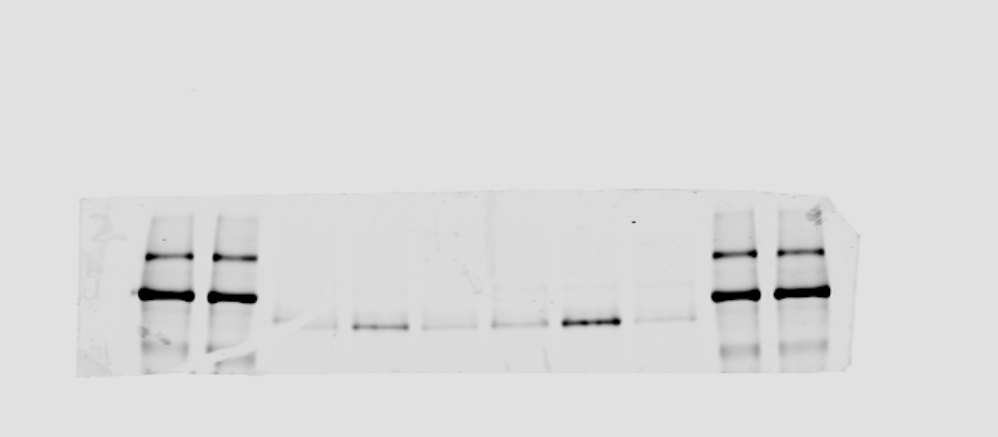

Supplement: Figure 6—source data 1. [file elife-69348-fig6-data1.zip › Figure 6-source data 1/raw blot/PLOD2.tif]
