# Supplementary material for: Pseudohypoxic HIF pathway activation dysregulates collagen structure-function in human lung fibrosis
Source: eLife. 2022 Feb 21;11:e69348. doi: 10.7554/eLife.69348 (PMC8860444; doi:10.7554/eLife.69348)
Supplement: Figure 2—figure supplement 1—source data 1. [file elife-69348-fig2-figsupp1-data1.zip › FIgure2-figure supplement 1-source data 1/FIgure2-figure supplement 1-source data 1a/FIgure2-figure supplement 1-source data 1a labelled.pptx]

## Slide 1
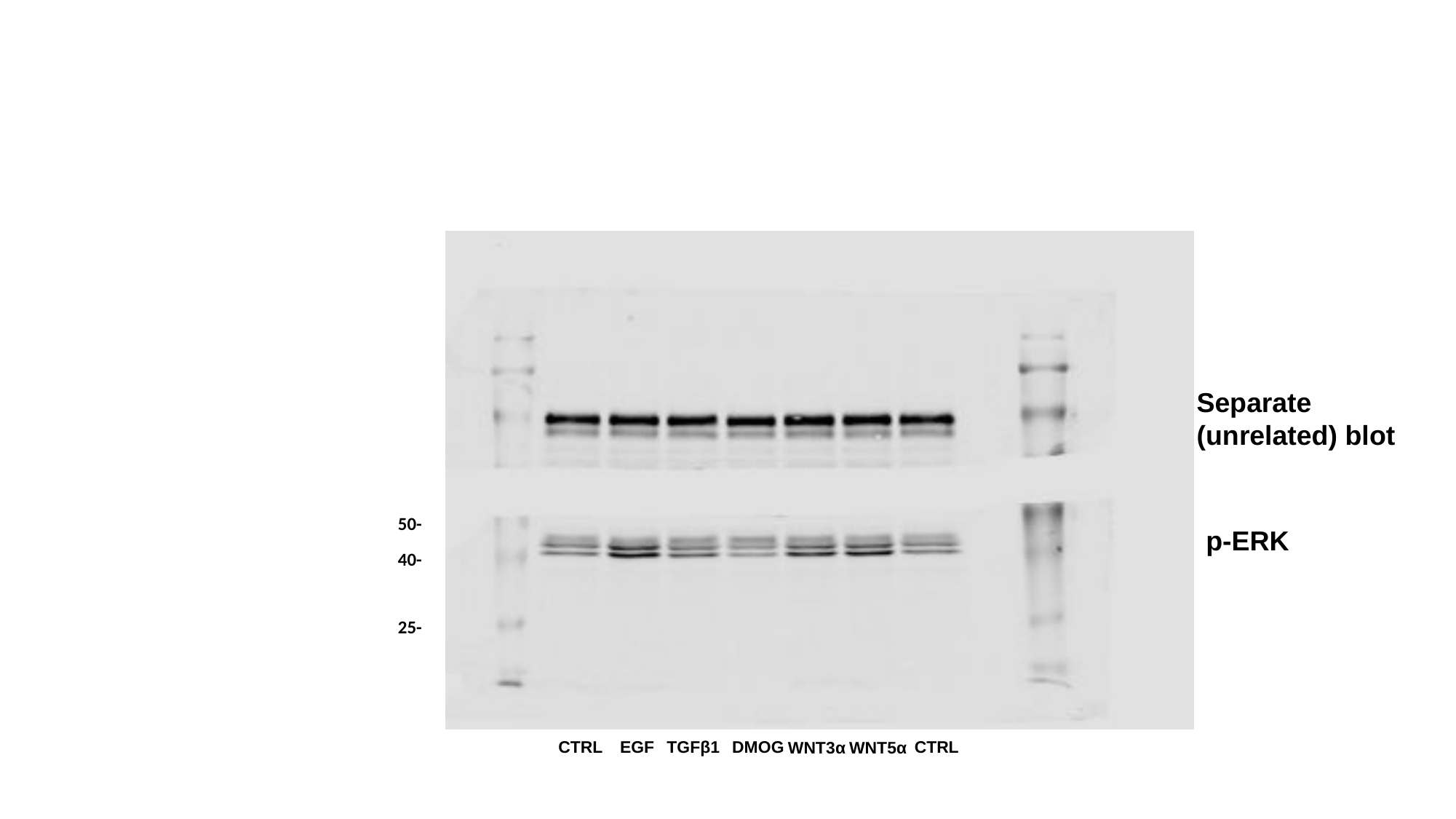

#
Separate (unrelated) blot
50-
p-ERK
40-
25-
CTRL
EGF
TGFβ1
DMOG
CTRL
WNT3α
WNT5α

## Slide 2
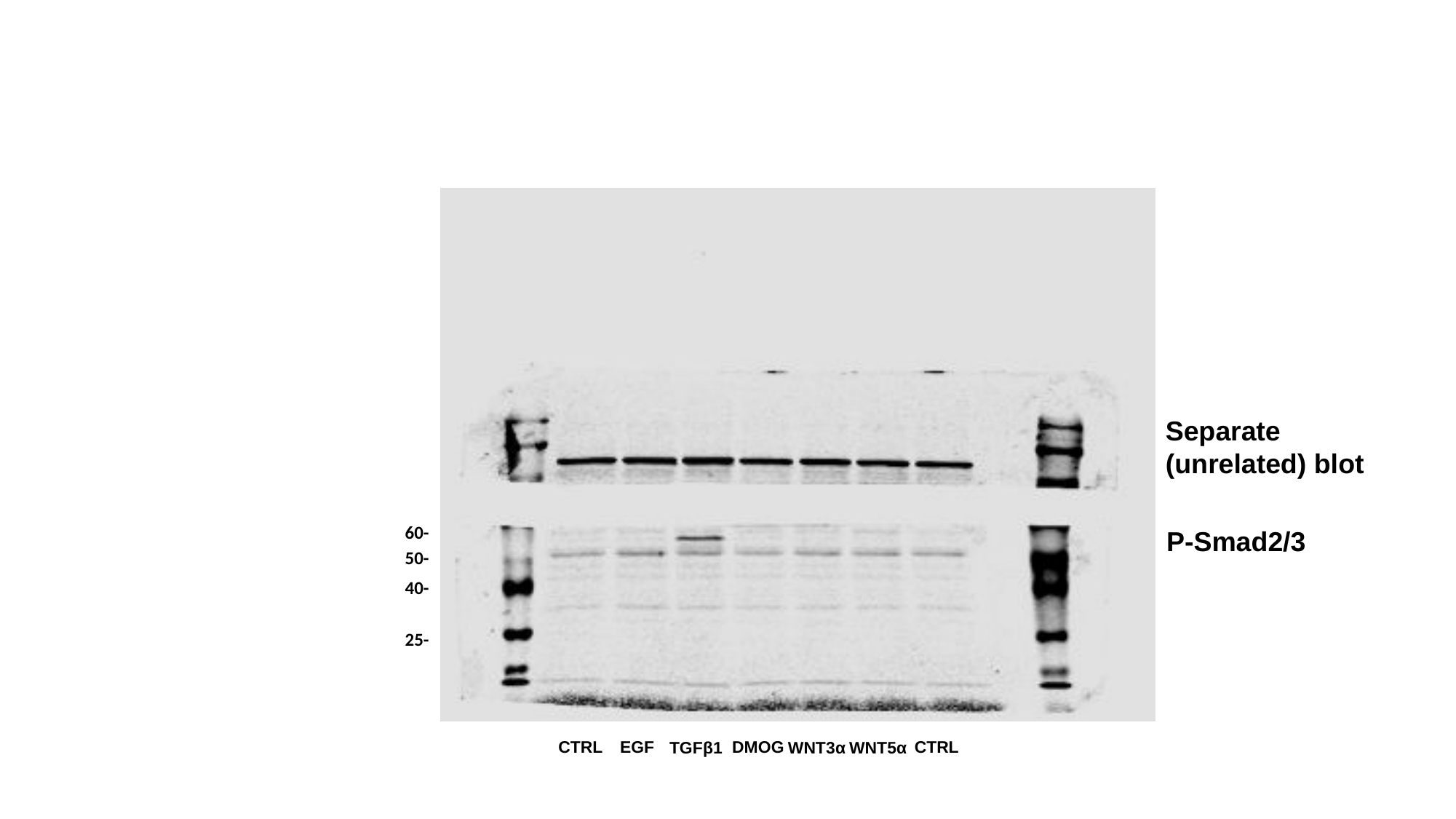

#
Separate (unrelated) blot
60-
P-Smad2/3
50-
40-
25-
CTRL
EGF
DMOG
CTRL
TGFβ1
WNT3α
WNT5α

## Slide 3
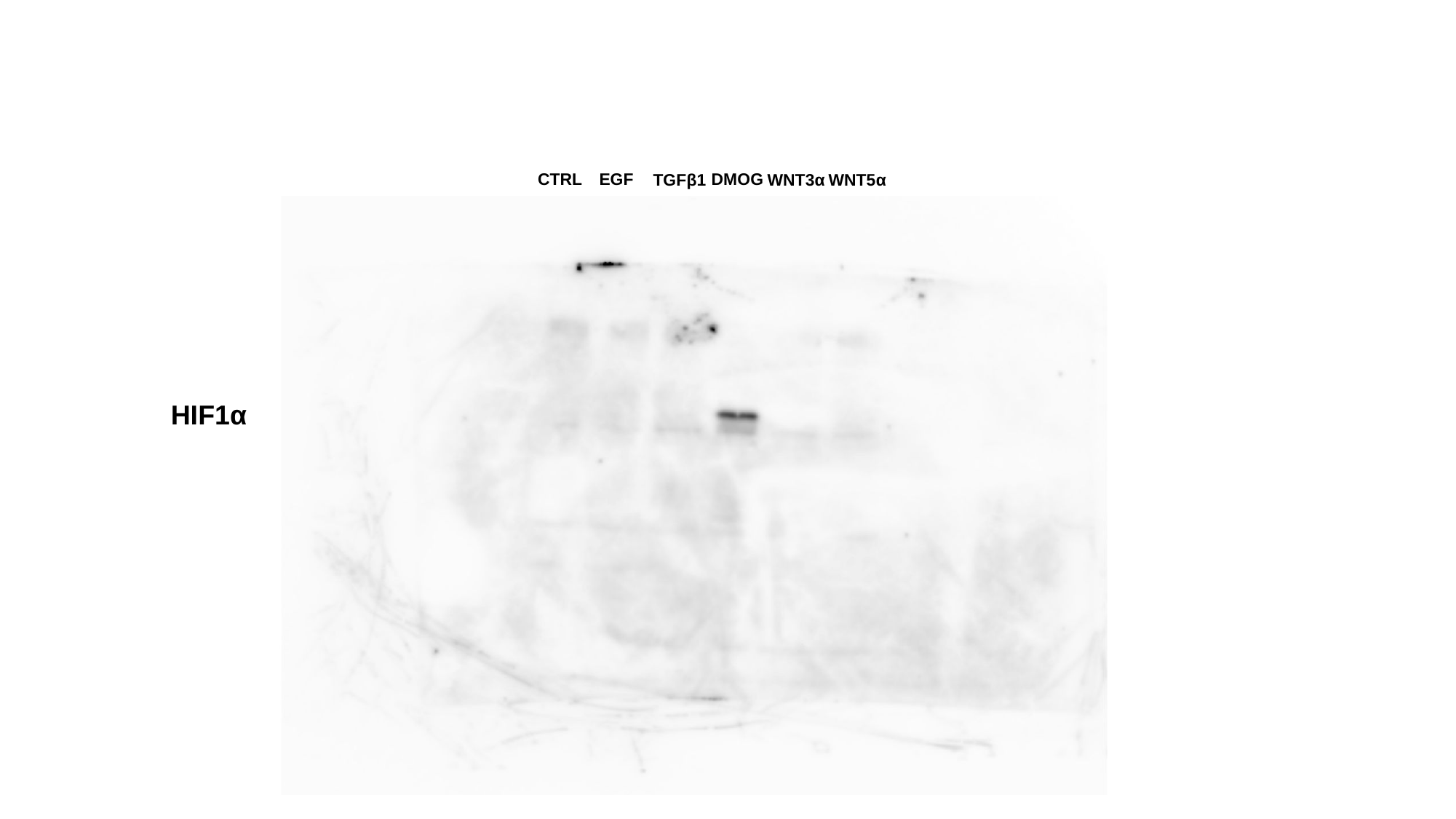

CTRL
EGF
DMOG
TGFβ1
WNT3α
WNT5α
HIF1α

## Slide 4
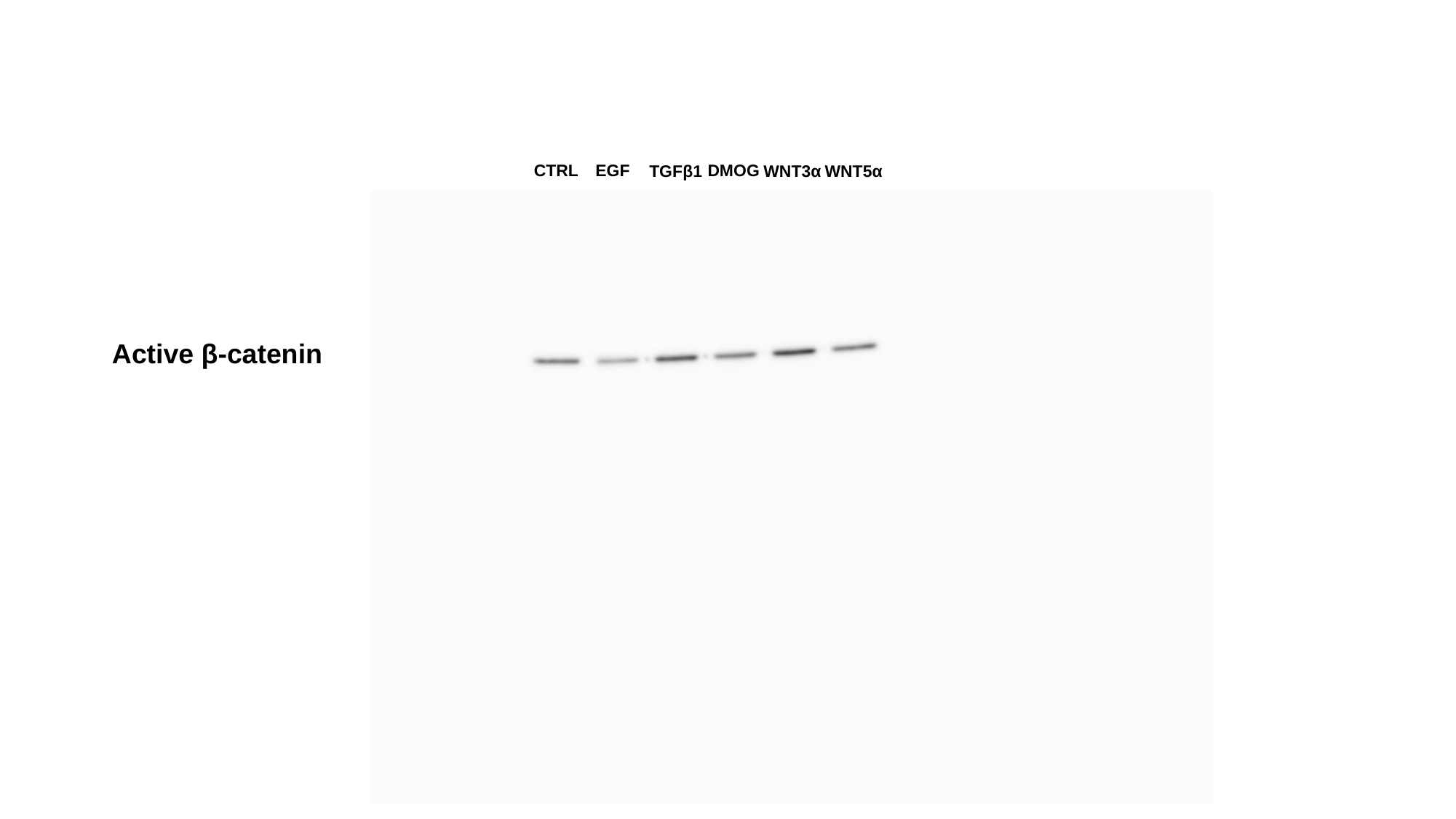

#
CTRL
EGF
DMOG
TGFβ1
WNT3α
WNT5α
Active β-catenin

## Slide 5
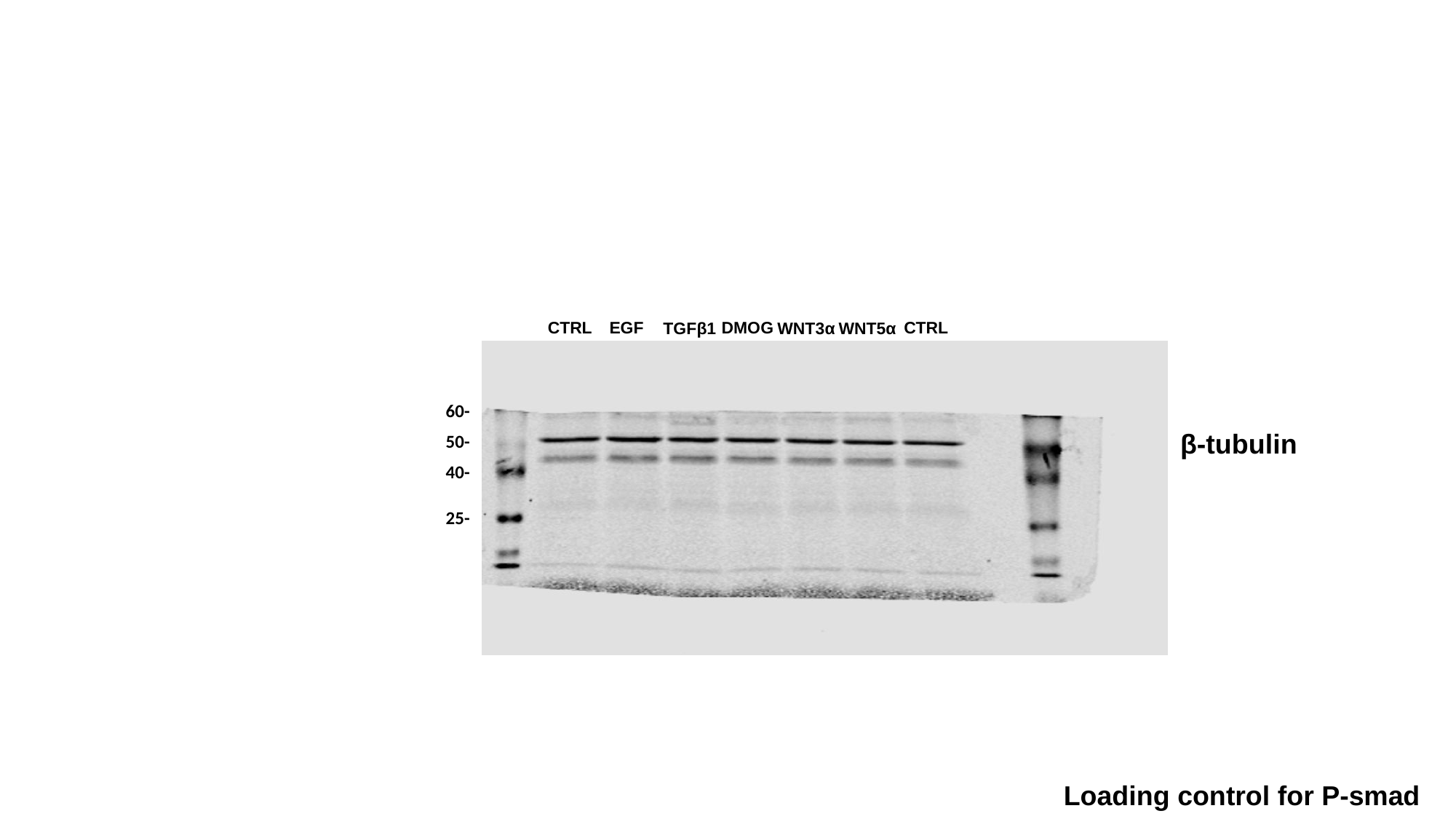

#
CTRL
EGF
DMOG
CTRL
TGFβ1
WNT3α
WNT5α
60-
β-tubulin
50-
40-
25-
Loading control for P-smad
