# Supplementary material for: Pseudohypoxic HIF pathway activation dysregulates collagen structure-function in human lung fibrosis
Source: eLife. 2022 Feb 21;11:e69348. doi: 10.7554/eLife.69348 (PMC8860444; doi:10.7554/eLife.69348)
Supplement: Figure 2—figure supplement 1—source data 1. [file elife-69348-fig2-figsupp1-data1.zip › FIgure2-figure supplement 1-source data 1/FIgure2-figure supplement 1-source data 1d/FIgure2-figure supplement 1-source data 1d labelled.pptx]

## Slide 1
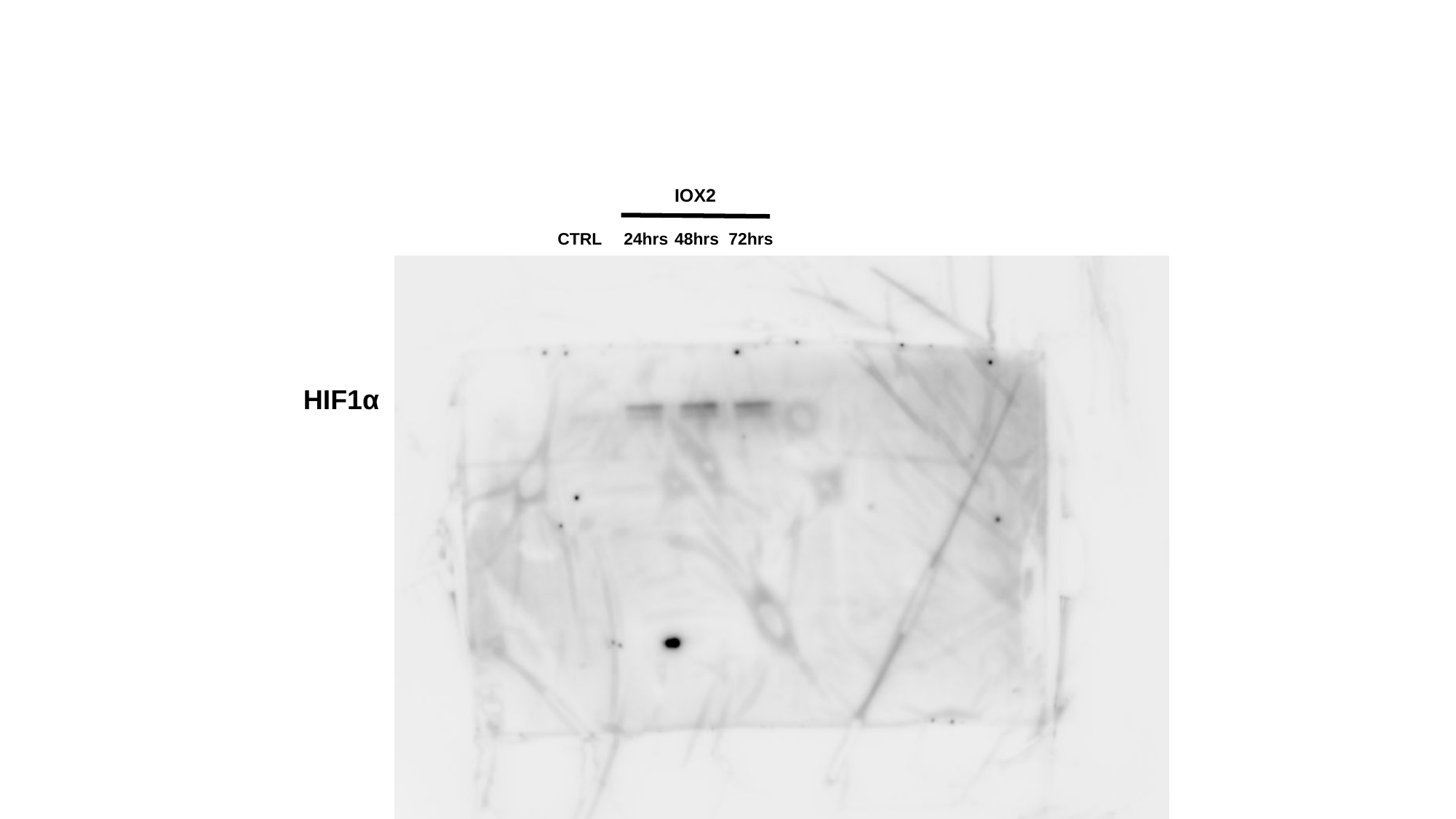

IOX2
CTRL
24hrs
48hrs
72hrs
HIF1α

## Slide 2
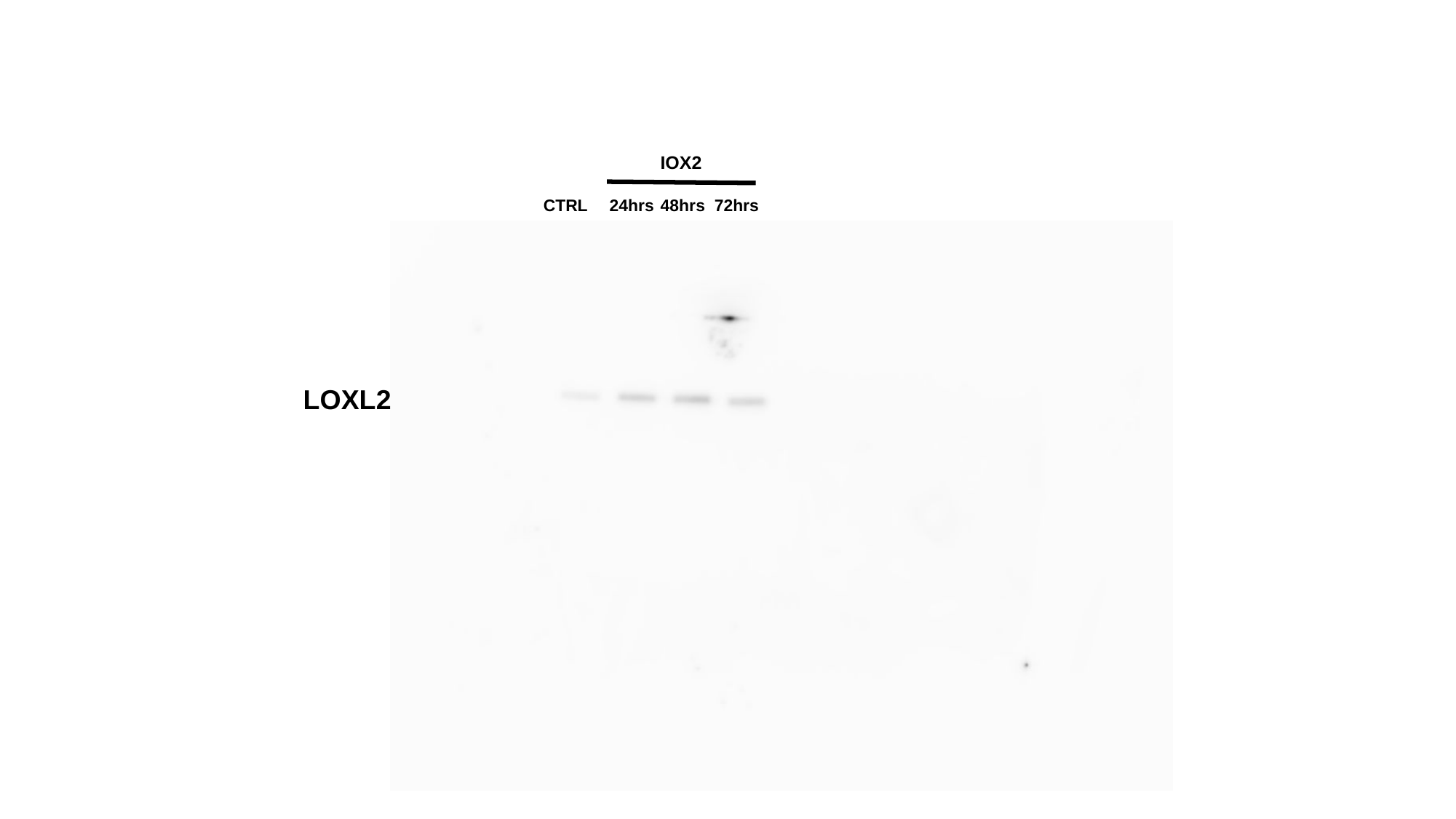

IOX2
CTRL
24hrs
48hrs
72hrs
LOXL2

## Slide 3
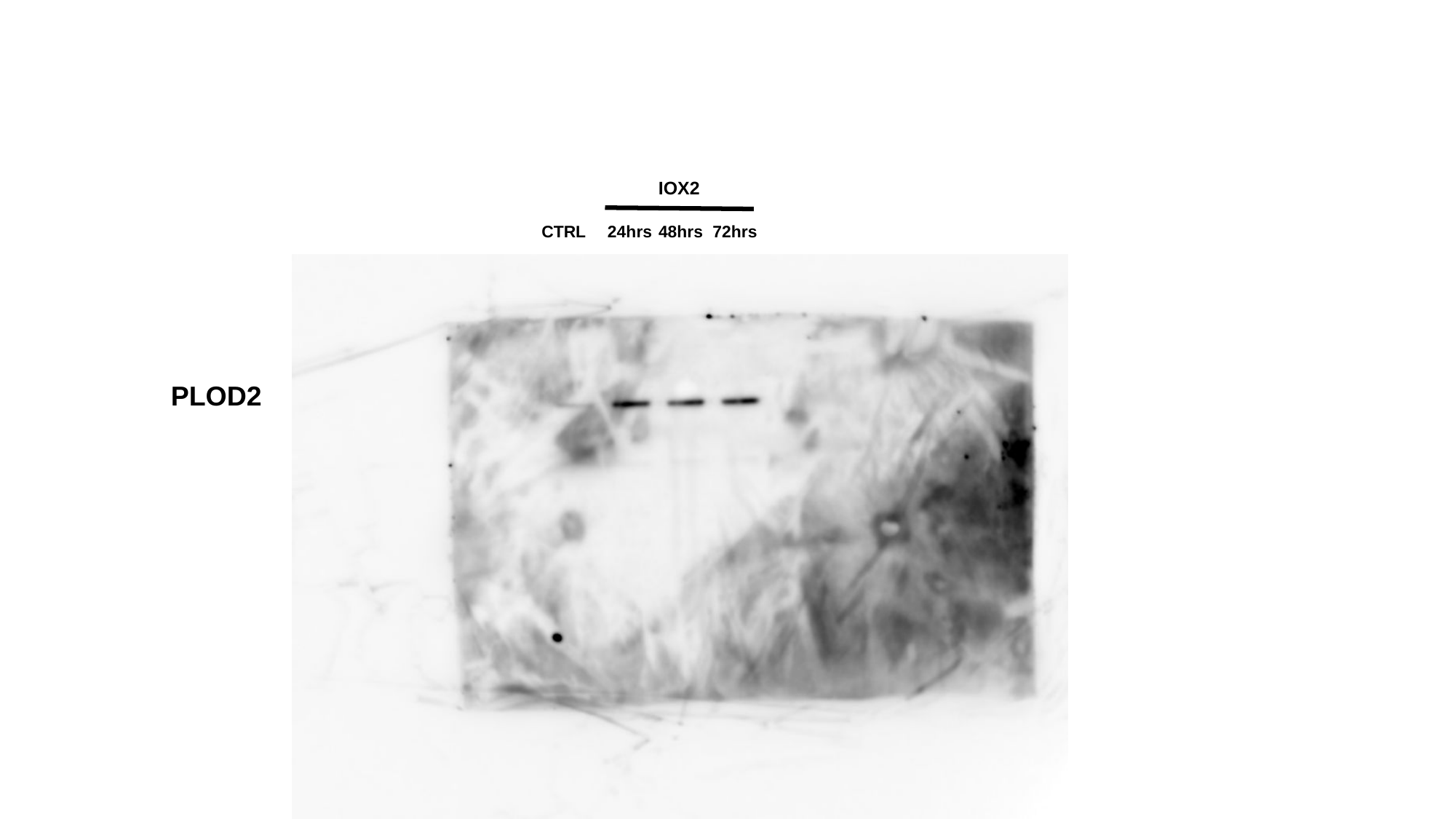

IOX2
CTRL
24hrs
48hrs
72hrs
PLOD2

## Slide 4
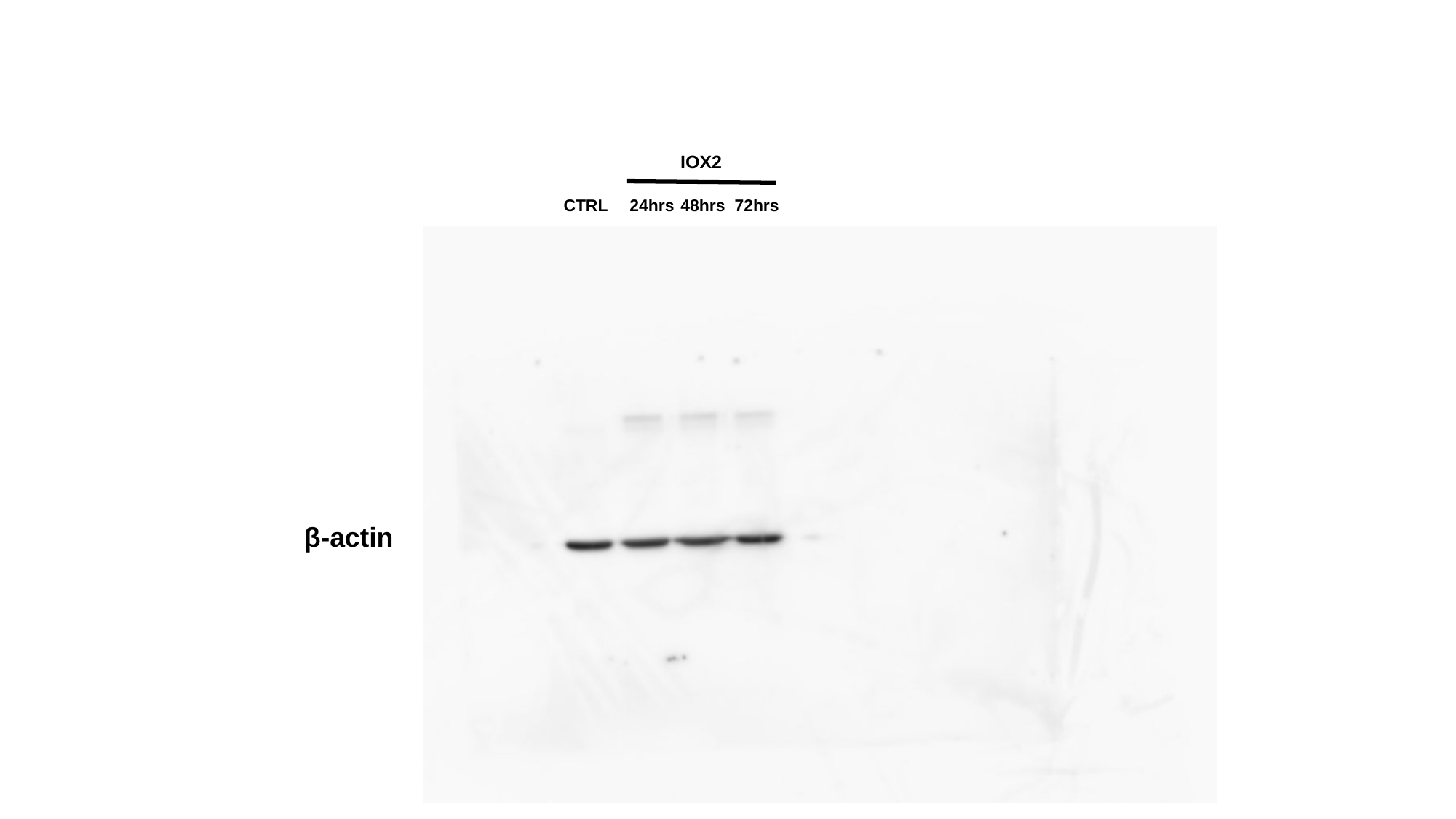

IOX2
CTRL
24hrs
48hrs
72hrs
β-actin
