# Supplementary material for: Pseudohypoxic HIF pathway activation dysregulates collagen structure-function in human lung fibrosis
Source: eLife. 2022 Feb 21;11:e69348. doi: 10.7554/eLife.69348 (PMC8860444; doi:10.7554/eLife.69348)
Supplement: Figure 3—source data 1. [file elife-69348-fig3-data1.zip › Figure 3-source data 1/Figure 3d labelled.pptx]

## Slide 1
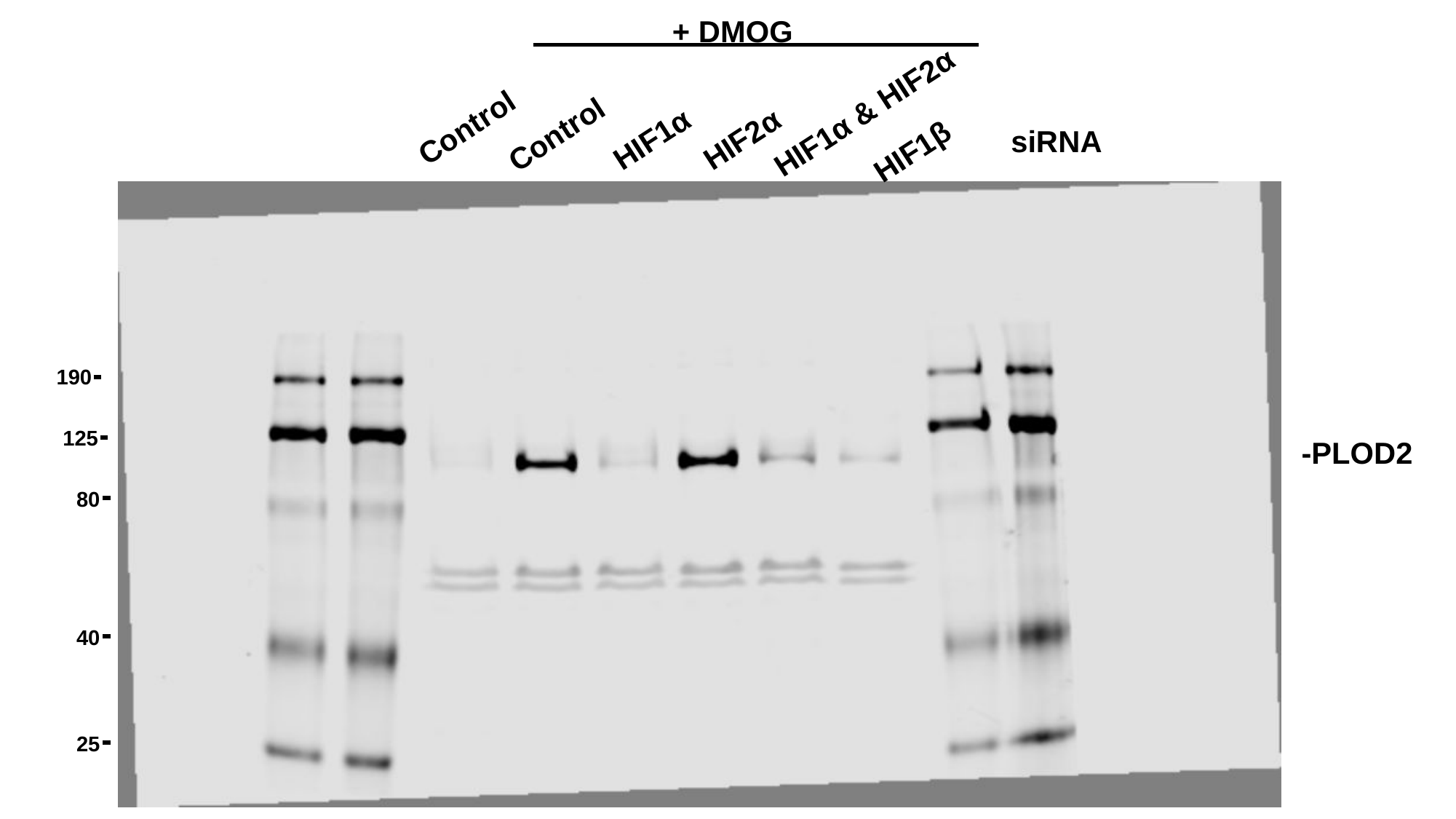

+ DMOG
 HIF1α & HIF2α
 Control
 Control
 HIF1α
 HIF2α
 HIF1β
siRNA
190
125
-PLOD2
80
40
25

## Slide 2
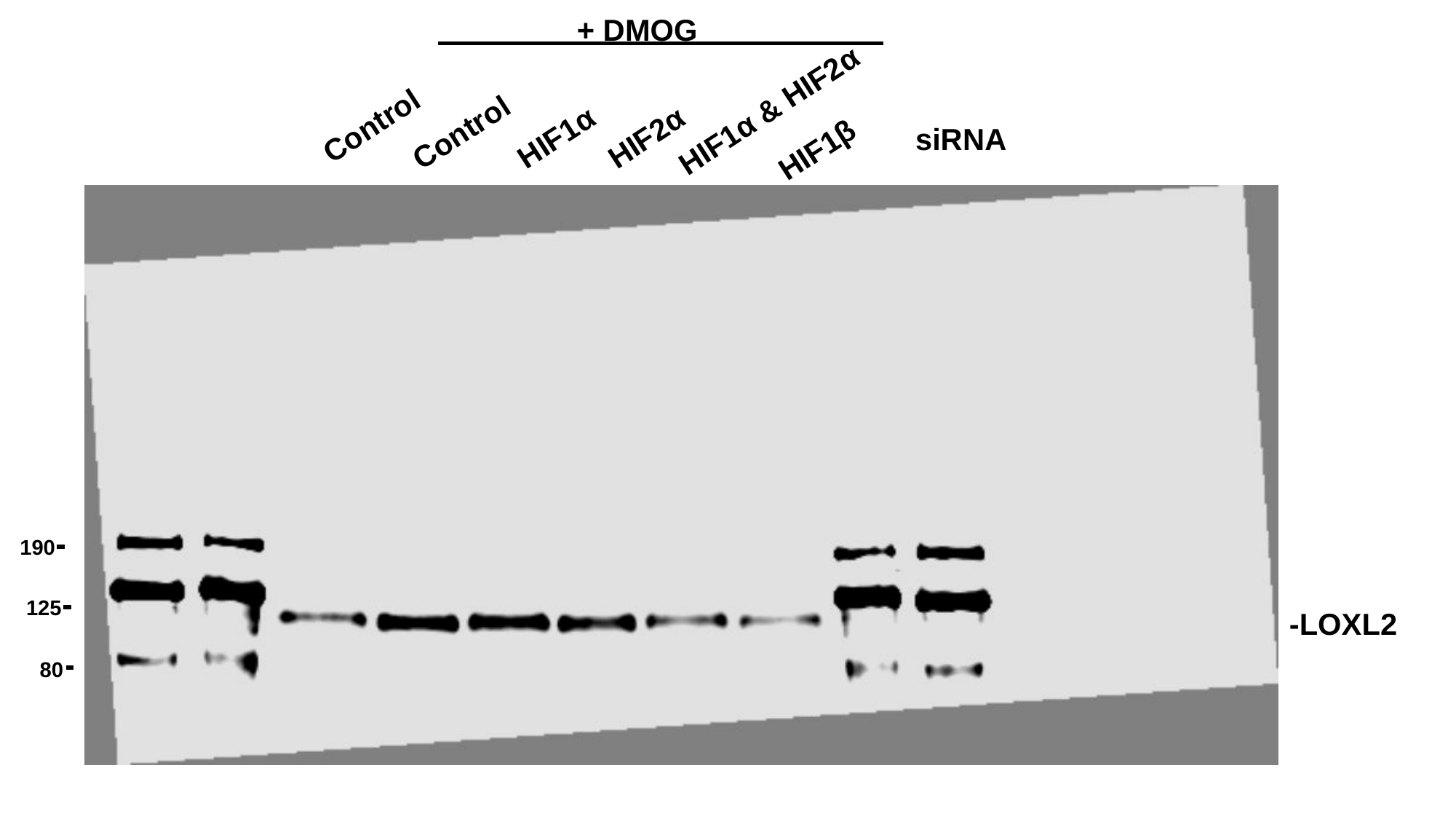

+ DMOG
 HIF1α & HIF2α
 Control
 Control
 HIF1α
 HIF2α
 HIF1β
siRNA
190
125
-LOXL2
80

## Slide 3
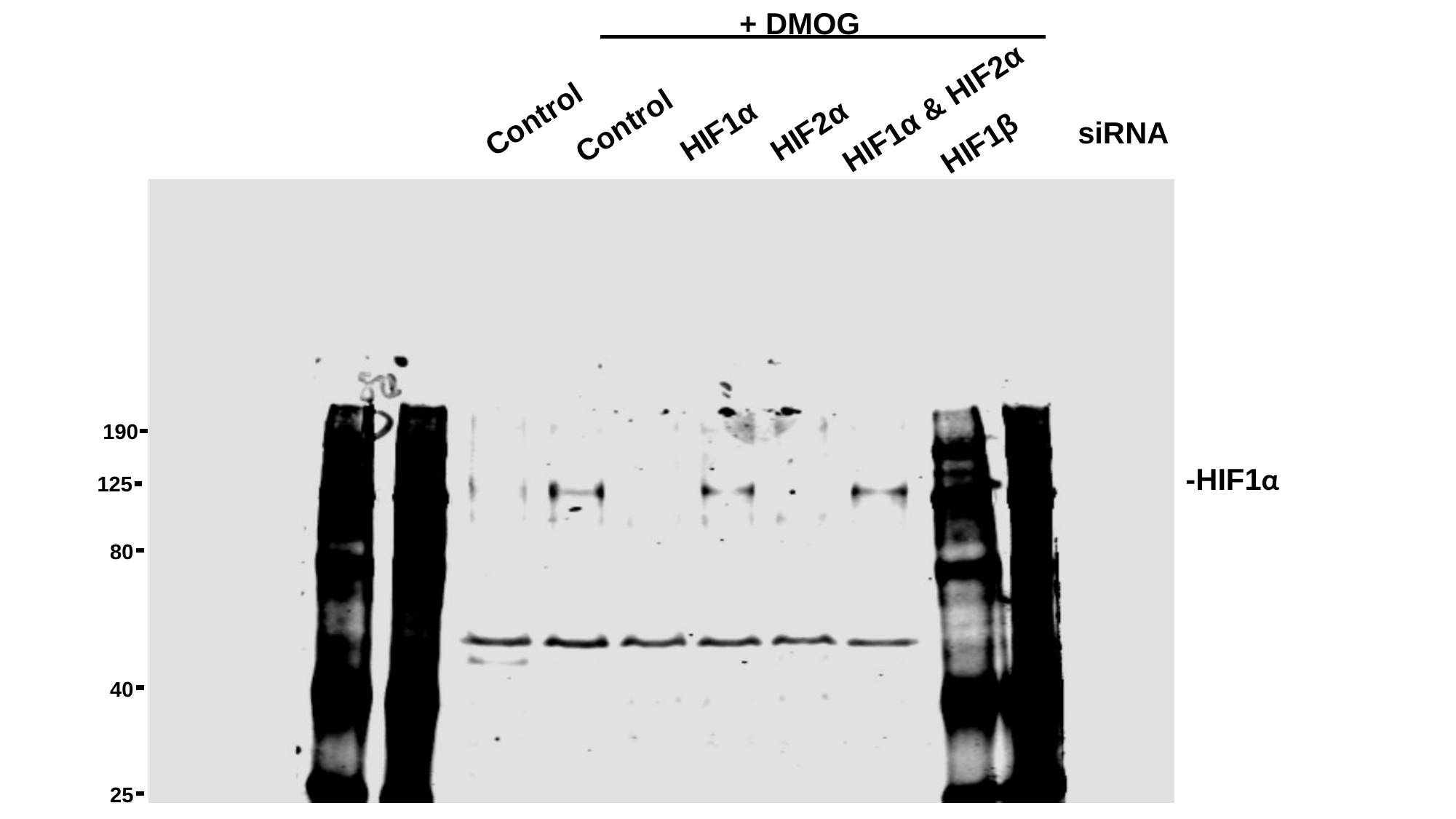

+ DMOG
 HIF1α & HIF2α
 Control
 Control
 HIF1α
 HIF2α
 HIF1β
siRNA
190
-HIF1α
125
80
40
25

## Slide 4
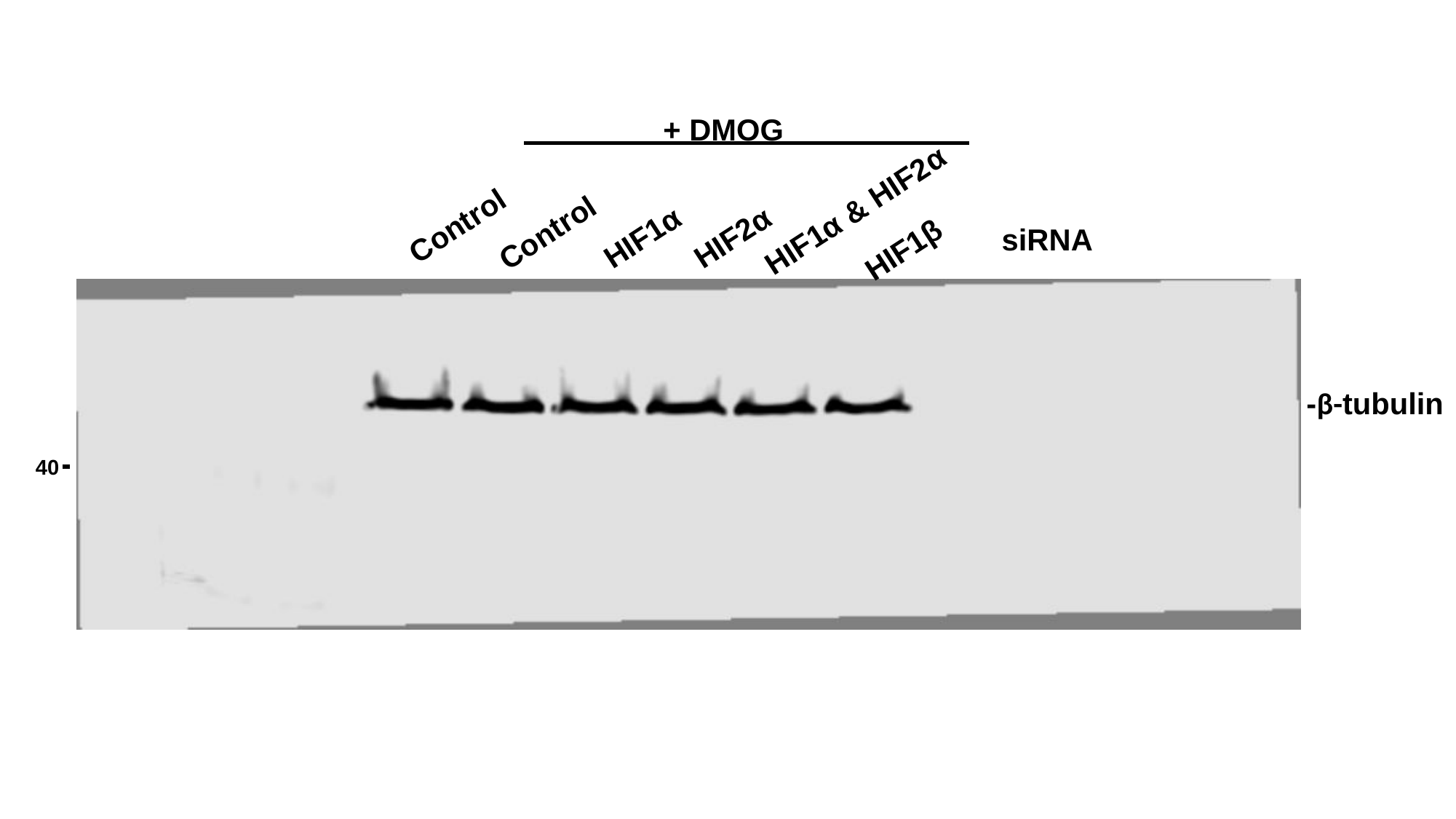

+ DMOG
 HIF1α & HIF2α
 Control
 Control
 HIF1α
 HIF2α
 HIF1β
siRNA
-β-tubulin
40
