# Supplementary material for: Pseudohypoxic HIF pathway activation dysregulates collagen structure-function in human lung fibrosis
Source: eLife. 2022 Feb 21;11:e69348. doi: 10.7554/eLife.69348 (PMC8860444; doi:10.7554/eLife.69348)
Supplement: Figure 4—source data 1. [file elife-69348-fig4-data1.zip › Figure 4-source data 1/Figure 4C/Figure 4C labelled.pptx]

## Slide 1
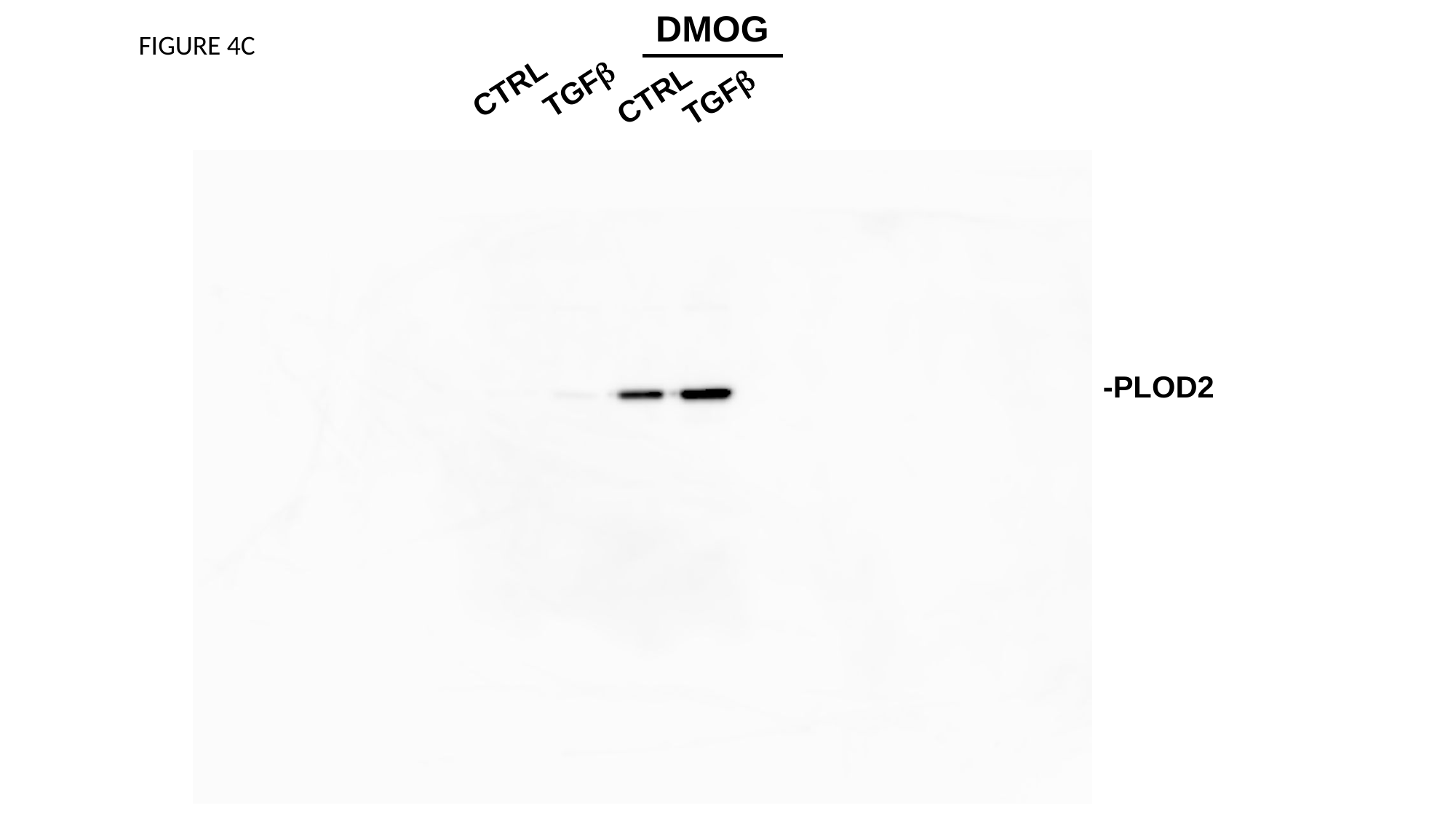

DMOG
FIGURE 4C
 TGFb
 CTRL
 TGFb
 CTRL
-PLOD2

## Slide 2
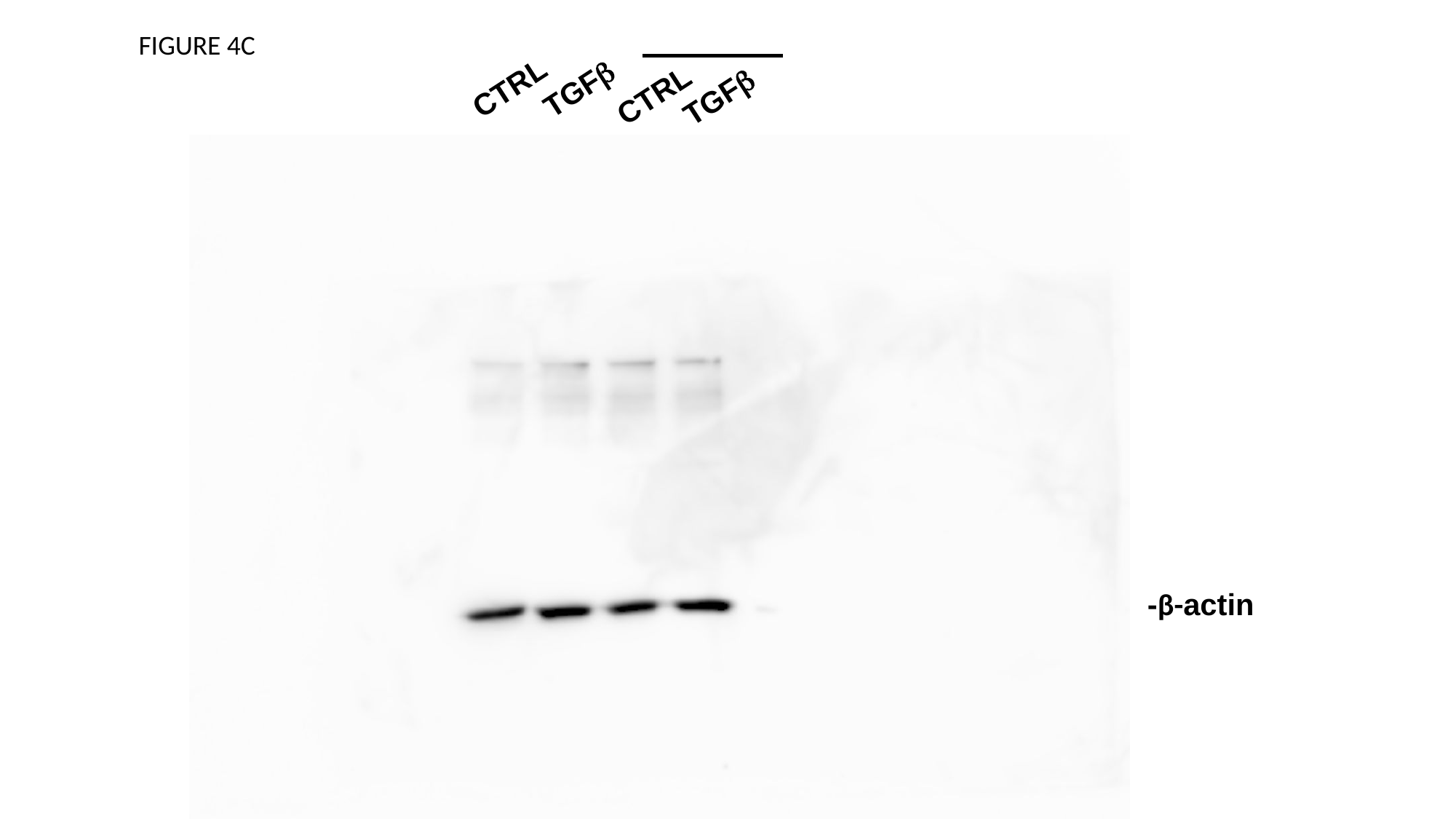

FIGURE 4C
 TGFb
 CTRL
 TGFb
 CTRL
-β-actin

## Slide 3
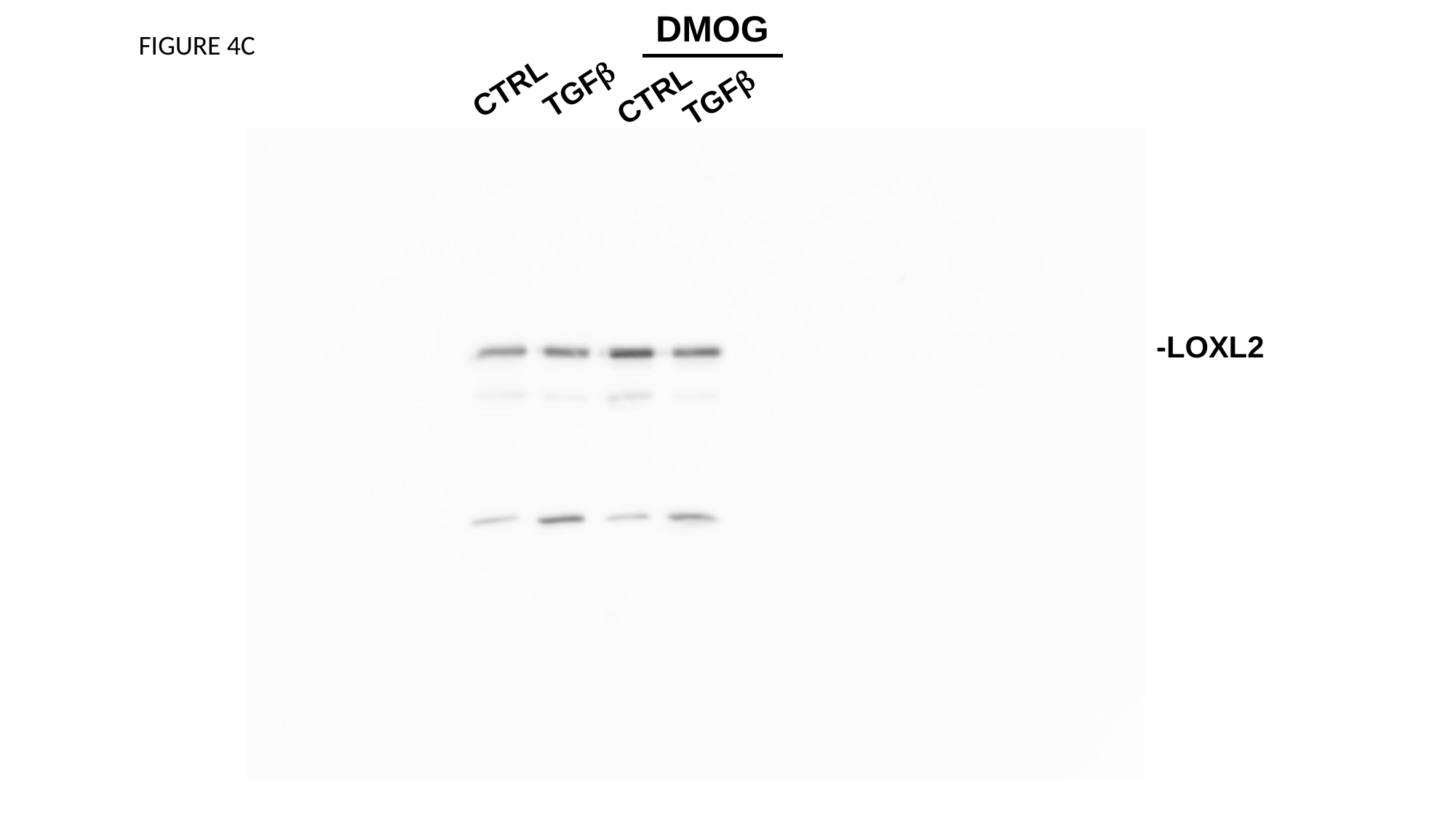

DMOG
FIGURE 4C
 TGFb
 CTRL
 TGFb
 CTRL
-LOXL2
