# Supplementary material for: Pseudohypoxic HIF pathway activation dysregulates collagen structure-function in human lung fibrosis
Source: eLife. 2022 Feb 21;11:e69348. doi: 10.7554/eLife.69348 (PMC8860444; doi:10.7554/eLife.69348)
Supplement: Figure 4—figure supplement 1—source data 1. [file elife-69348-fig4-figsupp1-data1.zip › Figure 4-figure supplement 1ΓÇösource data 1/Figure 4-figure supplement 1ΓÇösource data 1 labelled.pptx]

## Slide 1
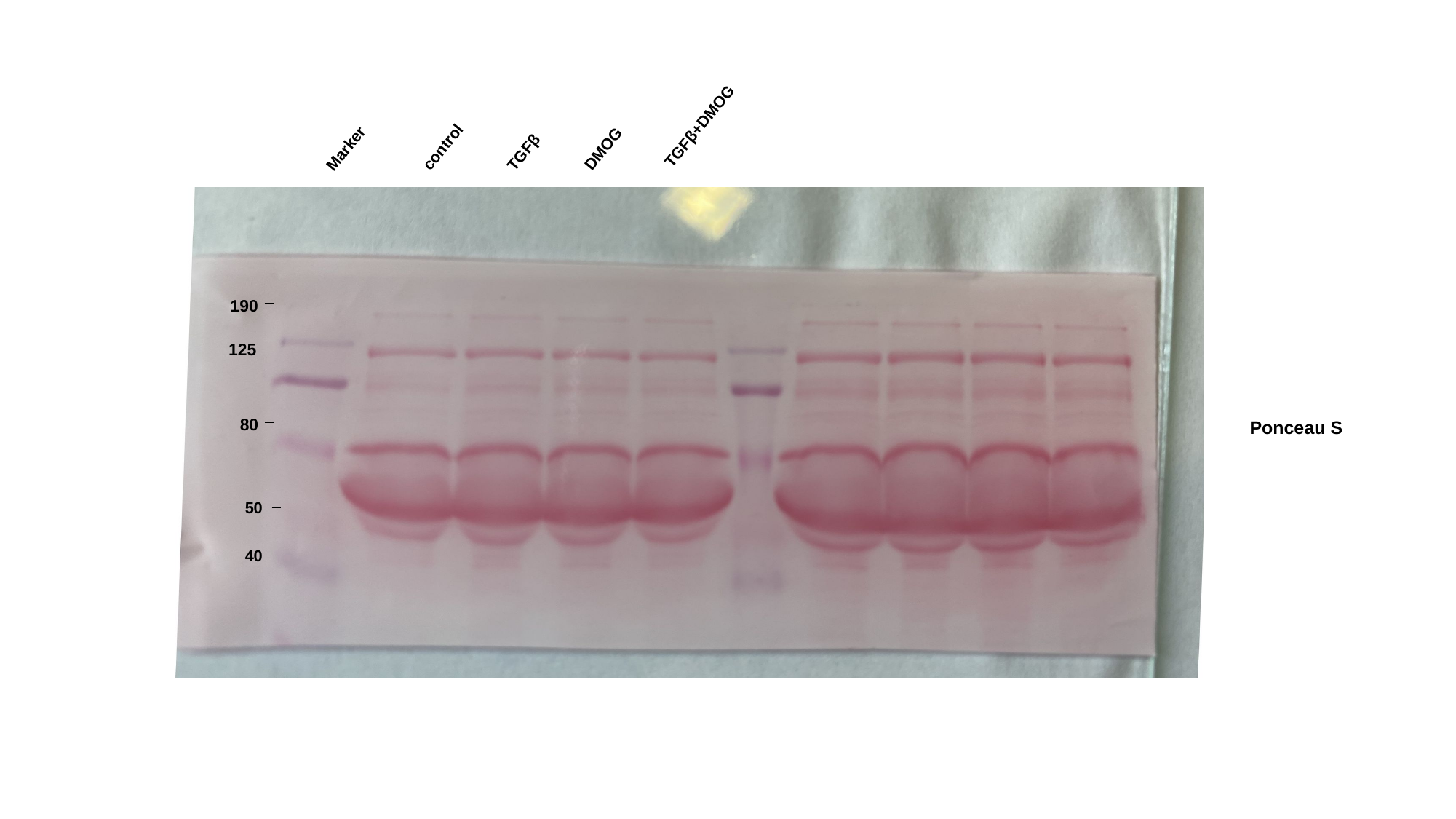

TGFβ+DMOG
DMOG
control
Marker
TGFβ
190
125
80
Ponceau S
50
40

## Slide 2
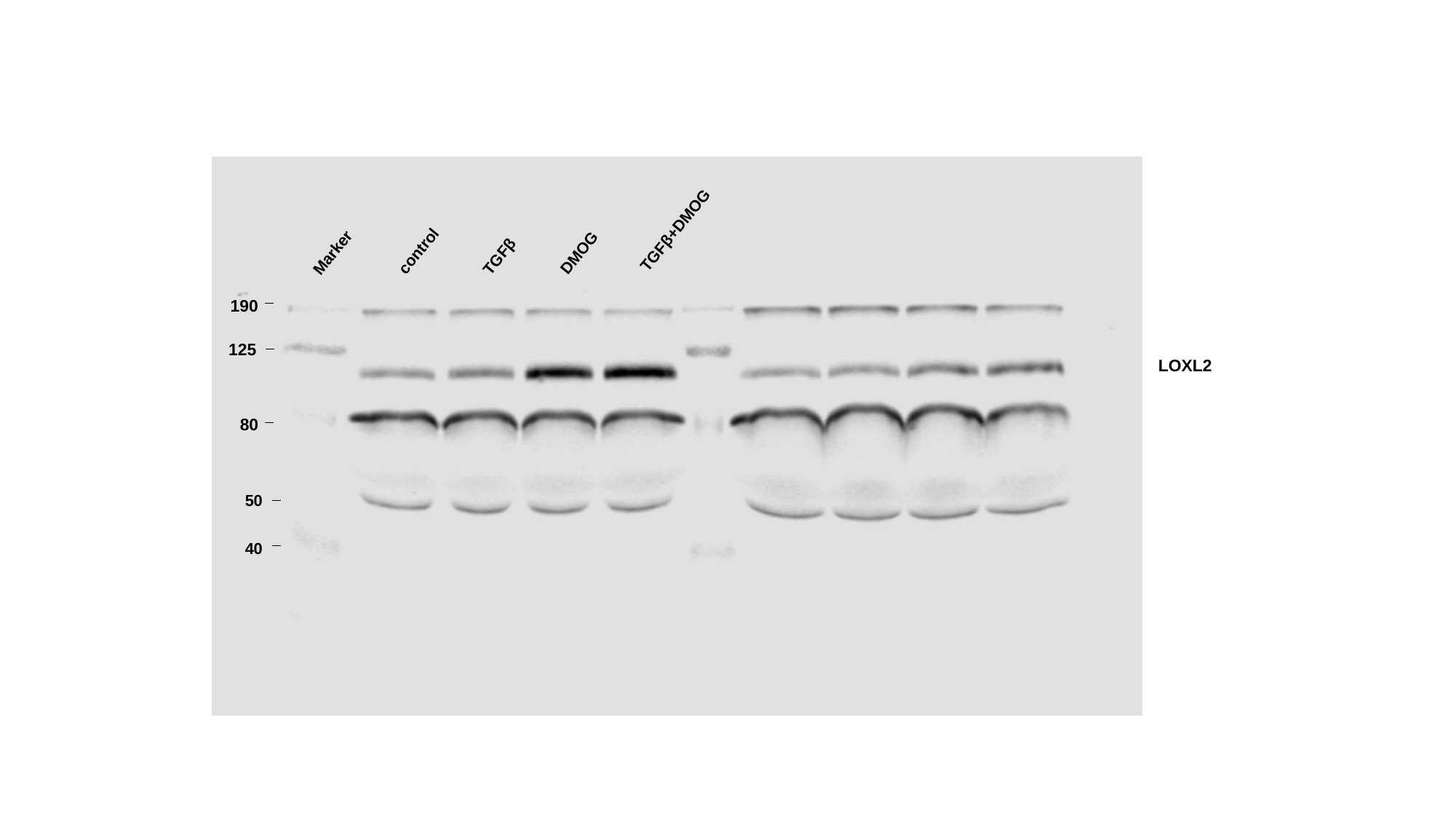

TGFβ+DMOG
DMOG
control
Marker
TGFβ
190
125
LOXL2
80
50
40
