# Supplementary material for: Pseudohypoxic HIF pathway activation dysregulates collagen structure-function in human lung fibrosis
Source: eLife. 2022 Feb 21;11:e69348. doi: 10.7554/eLife.69348 (PMC8860444; doi:10.7554/eLife.69348)
Supplement: Figure 5—figure supplement 1—source data 1. [file elife-69348-fig5-figsupp1-data1.zip › Figure 5ΓÇöfigure supplement 1-source data 1/Figure 5ΓÇöfigure supplement 1-source data 1b/Figure 5ΓÇöfigure supplement 1-source data 1b labelled.pptx]

## Slide 1
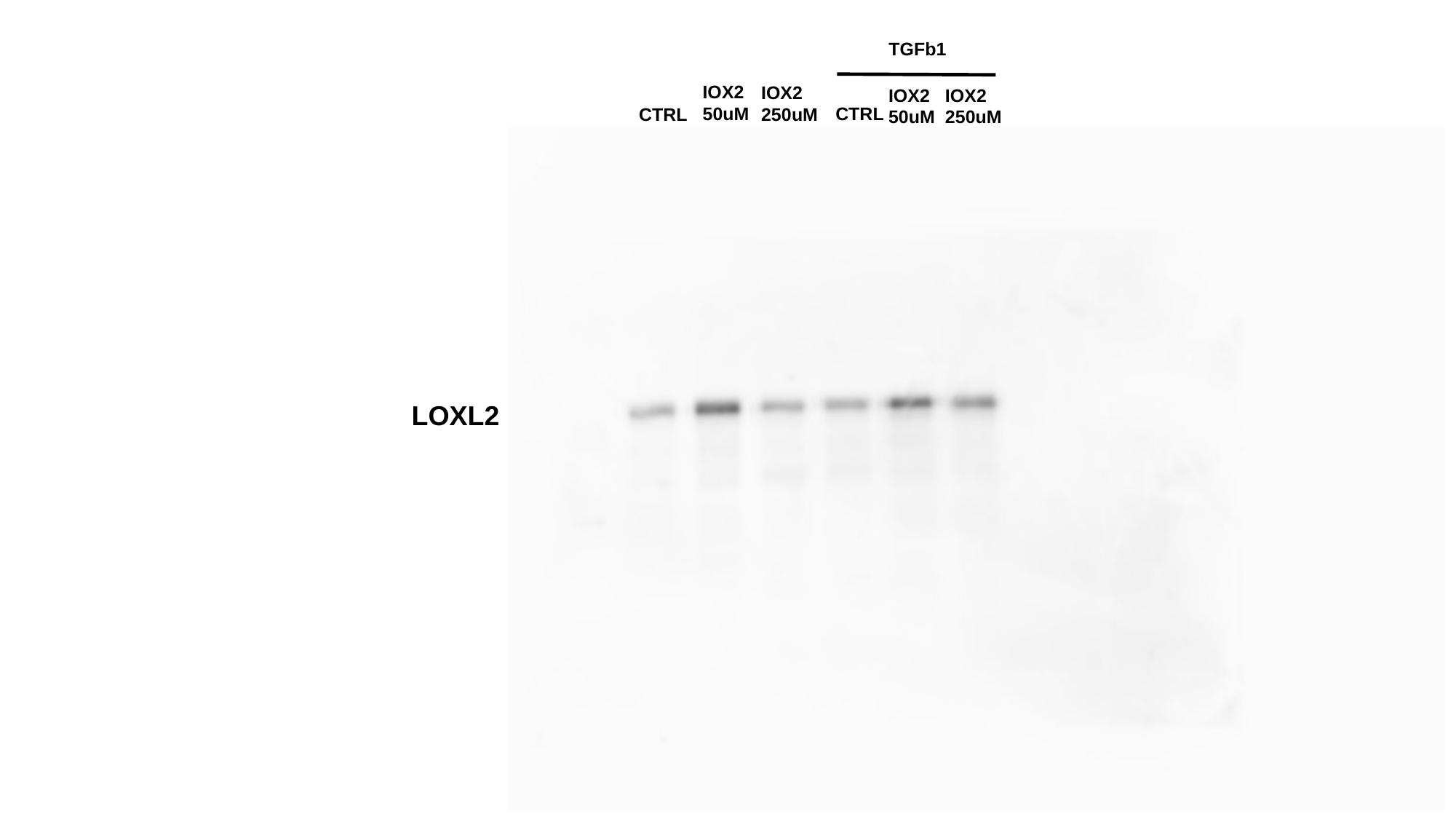

TGFb1
IOX2
50uM
IOX2
250uM
IOX2
50uM
IOX2
250uM
CTRL
CTRL
LOXL2

## Slide 2
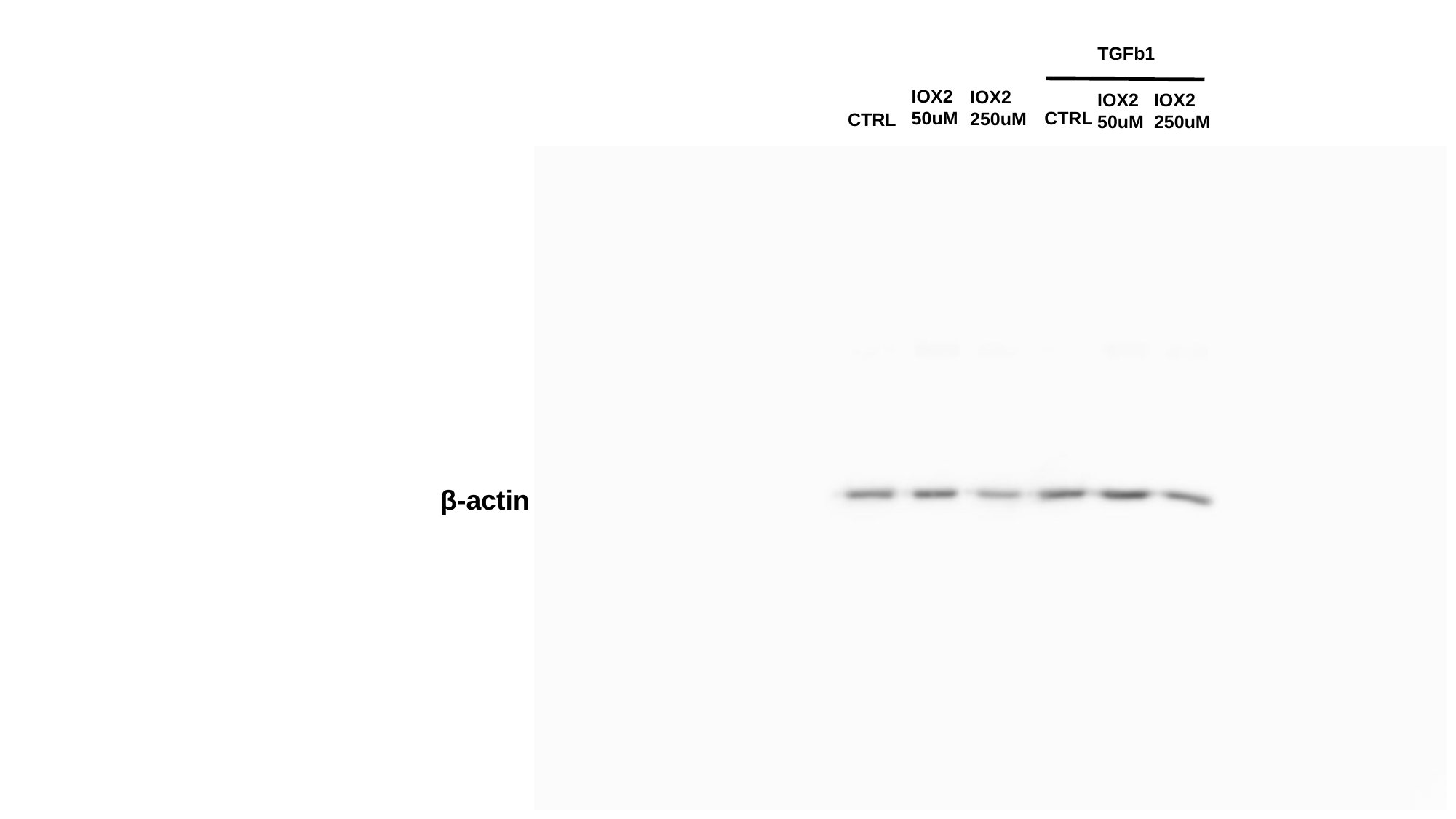

TGFb1
IOX2
50uM
IOX2
250uM
IOX2
50uM
IOX2
250uM
CTRL
CTRL
β-actin
