# Supplementary material for: Pseudohypoxic HIF pathway activation dysregulates collagen structure-function in human lung fibrosis
Source: eLife. 2022 Feb 21;11:e69348. doi: 10.7554/eLife.69348 (PMC8860444; doi:10.7554/eLife.69348)
Supplement: Figure 6—source data 1. [file elife-69348-fig6-data1.zip › Figure 6-source data 1/Figure 6c labelled raw blot.pptx]

## Slide 1
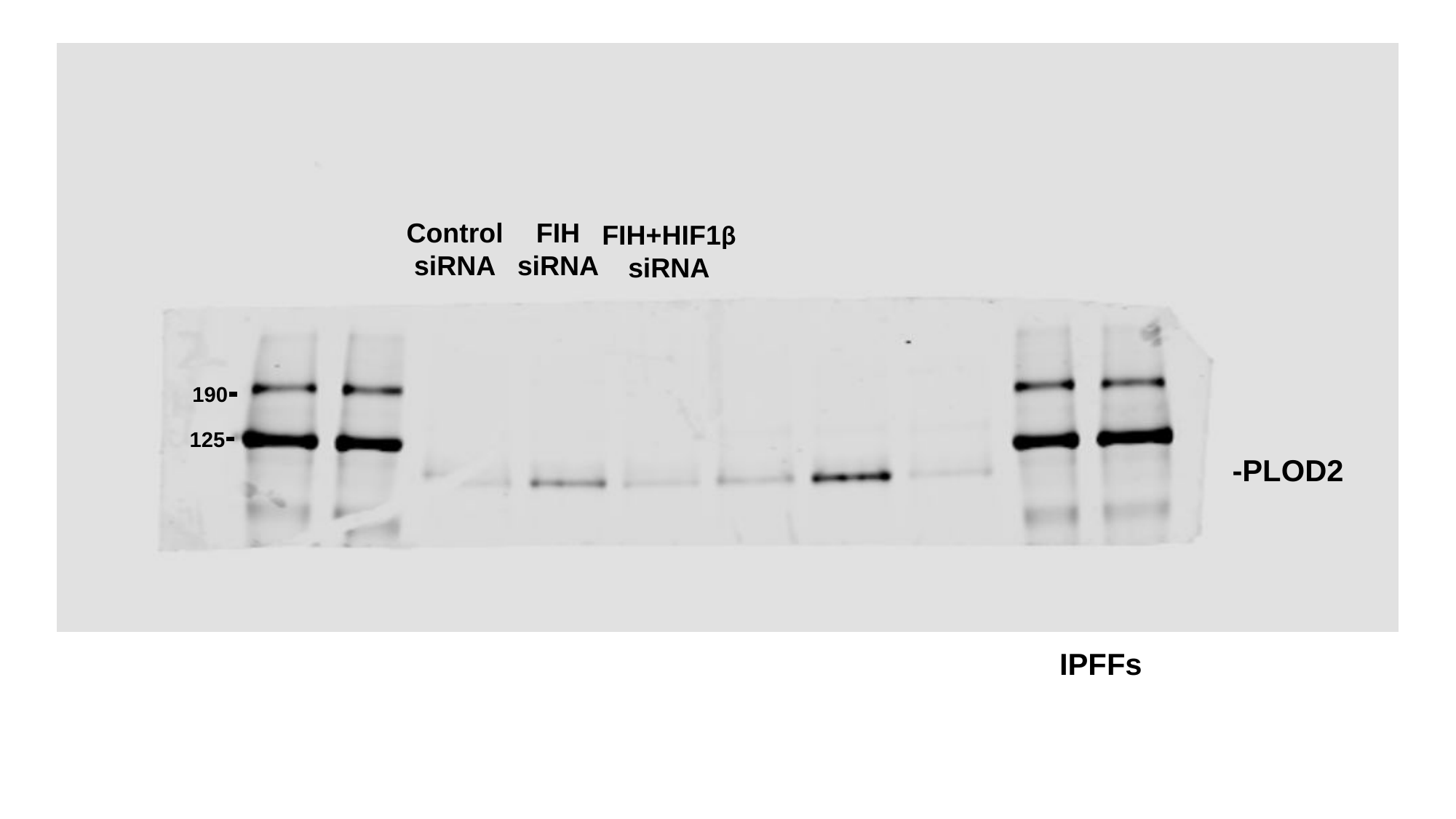

#
Control
siRNA
FIH
siRNA
FIH+HIF1β
siRNA
190
125
-PLOD2
IPFFs

## Slide 2
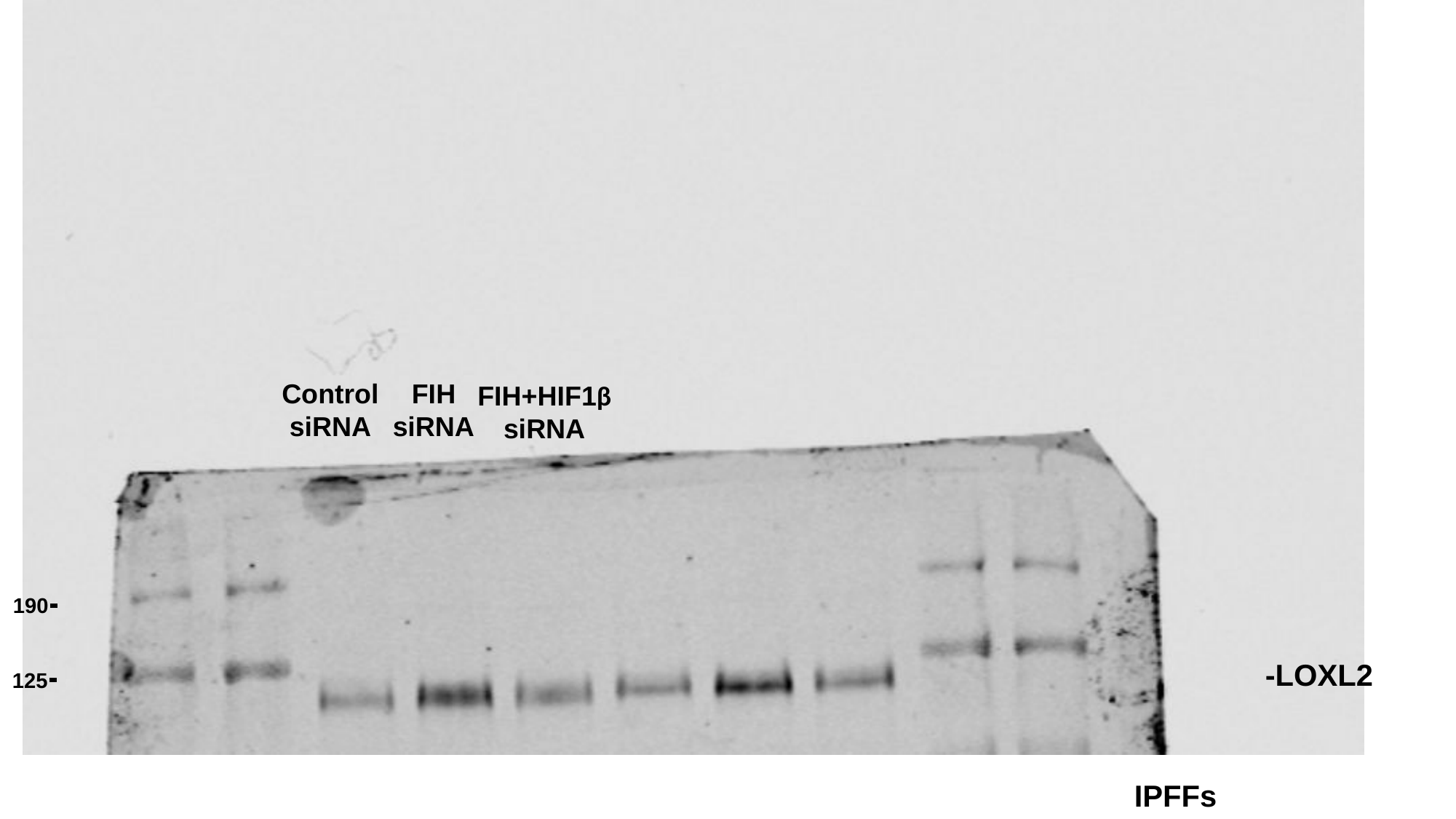

Control
siRNA
FIH
siRNA
FIH+HIF1β
siRNA
190
-LOXL2
125
IPFFs

## Slide 3
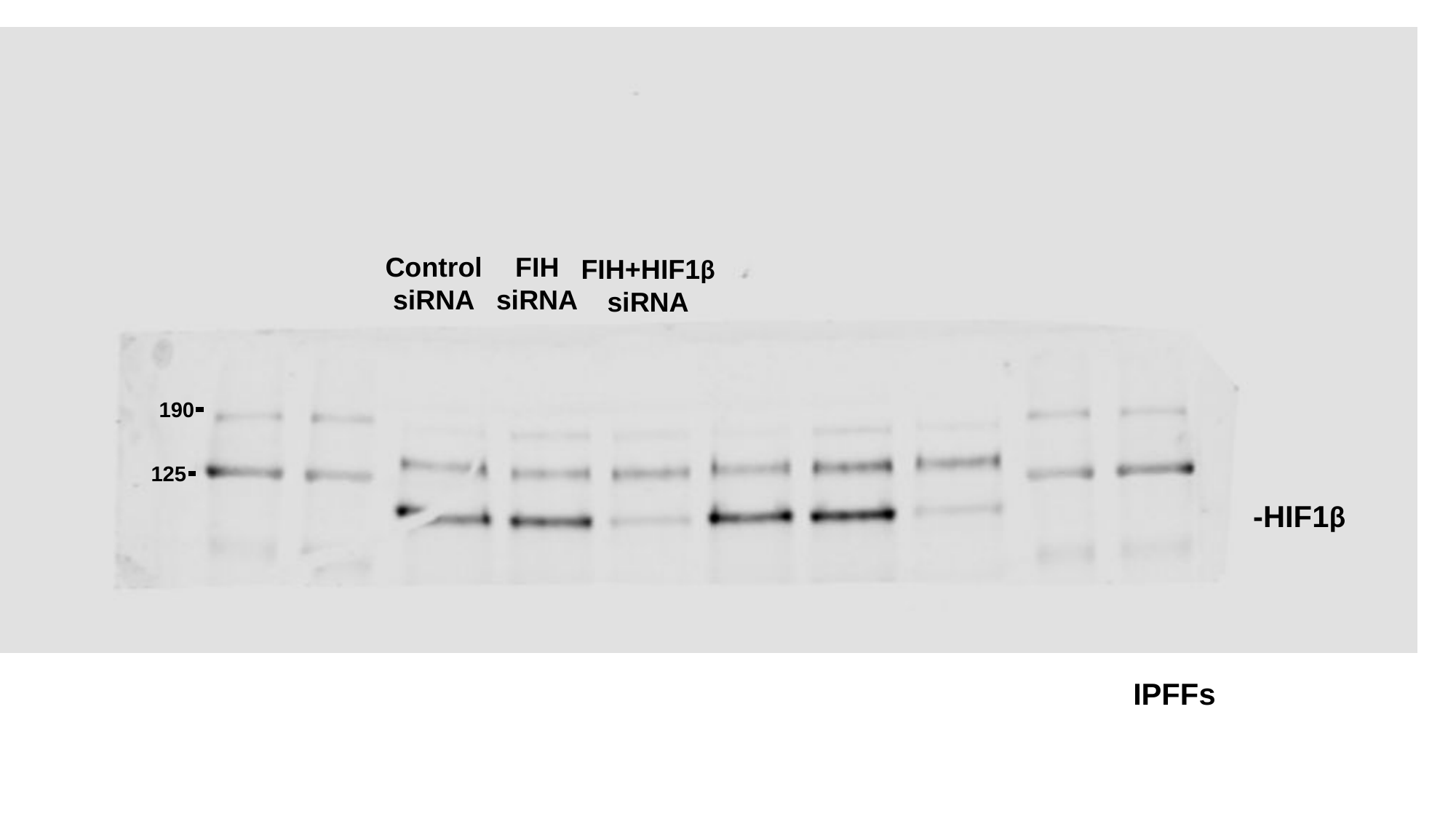

#
Control
siRNA
FIH
siRNA
FIH+HIF1β
siRNA
190
125
-HIF1β
IPFFs

## Slide 4
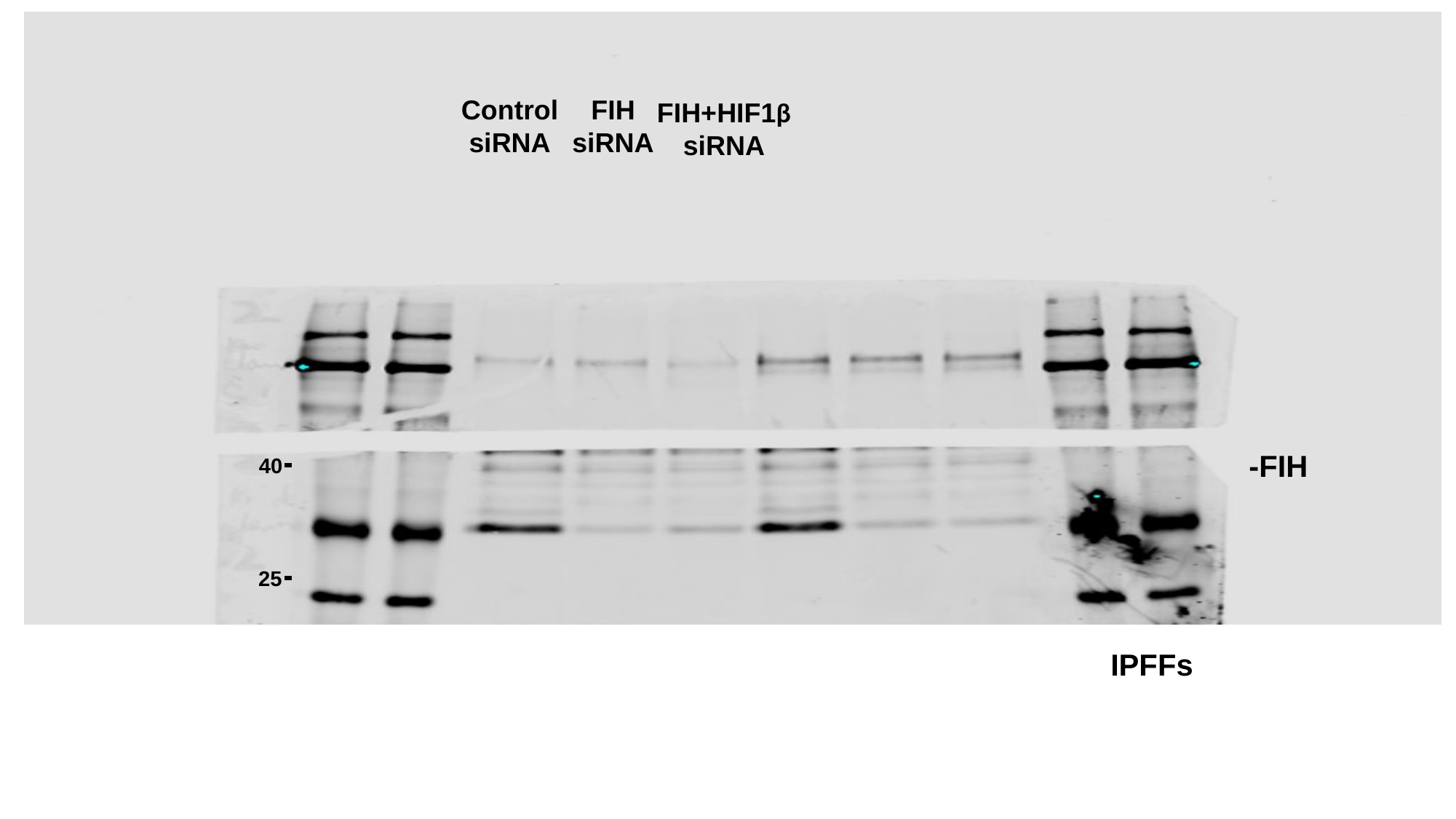

#
Control
siRNA
FIH
siRNA
FIH+HIF1β
siRNA
-FIH
40
25
IPFFs

## Slide 5
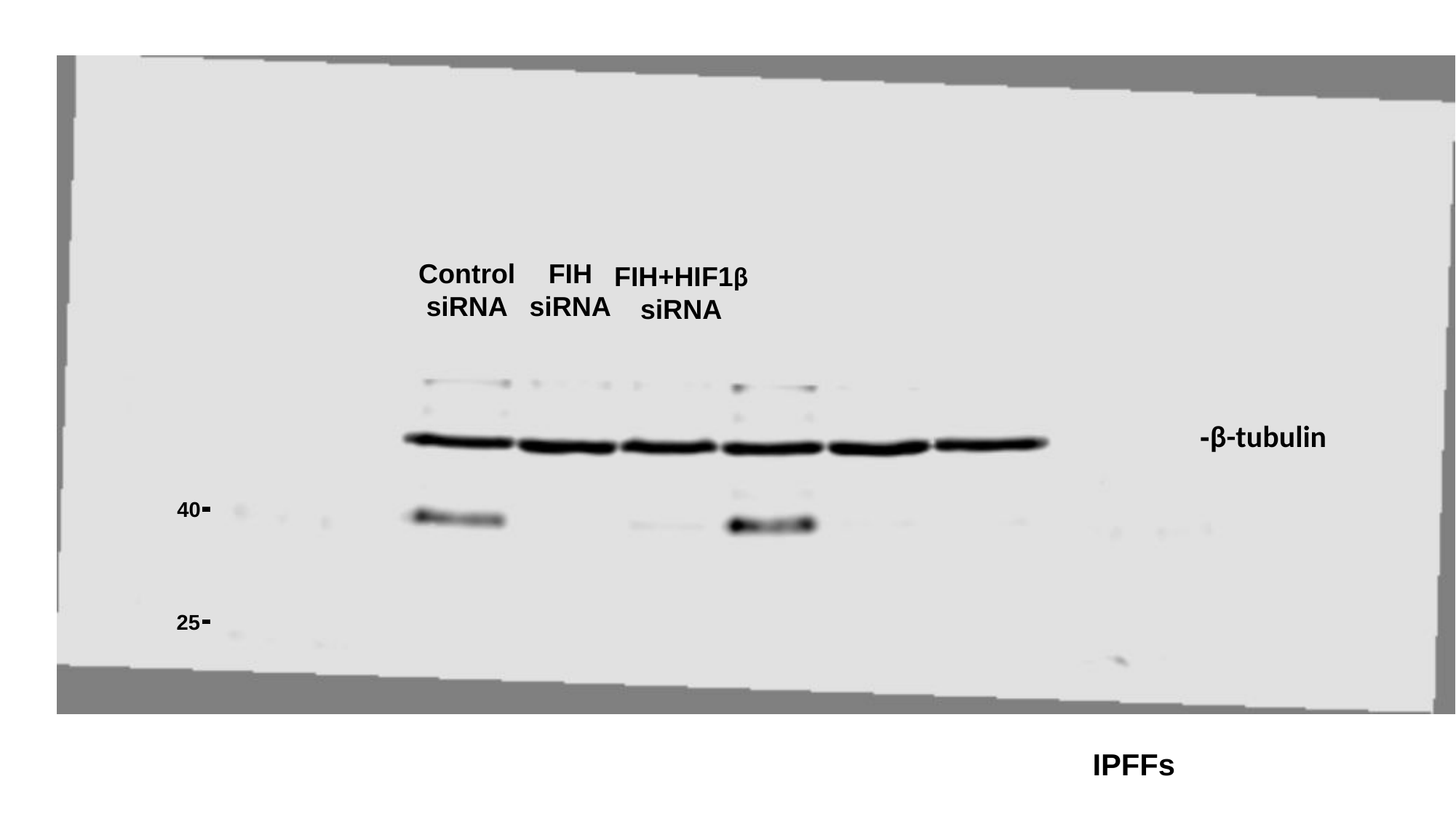

#
Control
siRNA
FIH
siRNA
FIH+HIF1β
siRNA
-β-tubulin
40
25
IPFFs
