# Supplementary material for: Pseudohypoxic HIF pathway activation dysregulates collagen structure-function in human lung fibrosis
Source: eLife. 2022 Feb 21;11:e69348. doi: 10.7554/eLife.69348 (PMC8860444; doi:10.7554/eLife.69348)
Supplement: Supplementary file 1. — (a) Semiquantitative analysis of LOX2 and PLOD2 mRNA expression identified by RNAscope in situ hybridization in cell subtypes in IPF lung tissue (n = 7 donors). FF, fibroblast focus. (b) Fibroblast donor demographic details. [file elife-69348-supp1.docx]

**Supplementary File 1a**. Semiquantitative analysis of *LOX2* and *PLOD2* mRNA expression identified by RNAscope in situ hybridization in cell subtypes in IPF lung tissue (n=7 donors). FF, fibroblast focus

|  | ​Preserved lung pneumocytes | Preserved lung Bronchial epithelium | Alveolar  Macrophages | Smooth muscle | Endothelial  cells | Inflammatory  cells | AT2 overlying FF | Fibroblasts in FF |
| --- | --- | --- | --- | --- | --- | --- | --- | --- |
| *LOX2* |  |  |  |  |  | + | + | +++ |
| *PLOD2* |  |  |  |  |  | + | + | +++ |

**Supplementary File 1b**. Fibroblast donor demographic details

| **Sex** | **Age** | **Smoking history** | **Disease status** |
| --- | --- | --- | --- |
|  |  |  |  |
| M | 68 | never | IPF |
|  |  |  |  |
| M | 71 | never | IPF |
|  |  |  |  |
| F | 66 | ex | IPF |
|  |  |  |  |
| M | 62 | never | IPF |
|  |  |  |  |
| M | 71 | never | IPF |
|  |  |  |  |
| M | 73 | ex | IPF |
|  |  |  |  |
| F | 66 | ex | IPF |
|  |  |  |  |
| F | 60 | never | Normal |
|  |  |  |  |
| M | 71 | ex | Normal |
|  |  |  |  |
| M | 66 | ex | Normal |
|  |  |  |  |
| F | 74 | never | Normal |
|  |  |  |  |
| M | 67 | ex | Normal |
|  |  |  |  |
