# Supplementary material for: Pseudohypoxic HIF pathway activation dysregulates collagen structure-function in human lung fibrosis
Source: eLife. 2022 Feb 21;11:e69348. doi: 10.7554/eLife.69348 (PMC8860444; doi:10.7554/eLife.69348)
Supplement: Supplementary file 2. [file elife-69348-supp2.docx]

**Supplementary File 2. Short interfering RNA (siRNA) oligo sequences.**

Short interfering RNA (siRNA) oligos against HIF1A (HIF1α) (MU-00401805-01-0002), EPAS1 (HIF2α) (MU-004814-01-0002), ARNT (HIF1β) (MU-007207-01-0002) and HIF1AN (FIH) (MU-004073-02-0002) were from Dharmacon, Cambridge, UK.

***ARNT* (HIF1β) *MU-007207-01-0002***

D-007207-01 GAUCAGAUGUCUAACGAUA

D-007207-02 UCAAGGAGAUCGUUUAUUU

D-007207-03 CGAAUGAGGUGUGGCAGUA

D-007207-05 GAUAUGGUACCCACCUGUA

***HIF1A* (HIF1α) *MU-004018-05-0002***

D-004018-01 GGACACAGAUUUAGACUUG

D-004018-03 GAUGGAAGCACUAGACAAA

D-004018-05 CGUGUUAUCUGUCGCUUUG

D-004018-27 GAUGAAAGAAUUACCGAAU

***EPAS1* (HIF2α) *MU-******004814-01-0002***

D-004814-02 GCAAAUGUACCCAAUGAUA

D-004814-03 GAGCGGGACUUCUUCAUGA

D-004814-04 AGACGGAGGUGUUCUAUGA

D-004814-05 ACACAUCUUUGGAUAACGA

***HIF1AN* (FIH) *MU-******004073-02-0002***

D-004073-01 GAAAUUUCAUGAGUUCGUU

D-004073-02 CACAUAGAGUCAUUACUAA

D-004073-04 UCAUGGACUUCUUAGGUUU

D-004073-18 AGUUAUAGCUUCCCGACUA
