# Supplementary material for: Pseudohypoxic HIF pathway activation dysregulates collagen structure-function in human lung fibrosis
Source: eLife. 2022 Feb 21;11:e69348. doi: 10.7554/eLife.69348 (PMC8860444; doi:10.7554/eLife.69348)
Supplement: Source code 1. [file elife-69348-code1.zip › Source Code 1.docx]

*### clean environment*

rm(list = ls())

*### load R packages*

library(GEOquery)

library(GSVA)

*### HIF gene list*

Hypoxia_list<-list(c('NDRG1','ENO1','VEGFA','MRPS17','TPI1','CDKN3',

'MIF','LDHA','ALDOA','TUBB6','PGAM1','SLC2A1','P4HA1','ACOT7','ADM'))

*####GSE40839 ####*

setwd('/Users/lefan/Rcode/IPF-singlecell/MARK/GSE40839/')

GSE40839<-getGEO('GSE40839',destdir = '.') *### download GSE40839 data and annotation file*

GSE40839<-ReadAffy() *### read all CEL files in this working directory*

GSE40839_data<-affy::rma(GSE40839) *### Normalize GSE40839*

GSE40839_data<-exprs(GSE40839_data) *### Extract expression value*

bb<-"./GPL96.soft" *### read annotation file*

bb_nn <- grep("^[^#!^]", readLines(bb))[1] – 1

pfinfo_bb <- read.table(bb, sep = "\t", quote = "", header = TRUE, skip = bb_nn, fill = TRUE) *### extract probe ID information*

pfinfo_bb <- pfinfo_bb[, c(1,11)] *### extract Probe ID and gene symbol*

write.csv(pfinfo_bb,'pfinfo_bb.csv') *### save file*

pfinfo_bb<-read.csv('pfinfo_bb.csv') *### load file*

pfinfo_bb<-pfinfo_bb[,1:2]

GSE40839_data<-as.data.frame(GSE40839_data)

GSE40839_data$ID<-rownames(GSE40839_data)

GSE40839_data<-merge(GSE40839_data,pfinfo_bb,by='ID') *### merge expression matrix with gene symbol by Probe ID*

GSE40839_data<-aggregate(x=GSE40839_data[,2:22],by=list(GSE40839_data$Gene.Symbol),FUN=median) *### Multiple probes relating to the same gene were deleted and summarised as the median value for further analysis*

rownames(GSE40839_data)<-GSE40839_data$Group.1

GSE40839_data<-GSE40839_data[-1,-1]

GSE40839_data<-as.matrix(GSE40839_data)

*### calculate HIF score via GSVA*

Hypoxia_score_GSE40839<-gsva(GSE40839_data,Hypoxia_list, mx.diff=1,kcdf= "Gaussian")

*#### GSE73854 ####*

setwd('/Users/lefan/Rcode/IPF-singlecell/GSE73854/')

GSE73854<-getGEO('GSE73854',destdir = '.') *### download GSE73854 data and annotation file*

GSE73854<-GSE73854[[1]]

GSE73854_data<-exprs(GSE73854) *### Extract expression value*

colnames(GSE73854_data)<-c('Stable_IPF_1','Stable_IPF_2','Stable_IPF_3','Stable_IPF_4',

'Progressive_IPF_1','Progressive_IPF_2','Progressive_IPF_3','Progressive_IPF_4') *### rename sample*

GPL570<-'./GPL570.soft' " *### read annotation file*

GPL570_nn <- grep("^[^#!^]", readLines(GPL570))[1] – 1

GPL570_dd <- read.table(GPL570, sep = "\t", quote = "", header = TRUE, skip = GPL570_nn, fill = TRUE) *### extract probe ID information*

GPL570_dd <- GPL570_dd[, c(1,11)] *### extract Probe ID and gene symbol*

#write.csv(GPL570_dd,'GPL570.csv') *### save file*

#GPL570_dd<-read.csv('GPL570.csv',header = T,sep = ',') *### load file*

GPL570_dd<-na.omit(GPL570_dd) *### remove NA values*

GSE73854_data<-as.data.frame(GSE73854_data)

GSE73854_data$ID<-rownames(GSE73854_data)

GSE73854_data<-merge(GSE73854_data,GPL570_dd,by='ID') *### merge expression matrix with gene symbol by Probe ID*

GSE73854_data_new<-aggregate(x=GSE73854_data[,2:9],by=list(GSE73854_data$Gene.Symbol),FUN=median)

*### Multiple probes relating to the same gene were deleted and summarised as the median value for further analysis*

rownames(GSE73854_data_new)<-GSE73854_data_new$Group.1

GSE73854_data_new<-GSE73854_data_new[-1,-1]

GSE73854_data_new<-as.matrix(GSE73854_data_new)

### calculate HIF score via GSVA

Hypoxia_score_GSE73854<-gsva(GSE73854_data_new,Hypoxia_list , mx.diff=1, kcdf= "Gaussian"))
