# Supplementary material for: Pseudohypoxic HIF pathway activation dysregulates collagen structure-function in human lung fibrosis
Source: eLife. 2022 Feb 21;11:e69348. doi: 10.7554/eLife.69348 (PMC8860444; doi:10.7554/eLife.69348)
Supplement: Source code 2. [file elife-69348-code2.zip › Source Code 2.docx]

*### clean environment*

rm(list = ls())

*### load R packages*

library(GEOquery)

library(GSVA)

*### HIF gene list*

Hypoxia_list<-list(c('NDRG1','ENO1','VEGFA','MRPS17','TPI1','CDKN3',

'MIF','LDHA','ALDOA','TUBB6','PGAM1','SLC2A1','P4HA1','ACOT7','ADM'))

*### Hallmark TGFB gene list*

TGFB_list<-list(c("ACVR1","APC","ARID4B","BCAR3","BMP2","BMPR1A","BMPR2","CDH1","CDK9","CDKN1C","CTNNB1", "ENG","FKBP1A","FNTA","FURIN","HDAC1","HIPK2","ID1","ID2","ID3","IFNGR2","JUNB","KLF10","LEFTY2","LTBP2","MAP3K7","NCOR2","NOG","PMEPA1","PPM1A","PPP1CA","PPP1R15A","RAB31","RHOA","SERPINE1","SKI","SKIL","SLC20A1","SMAD1","SMAD3","SMAD6","SMAD7","SMURF1","SMURF2", "SPTBN1","TGFB1","TGFBR1","TGIF1","THBS1","TJP1","TRIM33","UBE2D3","WWTR1","XIAP"))

*#### PLOD2&LOXL2 in FF####*

FF<-read.table(' Fibroblast_Foci_New.txt',header = T,sep = '\t') ### load RNA-seqfile

rownames(FF)<-FF$Gene

FF<-FF[,-1]

FF<-as.matrix(FF)

*### gene list score*

gsva_Hypoxia <- gsva(FF,Hypoxia_list , mx.diff=1)

HIF1A<-as.data.frame((gsva_Hypoxia))

gsva_TGFB <- gsva(FF,TGFB_list , mx.diff=1)

TGFB <-as.data.frame((gsva_TGFB))

*### LOXL2_PLOD2 score*

LOXLPLOD_list<-list(c('PLOD2','LOXL2'))

gsva_LOXLPLOD <- gsva(FF,LOXLPLOD_list , mx.diff=1)

LOXLPLOD<-as.data.frame((gsva_LOXLPLOD))

*### Correlation and plot*

cor.test(as.numeric(HIF1A[1,]),as.numeric(LOXLPLOD[1,]),method = "spearman")

plot(as.numeric(HIF1A[1,]),as.numeric(LOXLPLOD[1,]))

cor.test(as.numeric(TGFB [1,]),as.numeric(LOXLPLOD[1,]),method = "spearman")

plot(as.numeric(TGFB [1,]),as.numeric(LOXLPLOD[1,]))
