# Supplementary material for: Pseudohypoxic HIF pathway activation dysregulates collagen structure-function in human lung fibrosis
Source: eLife. 2022 Feb 21;11:e69348. doi: 10.7554/eLife.69348 (PMC8860444; doi:10.7554/eLife.69348)
Supplement: Source code 3. [file elife-69348-code3.zip › Source Code 3.docx]

library(magrittr)

library(tidyverse)

library(Matrix)

library(Seurat)

library(ggplot2)

library(data.table)

library(ggrepel)

library(ggsci)

library(GSVA)

library(GO.db)

library(topGO)

library(viridis)

#Data taken from GSE135893. Fibroblast data is taken by extracting different fibroblast populations (fibroblasts, myofibroblasts, PLIN2+ fibroblasts and

#HAS1-high fibroblasts.)

# Read in Data ------------------------------------------------------------

str(data <- readMM("GSE135893_matrix.mtx"))

metadata <- read.csv("GSE135893_IPF_metadata.csv", stringsAsFactors = F)

barcodes <- scan("GSE135893_barcodes.tsv", character())

genes <- scan("GSE135893_genes.tsv", character())

colnames(data) <- barcodes

rownames(data) <- genes

data <- data[,match(metadata$X, colnames(data))]

meta.unq <- unique(metadata$population)

meta.mesenchymal <- grep("Mesenchymal", metadata$population)

meta.mesenchymal <- metadata[meta.mesenchymal,]

meta.fibro <- grep("Muscle", meta.mesenchymal$celltype)

meta.fibro <- meta.mesenchymal[-meta.fibro,]

#IPF.inds <- grep("IPF", meta.fibro$Diagnosis)

#meta.fibro <- meta.fibro[IPF.inds,]

meso.inds <- grep("Mesothelial", meta.fibro$celltype)

#write.csv(meta.mesenchymal, file = "Kropski_mesenchymal.csv")

meta.fibro <- meta.fibro[-meso.inds,]

barcodes.match <- match(meta.fibro$X, colnames(data))

fibro.data <- data[,barcodes.match]

dim(fibro.data)

# Only use IPF and control cells going forwards ---------------------------

ipf.ctrl <- which(meta.fibro$Diagnosis == "Control" | meta.fibro$Diagnosis == "IPF")

ipf.ctrl.meta <- meta.fibro[ipf.ctrl,]

ipf.ctrl.fibro <- fibro.data[,ipf.ctrl]

rownames(ipf.ctrl.meta) <- ipf.ctrl.meta$X

# Seurat analysis to generate t-SNE embeddings for GSVA plots -------------

fibroblasts <- CreateSeuratObject(counts = ipf.ctrl.fibro, project = "Kropski", meta.data = ipf.ctrl.meta)

pbmc <- NormalizeData(object = fibroblasts, normalization.method = "LogNormalize", scale.factor = 10000)

pbmc <- FindVariableFeatures(object = pbmc, selection.method = "vst", nfeatures = 2000)

# Identify the 10 most highly variable genes

top10 <- head(x = VariableFeatures(object = pbmc), 10)

plot1 <- VariableFeaturePlot(object = pbmc)

plot2 <- LabelPoints(plot = plot1, points = top10, repel = TRUE)

CombinePlots(plots = list(plot1, plot2))

all.genes <- rownames(x = pbmc)

pbmc <- ScaleData(object = pbmc, features = all.genes)

pbmc <- RunPCA(object = pbmc, features = VariableFeatures(object = pbmc))

print(x = pbmc[["pca"]], dims = 1:5, nfeatures = 5)

VizDimLoadings(object = pbmc, dims = 1:2, reduction = "pca")

DimPlot(object = pbmc, reduction = "pca")

DimHeatmap(object = pbmc, dims = 1:15, cells = 500, balanced = TRUE)

# pbmc <- JackStraw(object = pbmc, num.replicate = 100)

# pbmc <- ScoreJackStraw(object = pbmc, dims = 1:20)

# JackStrawPlot(object = pbmc, dims = 1:20)

ElbowPlot(object = pbmc)

clusters <- ipf.ctrl.meta$celltype

unique(clusters)

pbmc@meta.data$new.clusters <- clusters

#PLot TSNE - first use the inbuilt tsneplot() function, then get embeddings and make a prettier ggplot2 cluster plot.

pbmc=RunTSNE(pbmc,dims.use = 1:15,max_iter=2000)

TSNEPlot(object = pbmc, group.by = "new.clusters")

#writeMM(fibro.data, file = "fibro_data.mtx")

tsne.embed <- as.data.frame(pbmc@reductions$tsne@cell.embeddings, stringsAsFactors = F)

# Save and then read in tsne embeddings -----------------------------------

tsne.embed$cluster <- clusters

write.csv(tsne.embed, file = "kropski_fibro_tsne_embeddings.csv")

tsne.embed <- read.csv("kropski_fibro_tsne_embeddings.csv", row.names = 1, stringsAsFactors = F)

tsne.ipf.ctrl <- tsne.embed[ipf.ctrl,]

#Generate normalised matrix for use in GSVA analysis

fibroblasts <- NormalizeData(object = fibroblasts, normalization.method = "LogNormalize", scale.factor = 10000)

norm.data <- as.matrix(fibroblasts@assays$RNA@data)

hist(norm.data[,2000], breaks = 10)

norm.data.reduced <- apply(norm.data,1, function(x){

sum(x) !=0

})

norm.data.reduced <- norm.data[norm.data.reduced,]

#Oxidative stress genes

Oxidative_stress <- c("CAT", "CYBB", "CYP1A1", "FOS", "GCLC", "GPX1", "GPX3", "GSR", "GSTT2", "HMOX1", "JUNB", "MAOA", "MAPK10",

"MAPK14", "MGST1", "MT1X", "NFE2L2", "NFIX", "NFKB1", "NOX1", "NOX3", "NOX4", "NOX5", "NQO1", "SOD1", "SOD2",

"SOD3", "SP1", "TXN2", "TXNRD1", "TXNRD2", "UGT1A6", "XDH")

os.inds <- match(Oxidative_stress, rownames(norm.data.reduced))

os.inds <-os.inds[!is.na(os.inds)]

os.dat <- norm.data.reduced[os.inds,]

t.os <- t(os.dat)

df.os <- as.data.frame(t.os)

df.os$celltype <- NULL

os.diag <- aggregate(df.os, by = list(ipf.ctrl.meta$Diagnosis), FUN = mean)

rownames(os.diag) <- os.diag$Group.1

os.diag$Group.1 <- NULL

os.diag.hm <- t(os.diag)

os.diag.hm.mat <- as.matrix(os.diag.hm)

os.diag.hm <- as.data.frame(os.diag.hm)

os.diag.hm$diff <- os.diag.hm$IPF - os.diag.hm$Control

#Get up and downregulated Oxidative Stress genes

os.upreg <- os.diag.hm[which(os.diag.hm$diff > 0),]

os.downreg <- os.diag.hm[which(os.diag.hm$diff <0),]

os.up.genes <- rownames(os.upreg)

os.down.genes <- rownames(os.downreg)

os.genes <- list(os.up.genes, os.down.genes)

os.gsva <- gsva(expr = norm.data.reduced, gset.idx.list = os.genes) #Run GSVA for up and downregulated OS genes

rownames(os.gsva) <- c("Upregulated Oxidative Stress", "Downregulated Oxidative Stress")

os.gsva.t <- as.data.frame(t(os.gsva) )

# GSVA --------------------------------------------------------------------

#Perform GSVA for hypoxia geneset

Hypoxia <- c("VEGFA", "SLC2A1", "PGAM1", "ENO1", "LDHA", "TPI1", "P4HA1", "MRPS17", "CDKN3", "ADM", "NDRG1", "TUBB6", "ALDOA", "MIF", "ACOT7")

Hyp.list <- list(Hypoxia)

Hyp.gsva <- gsva(expr = norm.data.reduced, gset.idx.list = Hyp.list) #Run GSVA

clust.mean.ipf.ctrl <- aggregate(tsne.embed[,1:2], by = list(Cluster = tsne.embed$cluster), mean)[,1:3] # Get cluter mean coordinates for cluster labels

cols <- viridis()

# Plots -------------------------------------------------------------------

#T-SNE plot for hypoxia GSVA

ggplot(data = tsne.embed, aes(x = tSNE_1, y = tSNE_2)) +

geom_point(size = 1.4, aes(color = Hyp.gsva)) +

geom_point(size = 3, alpha = 1/100) +

xlab("TSNE 1") +

ylab("TSNE 2") +

labs(color='GSVA Score') +

ggtitle("GSVA: HIF score") +

theme_bw() +

theme(panel.border = element_blank()) +

theme(axis.line = element_line(colour = "black", size = 2)) +

theme(panel.grid = element_blank())+

theme(text = element_text(size=20)) +

geom_text_repel(data=clust.mean.ipf.ctrl, aes(x=tSNE_1, y=tSNE_2, label = Cluster), seed = 234, size = 7,

colour = "black", box.padding = 1, force = 1, max.overlaps = Inf, min.segment.length = 0, segment.size = 1,

segment.colour = "grey10", ylim = c(-25, 50)) +

scale_color_viridis()

#T-SNE plot for upregulated oxidative stress GSVA

ggplot(data = tsne.embed, aes(x = tSNE_1, y = tSNE_2)) +

geom_point(size = 1.4, aes(color = os.gsva.t$`Upregulated Oxidative Stress`)) +

geom_point(size = 3, alpha = 1/100) +

xlab("TSNE 1") +

ylab("TSNE 2") +

labs(color='GSVA Score') +

ggtitle("GSVA: Upregulated oxidative stress") +

theme_bw() +

theme(panel.border = element_blank()) +

theme(axis.line = element_line(colour = "black", size = 2)) +

theme(panel.grid = element_blank())+

theme(text = element_text(size=20)) +

geom_text_repel(data=clust.mean.ipf.ctrl, aes(x=tSNE_1, y=tSNE_2, label = Cluster), seed = 234, size = 7,

colour = "black", box.padding = 1, force = 1, max.overlaps = Inf, min.segment.length = 0, segment.size = 1,

segment.colour = "grey10", ylim = c(-25, 50)) +

scale_color_viridis()

#Correlation plot for HIF vs oxidative stress

GSVA.all <- os.gsva.t

GSVA.all$HIF <- t(Hyp.gsva)

colnames(GSVA.all) <- c("UOS", "DOS", "HIF")

ggplot(GSVA.all, aes(x = HIF, y = UOS)) +

geom_point(colour = "grey60") +

geom_smooth(method='lm', se = F, colour = "black", size = 2) +

xlab("Hypoxia GSVA Score") +

ylab("Oxidative stress GSVA Score") +

ggtitle("Correlation plot of HIF score vs \n Upregulated Oxidative stress score") +

labs(fill = "") +

theme_bw() +

theme(panel.border = element_blank()) +

theme(axis.line = element_line(colour = "black", size = 2)) +

theme(panel.grid = element_blank()) +

theme(text = element_text(size=24)) +

annotate(geom="label", x=0.2, y=-0.61, label=paste("Coeff: = ",cor(x = GSVA.all$HIF, y = GSVA.all$UOS)

%>% round(2), ", P < 2.2e-16"),

color="black", alpha = 0.6, size = 6.5)

# T-SNE plot for different clusters

ggplot(data = tsne.embed, aes(x = tSNE_1, y = tSNE_2)) +

geom_point(size = 1.4, aes(color = cluster)) +

geom_point(size = 3, alpha = 1/100) +

xlab("TSNE 1") +

ylab("TSNE 2") +

labs(color='Cell Type') +

ggtitle("GSVA: HIF score") +

theme_bw() +

theme(panel.border = element_blank()) +

theme(axis.line = element_line(colour = "black", size = 2)) +

theme(panel.grid = element_blank())+

theme(text = element_text(size=20)) +

geom_text_repel(data=clust.mean.ipf.ctrl, aes(x=tSNE_1, y=tSNE_2, label = Cluster), seed = 234, size = 7,

colour = "black", box.padding = 1, force = 1, max.overlaps = Inf, min.segment.length = 0, segment.size = 1,

segment.colour = "grey10", ylim = c(-25, 50)) +

scale_color_viridis(discrete = T)

# T-SNE plot for different diseases

tsne.embed$Disease <- ipf.ctrl.meta$Diagnosis

ggplot(data = tsne.embed, aes(x = tSNE_1, y = tSNE_2)) +

geom_point(size = 1.4, aes(color = Disease)) +

geom_point(size = 3, alpha = 1/100) +

xlab("TSNE 1") +

ylab("TSNE 2") +

labs(color='Diagnosis') +

ggtitle("GSVA: HIF score") +

theme_bw() +

theme(panel.border = element_blank()) +

theme(axis.line = element_line(colour = "black", size = 2)) +

theme(panel.grid = element_blank())+

theme(text = element_text(size=20)) +

geom_text_repel(data=clust.mean.ipf.ctrl, aes(x=tSNE_1, y=tSNE_2, label = Cluster), seed = 234, size = 7,

colour = "black", box.padding = 1, force = 1, max.overlaps = Inf, min.segment.length = 0, segment.size = 1,

segment.colour = "grey10", ylim = c(-25, 50)) +

scale_color_manual(values = c("#29AF7FFF", "#FDE725FF"))

#Bar chart for HIF score and oxidative stress by cell type

meta.gs <- data.frame(ipf.ctrl.meta, os.gsva.t, t(Hyp.gsva))

celltypes <- unique(meta.gs$celltype)

gs.meta.reduced <- which(meta.gs$Diagnosis == "Control" & meta.gs$celltype == "PLIN2+ Fibroblasts") #Remove PLIN2+ cells from control as too few of them.

gs.meta.reduced <- meta.gs[-gs.meta.reduced,]

gs.meta <- gs.meta.reduced[,match("Upregulated.Oxidative.Stress", colnames(gs.meta.reduced))]

gs.meta <-data.frame(gs.meta.reduced$Diagnosis, gs.meta.reduced$celltype, gs.meta)

geneset.bar <- function(geneset) {

gs.meta <- gs.meta.reduced[,match(geneset, colnames(gs.meta.reduced))]

gs.meta <- data.frame(gs.meta.reduced$Diagnosis, gs.meta.reduced$celltype, gs.meta)

ggplot(gs.meta, aes(x= gs.meta.reduced.Diagnosis, y = gs.meta, fill = gs.meta.reduced.celltype)) +

#geom_point(position = position_dodge(width = 0.5), size = 0.5) +

#geom_col(position = position_dodge(width = 0.5), width = 0.4) +

xlab("Diagnosis") +

ylab("Enrichment Score") +

ggtitle("GSVA: HIF score") +

labs(fill = "") +

theme_bw() +

theme(panel.border = element_blank()) +

theme(axis.line = element_line(colour = "black", size = 1.2)) +

theme(panel.grid = element_blank()) +

theme(text = element_text(size=18)) +

#scale_fill_manual(values = c("cornflowerblue", "orangered2", "green", "orange")) +

#scale_colour_manual(values = c("cornflowerblue", "orangered2", "green", "orange")) +

theme(axis.text.x = element_text(angle = 45, hjust = 1)) +

stat_summary(fun = mean,

geom="col",position = position_dodge(width = 0.7), width = 0.5, size = 1, colour = "black") +

stat_summary(fun.data=mean_se,

geom="errorbar", width=0.2, position = position_dodge(width = 0.7), colour = "black", size = 1) +

geom_hline(yintercept = 0, size = 1)

}

geneset.bar("Upregulated.Oxidative.Stress")

geneset.bar("t.Hyp.gsva.")
